# Supplementary material for: Engineering Enzyme‐Cleavable Oligonucleotides by Automated Solid‐Phase Incorporation of Cathepsin B Sensitive Dipeptide Linkers
Source: Angew Chem Int Ed Engl. 2022 Feb 10;61(13):e202114016. doi: 10.1002/anie.202114016 (PMC9306542; doi:10.1002/anie.202114016)
Supplement: Supplementary file 1 — Supporting Information [file ANIE-61-0-s001.pdf]

Angewandte Chemie
Eine Zeitschrift der Gesellschaft Deutscher Chemiker

Supporting Information

# Engineering Enzyme-Cleavable Oligonucleotides by Automated Solid-Phase Incorporation of Cathepsin B Sensitive Dipeptide Linkers

*C. Jin, A. H. El-Sagheer, S. Li, K. A. Vallis, W. Tan, T. Brown**

## Table of Contents

1. List of abbreviations……………………………………………………………………………………2

2. Materials……………………………………………………………………………………………………3

3. Experimental section……………………………………………………………………………………3

4. Tables and figures…………………………………………………………………………………………5

5. Synthesis of phosphoramidites………………………………………………………………………30

6. NMR spectra………………………………………………………………………………………………45

7. Author contributions……………………………………………………………………………………66

## 1. List of abbreviations

HPLC: high-performance liquid chromatography

LC-MS: liquid chromatography-mass spectrometry

DMT: 4,4'-dimethoxytrityl

DMT-Cl: 4,4'-dimethoxytrityl chloride

DTT: dithiothreitol

EDTA: ethylenediaminetetraacetic acid

PAGE: polyacrylamide gel electrophoresis

DCC: N,N'-dicyclohexylcarbodiimide

NHS: N-hydroxysuccinimide

FBS: fetal bovine serum

DPBS: dulbecco's phosphate-buffered saline

NBS: N-bromosuccinimide

$PPh_3$: triphenylphosphine

DBU: 1,8-diazabicyclo[5.4.0]undec-7-ene

TBAF: tetrabutylammonium fluoride

DIPEA: N,N-diisopropylethylamine

TEA: triethylamine

EtOAc: ethyl acetate

EEDQ: 2-ethoxy-1-ethoxycarbonyl-1,2-dihydroquinoline

DMF: dimethylformamide

TBDMS-Cl: tert-butyldimethylsilyl chloride

## 2. Materials

For organic synthesis, all reagents were purchased commercially and used without further treatment. Cathepsin B from human placenta, E-64 protease inhibitor and cell counting kit-8 were purchased from Sigma-Aldrich. Ultrapure deionized water was used in all experiments, excluding organic syntheses, and was obtained from a RiOs$^{TM}$ 3 water purification system.

## 3. Experimental section

**DNA synthesis**

All oligonucleotides used in this work were synthesized on an Applied Biosystems 394 DNA/RNA Synthesizer with standard phosphoramidite chemistry. Val-Ala-01 and Val-Ala-02 phosphoramidites were dissolved in anhydrous dichloromethane at 0.1 M concentration. Val-Ala-Coumarin phosphoramidite was dissolved in a mixture of anhydrous tetrahydrofuran and acetonitrile 1:1 v/v at 0.1 M concentration. Val-Ala-Chalcone phosphoramidite was dissolved in anhydrous dichloromethane at 0.05 M concentration. After DNA synthesis, the oligonucleotides were deprotected (Table S2). The deprotection solution was then removed at high vacuum, and the oligonucleotides were subjected to HPLC purification. After purification, oligonucleotides were dried. If necessary, 80% acetic acid aqueous solution was added to remove the 4,4'-dimethoxytrityl (DMT) group from oligonucleotides. After desalting by NAP column, the oligonucleotides were quantified by measuring their absorbance at 260 nm.

**Mass spectrometry**

After oligonucleotides synthesis, 20 μL desalted oligonucleotides (20 μM) were subjected to LC-MS analysis. For the oligonucleotides cleaved by cathepsin B in buffer A solution, HPLC was used to purify the resultant oligonucleotides, and the collected oligonucleotides were analyzed by LC-MS.

**Enzymatic cleavage**

Cathepsin B (2 U) was dissolved in 400 μL activation buffer (25 mM sodium acetate, 5 mM DTT, 1 mM EDTA, pH 5.0) to prepare the stock solution (5 U/mL). The oligonucleotides were diluted with buffer A (25 mM sodium acetate, 5 mM DTT, pH 5.0) or buffer B (25 mM sodium acetate, 5 mM DTT, 1 mM $MgCl_2$, pH 5.0) to the desired concentration. Then, oligonucleotides were incubated with cathepsin B at 37 °C for the desired times. When the incubation was complete, the mixture was subjected to HPLC, fluorescence or PAGE assays.

**Fluorescence assay**

Fluorescence spectra of oligonucleotides were measured in buffer A or B solutions at 37 °C. For FAM-labeled oligonucleotides, the excitation wavelength was 480 nm, and the emission wavelength was set between 495 nm and 700 nm with both slits set as 7 nm. For fluorescence spectra of ODN18, the excitation wavelength was 350 nm, and the emission wavelength was set between 380 nm and 650 nm with both slits set as 7 nm.

**PAGE assay**

12% Native or denaturing PAGE gels were used to analyze the cleavage of oligonucleotides by cathepsin B. The oligonucleotides were incubated with and without cathepsin B in buffer B at 37 °C for the desired times. Then, the mixture was subjected to a PAGE assay. For native PAGE gels, the gel was run at 4 °C for one hour and the power was set as 20 W. For denaturing PAGE gels, the gel was run at room temperature for one hour and the power was set as 20 W.

**Cell viability assay**

HepG2 cells were cultured in DMEM culture media supplemented with 10% FBS, 0.1 U/mL penicillin and 0.1 mg/mL streptomycin under 5% $CO_2$ atmosphere. For cell viability assay, about 10,000 cells were cultured in each well of a 96-well cell culture plate before the experiment. After 24 hours, the HepG2 cells were treated with chalcone or ODN1 in DMEM (10% FBS, 0.1 U/mL penicillin and 0.1 mg/mL streptomycin) cell culture medium for the desired times (96 hours for chalcone and 72 hours for ODN1). Then, 10 μL cell counting kit-8 were added to each well. Cell viability was analyzed by measuring absorbance at 450 nm.

## 4. Tables and figures

**Table S1**. Sequences of oligonucleotides

| Name | Sequence (5' to 3') |
|---|---|
| ODN1 | TTT TTT TT-Val-Ala-02-TTT TTT TTT TTT |
| ODN2 | FAM-TTT T-Val-Ala-02-TTT T-Dabcyl |
| ODN3 | FAM-CAC CGC TCT AAA TCT T-Val-Ala-02-TTC AGT CGC GCG GTG-Dabcyl |
| ODN4 | FAM-GAT ACT GCT T-Val-Ala-02-TT GAT ATG ACC |
| ODN5 | GGT CAT ATC AAA AGC AGT ATC-Dabcyl |
| ODN6 | FAM-CAC CGC T-Val-Ala-02-T GCG GTG-Dabcyl |
| ODN7 | CAC CGC TTT-Val-Ala-02-TTT GCG GTG-FAM |
| ODN8 | CAA TCT GCT T-Val-Ala-02-TTG ATA TGA CCC CGG TAT GTC GC-FAM |
| ODN9 | CAA TCT GCT TTT TTT T-Val-Ala-02-TTT TTT TTG ATA TGA CCC CGG TAT GTC GC-FAM |
| ODN10 | GCG ACA TAC CGG GGT CAT ATC AAA AGC AGA TTG |
| ODN11 | GCG ACA TAC CGG GGT CAT ATC AAT TTT AAG CAG ATT G |
| ODN12 | GCG ACA TAC CGG GGT CAT ATC AAT TTT TTT TAA GCA GAT TG |
| ODN13 | GCG ACA TAC CGG GGT CAT ATC AAT TTT TTT TTT TTA AGC AGA TTG |
| ODN14 | GCG ATC GAG GTA GGT CAT ATC AAA AGC AGT ATC |
| ODN15 | TTG ATA TGA CCT ACC TCG ATC GC |
| ODN16 | FAM-$U_{OMe}U_{OMe}U_{OMe}$-Val-Ala-02-$U_{OMe}U_{OMe}U_{OMe}$-Dabcyl |
| ODN17 | FAM-$U_{OMe}U_{OMe}U_{OMe}U_{OMe}U_{OMe}U_{OMe}U_{OMe}U_{OMe}$-Dabcyl |
| ODN18 | Val-Ala-Coumarin-TTT TTT TTT TTT TTT |
| ODN19 | TTT TTT TT-Val-Ala-Chalcone-TTT TTT TT |

Note: $U_{OMe}$ indicates 2'-OMe-U.

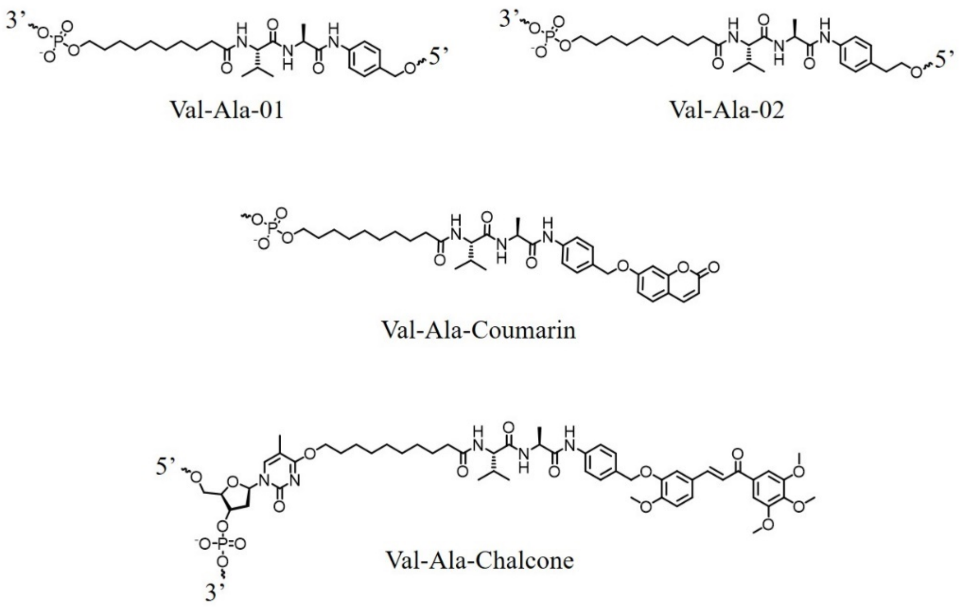


**Figure S1**. Chemical structures of Val-Ala-01, Val-Ala-02, Val-Ala-Coumarin and Val-Ala-Chalcone modifiers in oligonucleotides.

**Table S2**. Oligonucleotide deprotection methods

| Name | Deprotection method |
|---|---|
| ODN1 | Ammonium hydroxide/40% aqueous methylamine, 1:1, 55 °C, 0.5 h |
| ODN2 | Ammonium hydroxide, 55 °C, 17 h |
| ODN3 | Ammonium hydroxide, 55 °C, 17 h |
| ODN4 | Ammonium hydroxide, 55 °C, 17 h |
| ODN5 | Ammonium hydroxide, 55 °C, 17 h |
| ODN6 | Ammonium hydroxide, 55 °C, 17 h |
| ODN7 | Ammonium hydroxide, 55 °C, 17 h |
| ODN8 | Ammonium hydroxide, 55 °C, 17 h |
| ODN9 | Ammonium hydroxide, 55 °C, 17 h |
| ODN16 | Ammonium hydroxide, 55 °C, 17 h |
| ODN17 | Ammonium hydroxide, 55 °C, 17 h |
| ODN18[a] | 50 mM $K_2CO_3$ in methanol, room temperature, 3 h |
| ODN19[a] | 50 mM $K_2CO_3$ in methanol, room temperature, 4 h |

Note: [a] After deprotection by 50 mM $K_2CO_3$ in methanol (2 mL), 12 μL of acetic acid were added to neutralize the solution. Then, the solvent was removed under high vacuum and the oligonucleotides were subjected to HPLC purification.

**Table S3**. Calculated and observed mass of oligonucleotides

| Name | Calculated mass (Da) | Observed mass (Da) |
|---|---|---|
| ODN1 | 6560.5 | 6563 (Figure S21) |
| ODN2 | 3910.2 | 3912 (Figure S22) |
| ODN3 | 10976.7 | 10979 (Figure S23) |
| ODN4 | 7487.4 | 7489 (Figure S24) |
| ODN5 | 6900.7 | 6901 (Figure S25) |
| ODN6 | 5794.4 | 5796 (Figure S26) |
| ODN7 | 6580.7 | 6582 (Figure S27) |
| ODN8 | 11207.7 | 11209 (Figure S28) |
| ODN9 | 14858.1 | 14860 (Figure S29) |
| ODN10 | 10195.7 | 10197 |
| ODN11 | 11412.5 | 11414 |
| ODN12 | 12629.2 | 12630 |
| ODN13 | 13846 | 13847 |
| ODN14 | 10210.7 | 10210 |
| ODN15 | 6974.6 | 6975 |
| ODN16 | 3397.2 | 3399 (Figure S30) |
| ODN17 | 3498.8 | 3500 (Figure S30) |
| ODN18 | 5169.7 | 5171 (Figure S31) |
| ODN19 | 5880.3 | 5882 (Figure S32) |

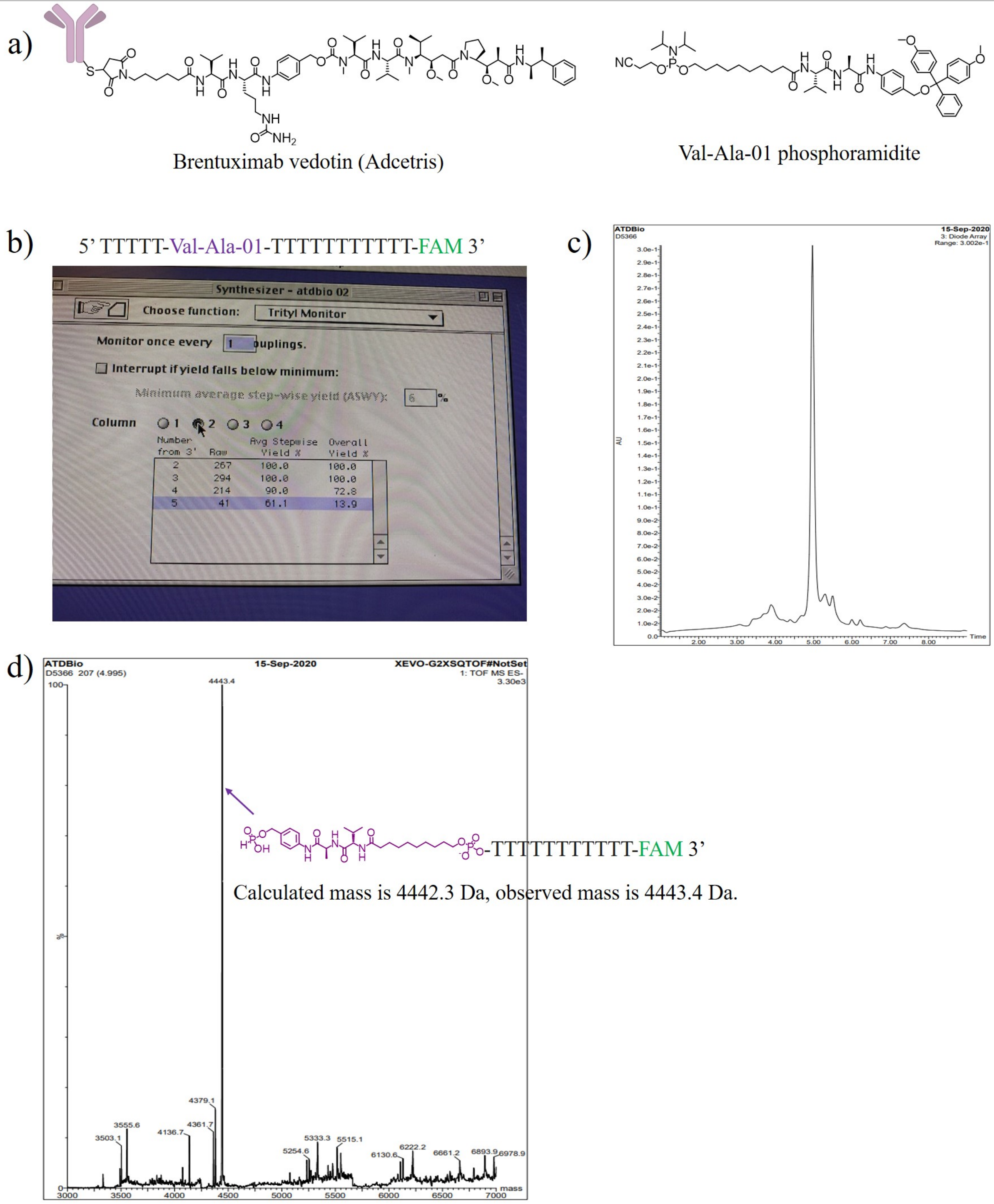


**Figure S2**. Synthesis of Val-Ala-01-containing oligonucleotide. (**a**) Chemical structures of brentuximab vedotin (Adcetris) and Val-Ala-01 phosphoramidite. (**b**) The designed sequence of Val-Ala-01-containing oligonucleotide and the stepwise yield record on DNA synthesizer. The trityl signal number 3 indicates the coupling yield of Val-Ala-01. The trityl signal number 4 indicates the coupling yield of thymidine after Val-Ala-01. The poor coupling yield of thymidine after Val-Ala-01 suggests that Val-Ala-01 is unstable on the DNA synthesizer (trityl monitoring started at the 10th thymidine from the 3'-end of the oligonucleotide). (**c**) HPLC chromatogram of the oligonucleotide after deprotection. (**d**) Mass

spectrum of the oligonucleotide after deprotection. The observed molecular weight is 4443.4 Da, which is consistent with the molecular weight of phosphite-Val-Ala-01-TTTTTTTTTT-FAM (4442.3 Da), demonstrating the degradation of Val-Ala-01 during DNA synthesis.

**Table S4**. Conditions for enzymatic cleavage of ODN1 by cathepsin B in buffer A (25 mM sodium acetate, 5 mM DTT, pH 5.0).

| | Entry 1 | Entry 2 | Entry 3 | Entry 4 |
|---|---|---|---|---|
| ODN1 | 5 μM | 5 μM | 5 μM | 5 μM |
| Cathepsin B | No | 0.2 U/mL | 0.2 U/mL | 0.2 U/mL |
| Incubation time | 1 hour | 1 hour | 6 hours | 22 hours |

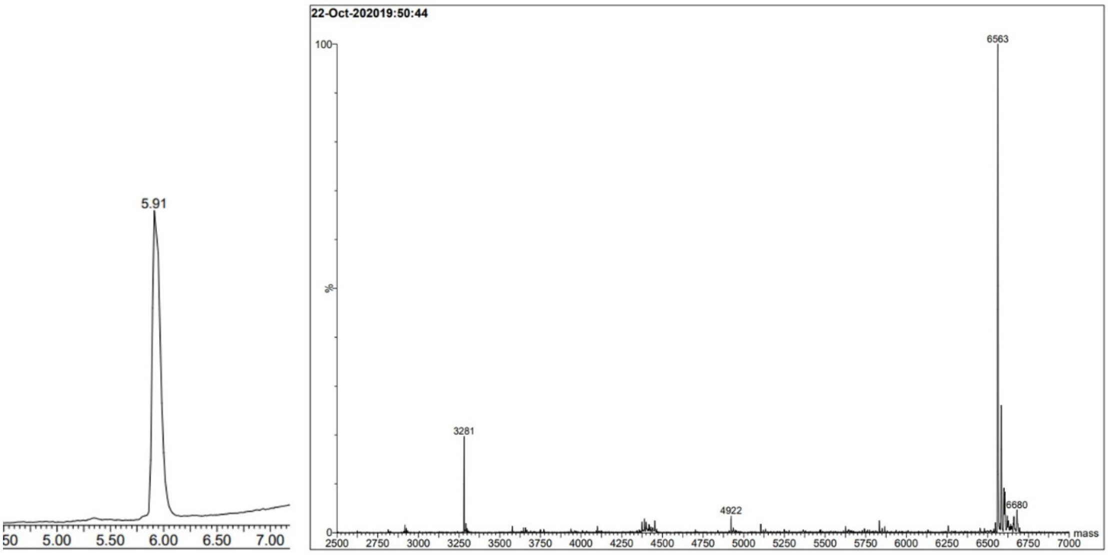


**Figure S3.** HPLC chromatogram (left) and mass spectrum (right) of ODN1 (5 μM) after incubation in buffer A (without cathepsin B) for one hour at 37 °C (entry 1 in Table S4). The calculated molecular weight is 6560.5 Da, and the observed molecular weight is 6563 Da, indicating that ODN1 is stable in buffer A solution. The mass peak of 3281 Da is due to the half molecular weight of ODN1.

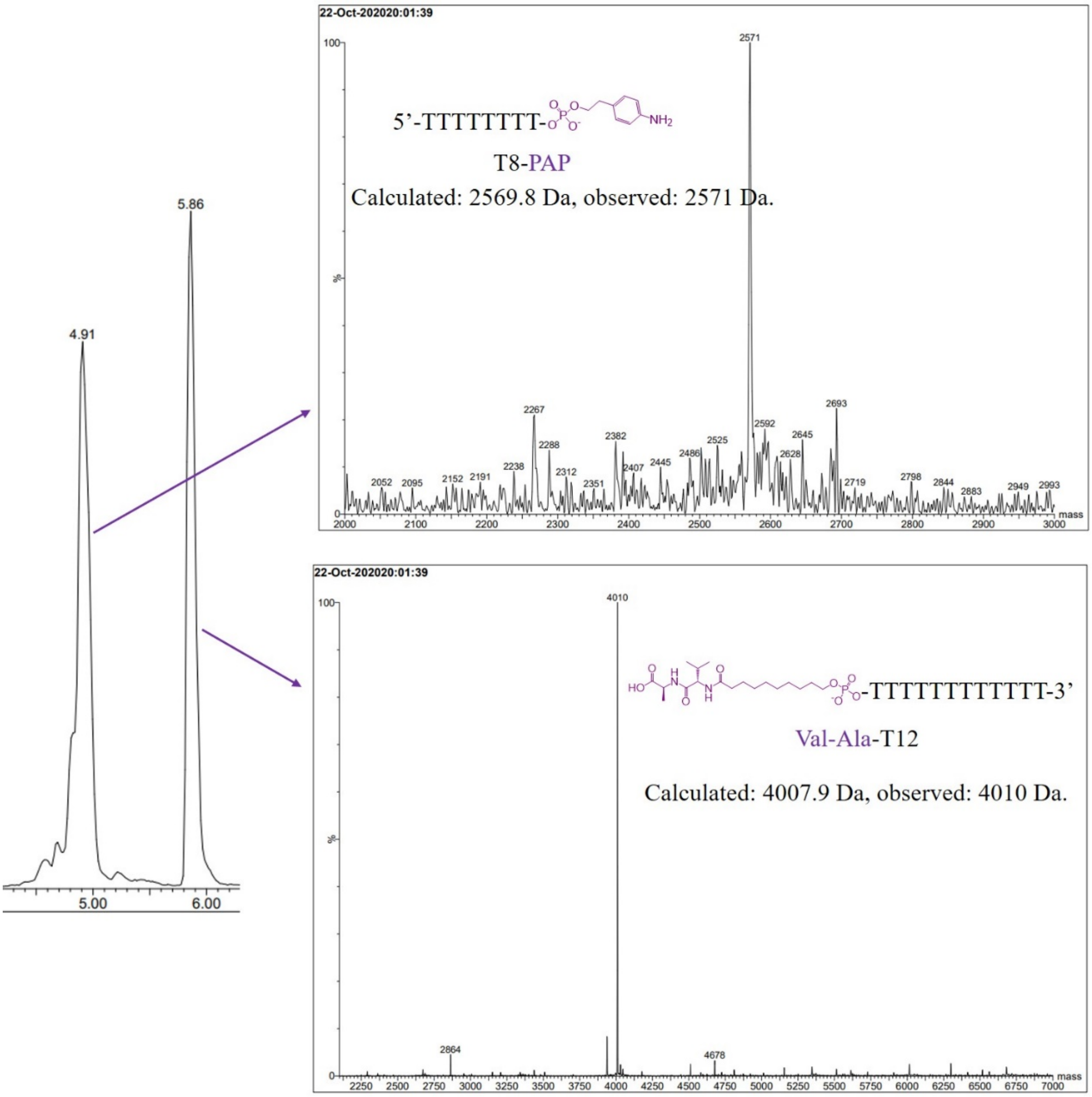


**Figure S4.** HPLC chromatogram (left) and mass spectrum (right) of ODN1 (5 μM) after incubation with 0.2 U/mL cathepsin B in buffer A for one hour at 37 °C (entry 2 in Table S4). The molecular weight of oligo peaks at 4.91 min and 5.86 min is 2571 Da and 4010 Da, consistent with the molecular weight of T8-PAP (2569.8 Da) and Val-Ala-T12 (4007.9 Da), respectively. These results demonstrate that cathepsin B had converted ODN1 into T8-PAP and Val-Ala-T12 in buffer A after one hour of incubation at 37 °C.

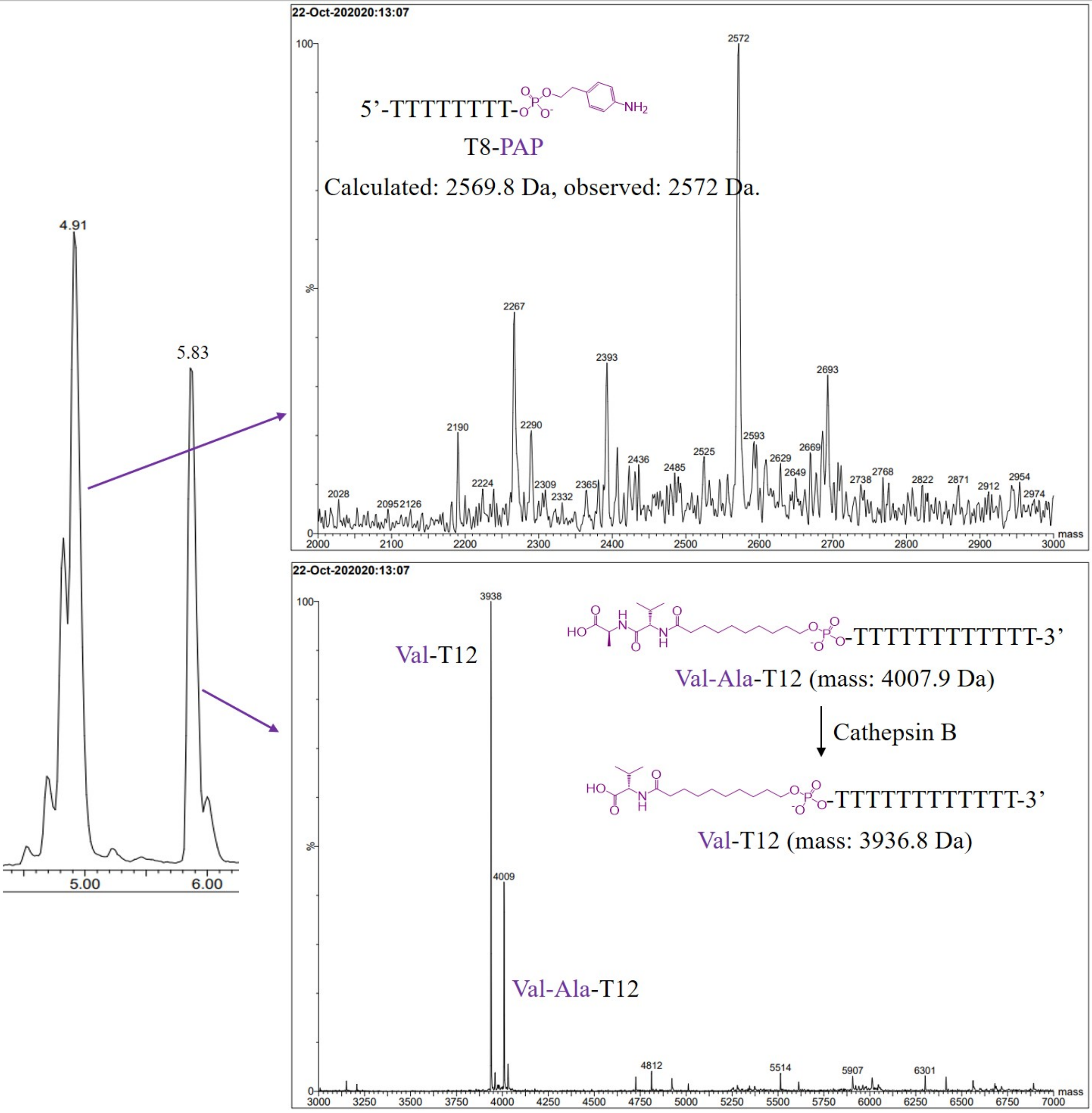


**Figure S5.** HPLC chromatogram (left) and mass spectrum (right) of ODN1 (5 μM) after incubation with 0.2 U/mL cathepsin B in buffer A for six hours at 37 °C (entry 3 in Table S4). The molecular weight of oligo peak at 4.91 min is 2572 Da, which is consistent with the molecular weight of T8-PAP (2569.8 Da). The molecular weight of oligo peak at 5.83 min is 4009 Da and 3938 Da, consistent with the molecular weight of Val-Ala-T12 (4007.9 Da) and Val-T12 (3936.8 Da), respectively. These results demonstrated that cathepsin B further cleaved Val-Ala-T12 to Val-T12 in buffer A after six hours of incubation at 37 °C.

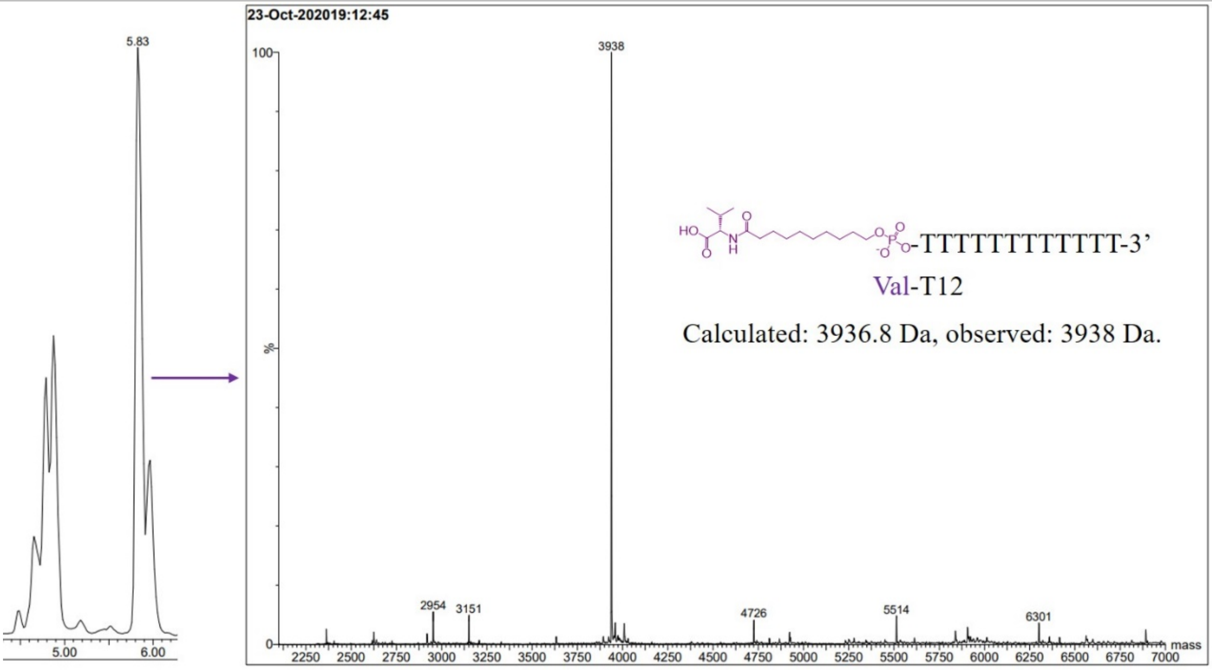


**Figure S6**. HPLC chromatogram (left) and mass spectrum (right) of ODN1 (5 μM) after incubation with 0.2 U/mL cathepsin B in buffer A for 22 hours at 37 °C (entry 4 in Table S4). The molecular weight of oligo peak at 5.83 min is 3938 Da, which is consistent with the molecular weight of Val-T12 (3936.8 Da), suggesting that cathepsin B had completely converted Val-Ala-T12 to Val-T12 in buffer A after twenty-two hours of incubation at 37 °C.

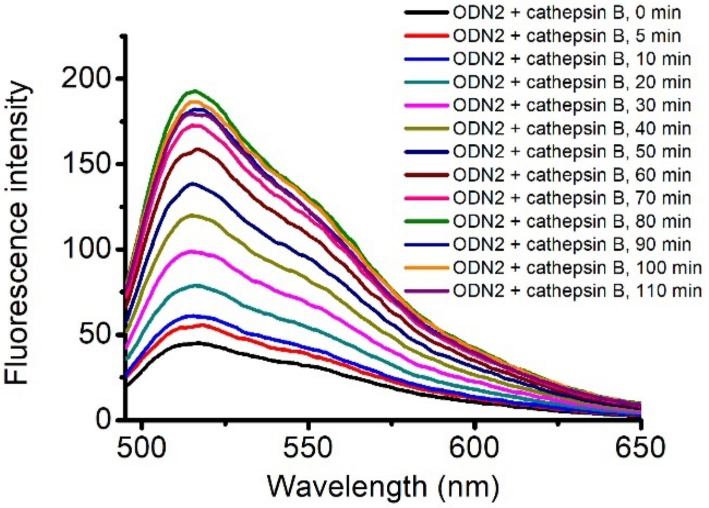


**Figure S7**. Fluorescence spectra of 2 μM ODN2 after incubation with 0.2 U/mL cathepsin B in buffer A as a function of time.

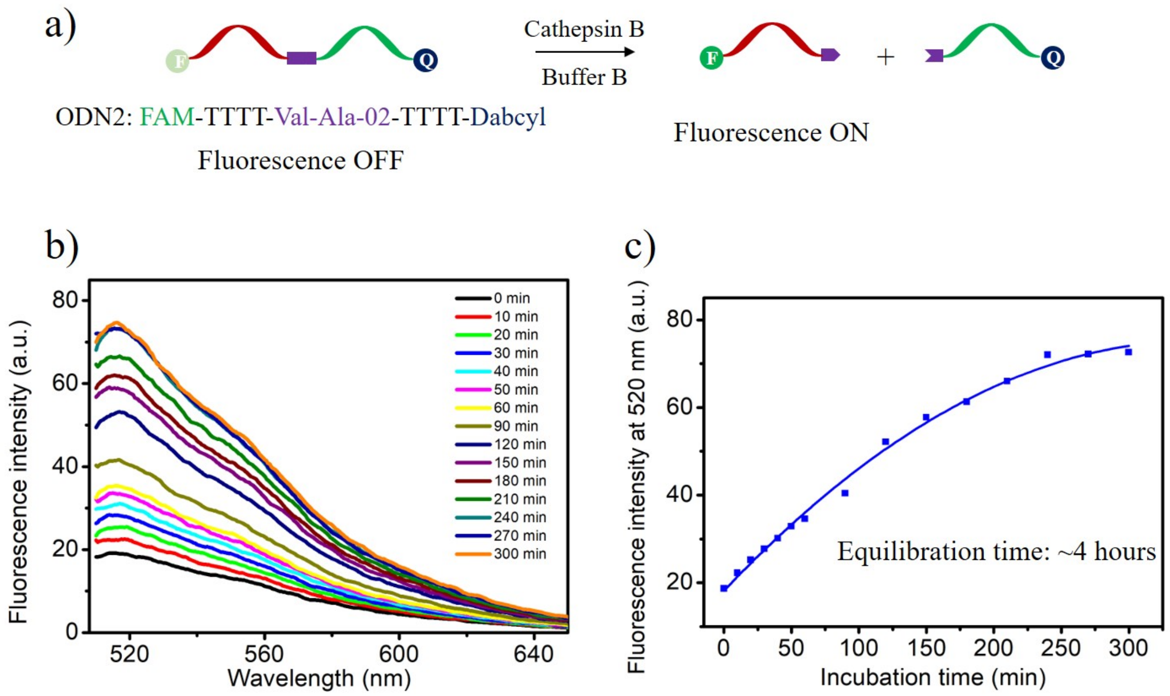


**Figure S8.** Cathepsin B-mediated cleavage of ODN2 in buffer B at 37 °C. (**a**) Schematic illustration of cathepsin B-responsive cleavage and fluorescence recovery of ODN2. (**b**) Fluorescence spectra of ODN2 (1 μM) incubated with 0.1 U/mL cathepsin B in buffer B at 37 °C for various times. (**c**) Fluorescence intensity of ODN2 (1 μM) at 520 nm after incubation with 0.1 U/mL cathepsin B in buffer B at 37 °C for various times.

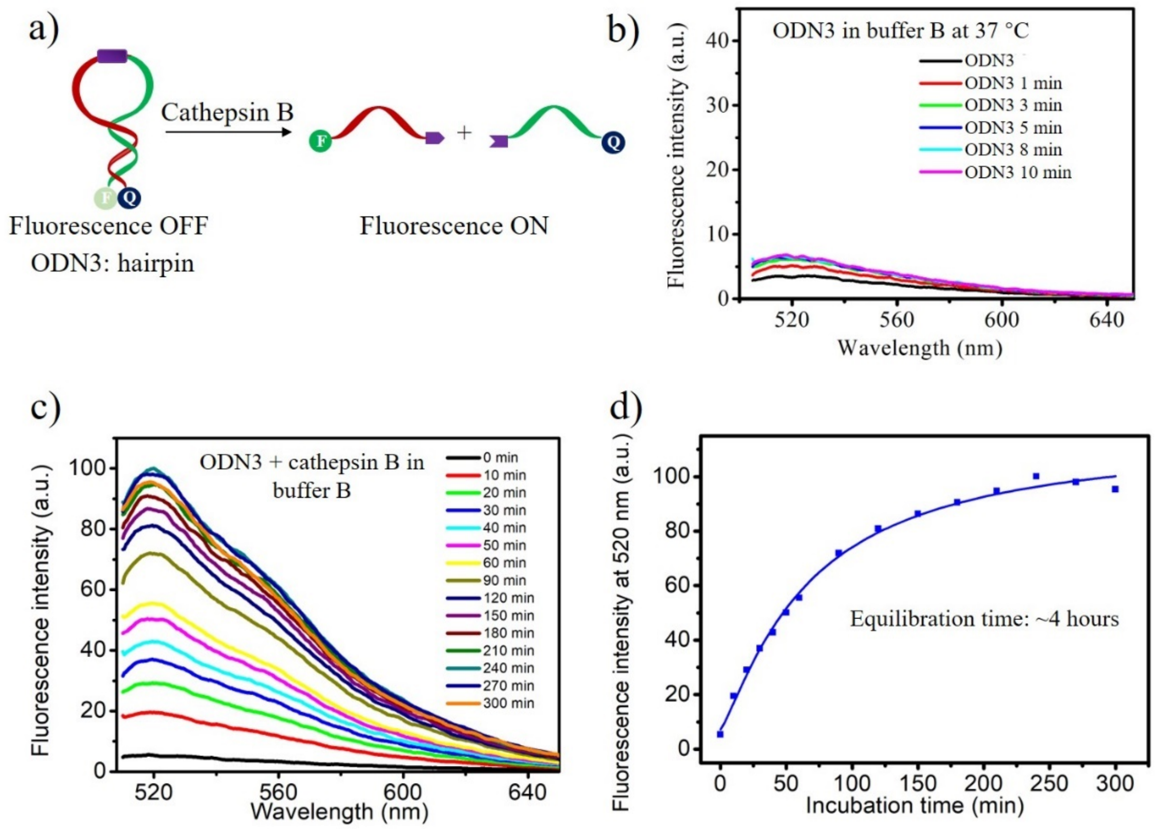


**Figure S9.** Cathepsin B-mediated cleavage of ODN3 in buffer B at 37 °C. (**a**) Schematic illustration of cathepsin B-responsive cleavage and fluorescence recovery of ODN3. (**b**) Fluorescence spectra of ODN3 (1 μM) in buffer B at 37 °C for various times. No obvious change of fluorescence emission at 520 nm was observed, demonstrating that the hairpin structure is stable in buffer B at 37 °C. (**c**) Fluorescence spectra of ODN3 (1 μM) after incubation with 0.1 U/mL cathepsin B in buffer B at 37 °C for various times. (**d**) Fluorescence intensity of ODN3 (1 μM) at 520 nm after incubation with 0.1 U/mL cathepsin B in buffer B at 37 °C for various times.

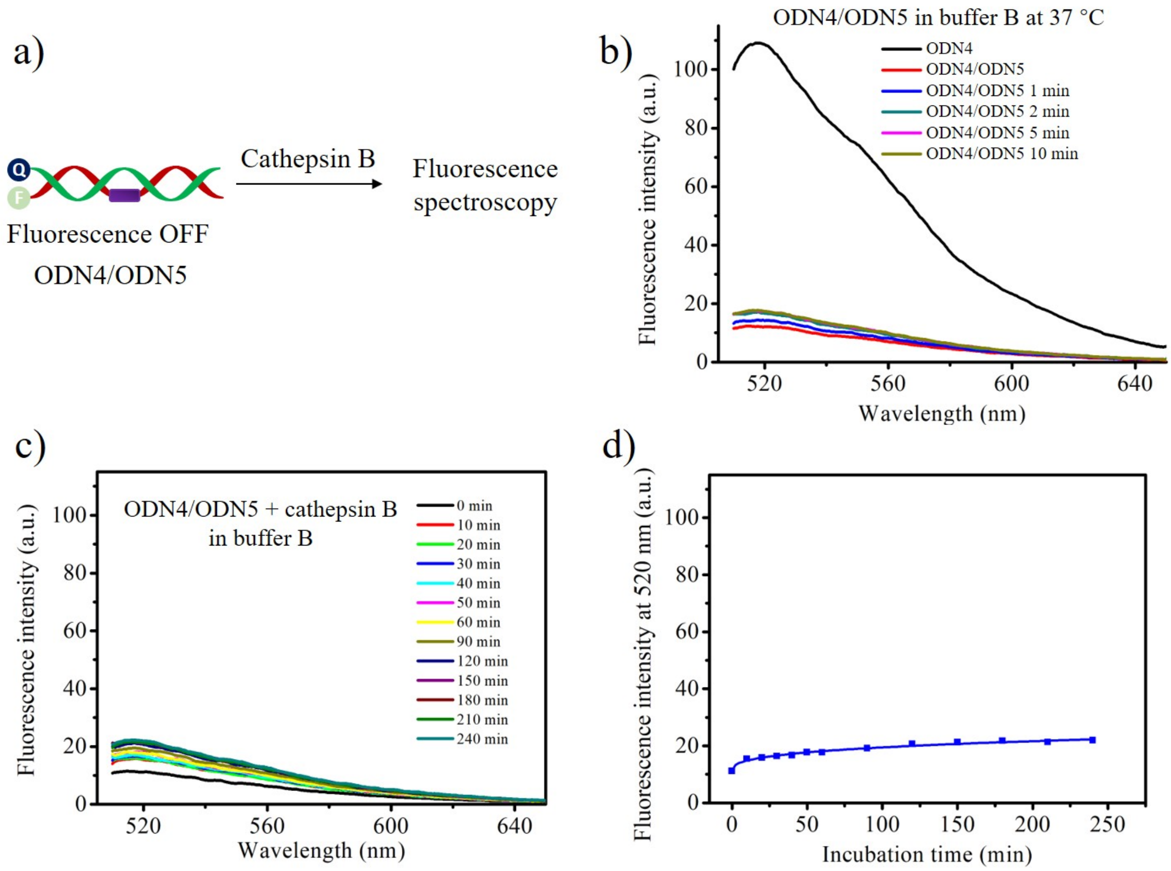


**Figure S10.** Cathepsin B-mediated cleavage of ODN4/ODN5 in buffer B at 37 °C. (**a**) Schematic illustration of cathepsin B-responsive cleavage of ODN4/ODN5. (**b**) Characterization of the formation of ODN4/ODN5 and its stability in buffer B at 37 °C. FAM-labeled ODN4 (1 μM) shows bright fluorescence emission at 520 nm (black line). The addition of Dabcyl-labeled ODN5 (1 μM) quenched the fluorescence of ODN4 (red line), indicating the formation of the ODN4/ODN5 double-stranded structure. No obvious change of fluorescence emission of ODN4/ODN5 at 520 nm was observed after ten minutes at 37 °C, demonstrating that ODN4/ODN5 is a stable double-stranded structure in buffer B at 37 °C. (**c**) Fluorescence spectra of ODN4/ODN5 (1 μM) after incubation with 0.1 U/mL cathepsin B in buffer B at 37 °C for various times. (**d**) Fluorescence intensity of ODN4/ODN5 (1 μM) at 520 nm after incubation with 0.1 U/mL cathepsin B in buffer B at 37 °C for various times.

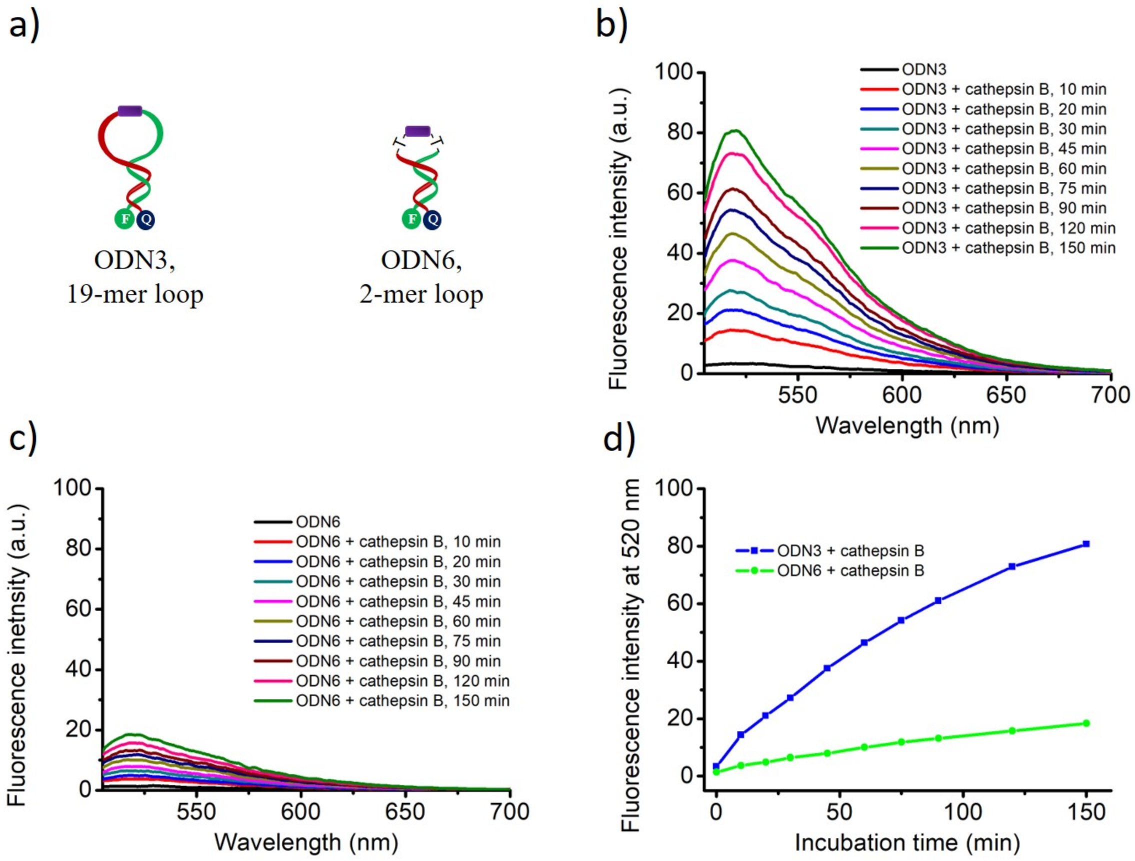


**Figure S11.** Cathepsin B-mediated cleavage of ODN3 and ODN6 in buffer B solution. (**a**) Schematic illustration of ODN3 and ODN6 with different lengths of loop structure. (**b**) Fluorescence spectra of ODN3 (1 μM) after incubation with 0.1 U/mL cathepsin B in buffer B at 37 °C for various times. (**c**) Fluorescence spectra of ODN6 (1 μM) after incubation with 0.1 U/mL cathepsin B in buffer B at 37 °C for various times. (**d**) Fluorescence intensity of 1 μM ODN3 and ODN6 at 520 nm after incubation with 0.1 U/mL cathepsin B in buffer B at 37 °C for various times.

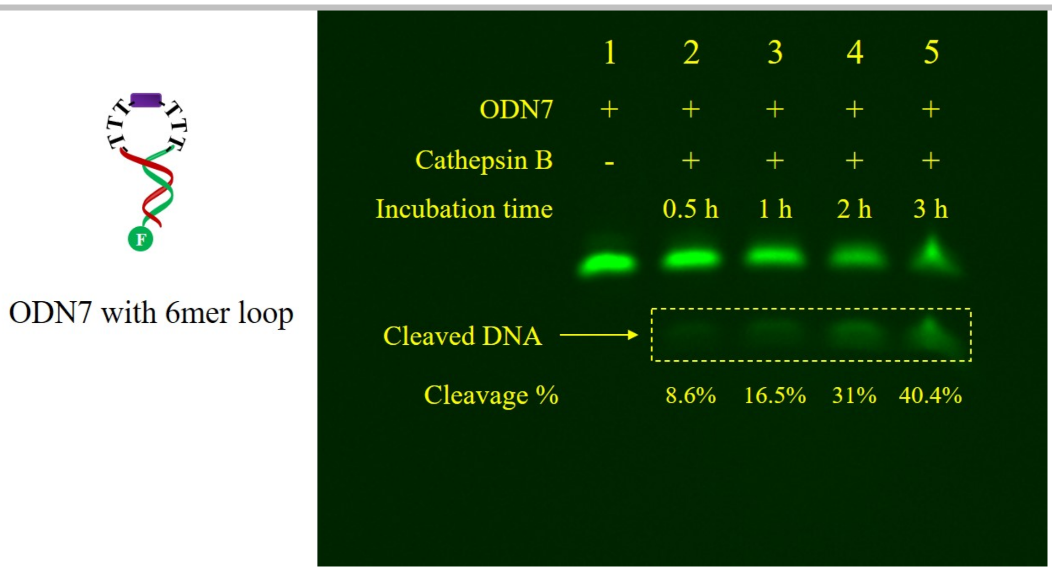


**Figure S12.** 12% Denaturing PAGE gel electrophoresis assay of cathepsin B-mediated cleavage of Val-Ala-02 in hairpin structure with 6mer loop size (ODN7). 2 μM ODN7 was incubated with 0.5 U/mL cathepsin B in buffer B solution at 37 °C for various times. After incubation, the samples were subjected to denaturing PAGE gel electrophoresis assay. The gel was run at room temperature for one hour. The power was set as 20 W.

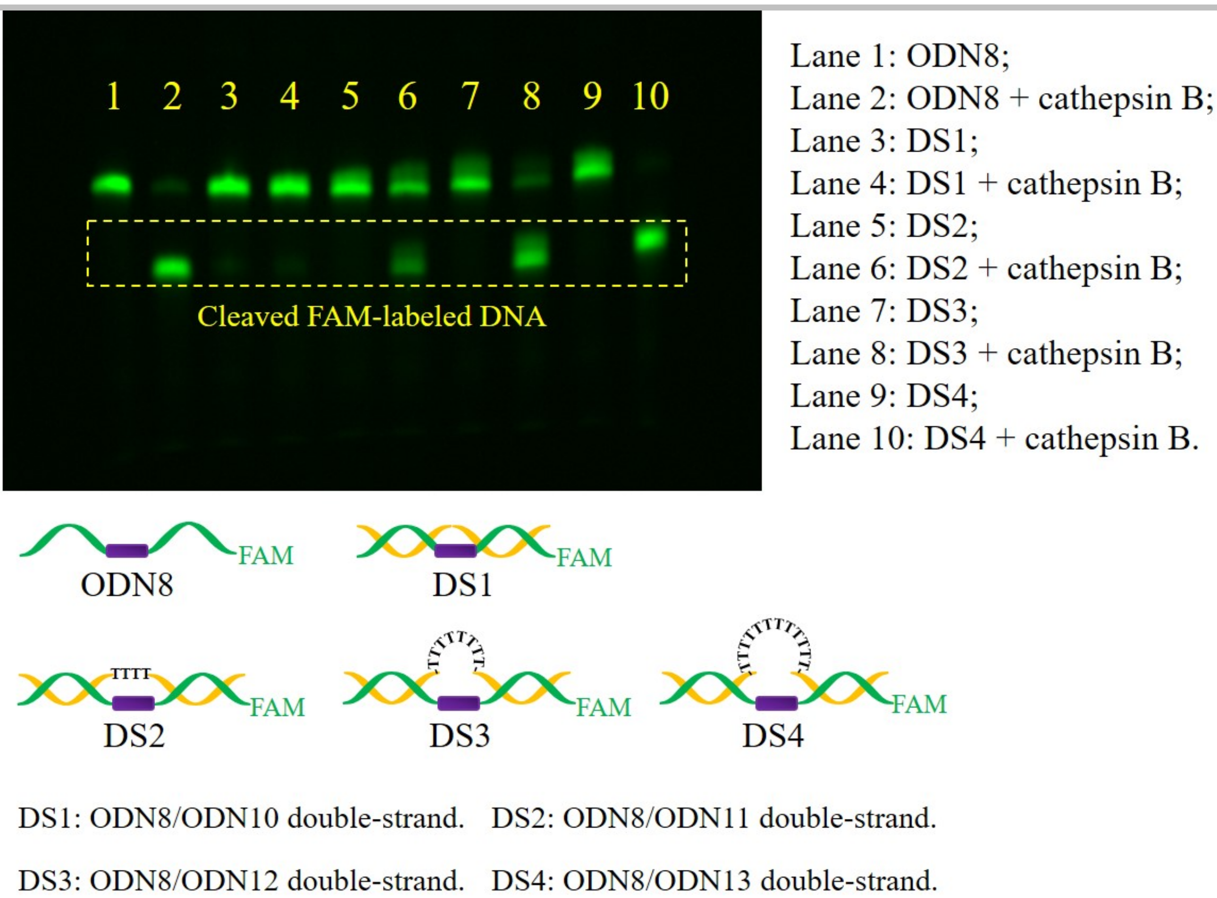


**Figure S13.** 12% Denaturing PAGE gel electrophoresis assay of cathepsin B-mediated cleavage of Val-Ala-02 in double-stranded oligonucleotides. 2 μM ODN8 hybridized with 3 μM cDNA (ODN10, ODN11, ODN12 or ODN13) in buffer B solution. Then, 0.5 U/mL cathepsin B were added. The mixture was incubated at 37 °C for one hour. After incubation, the samples were subjected to denaturing PAGE gel electrophoresis assay. The gel was run at room temperature for one hour. The power was set as 20 W.

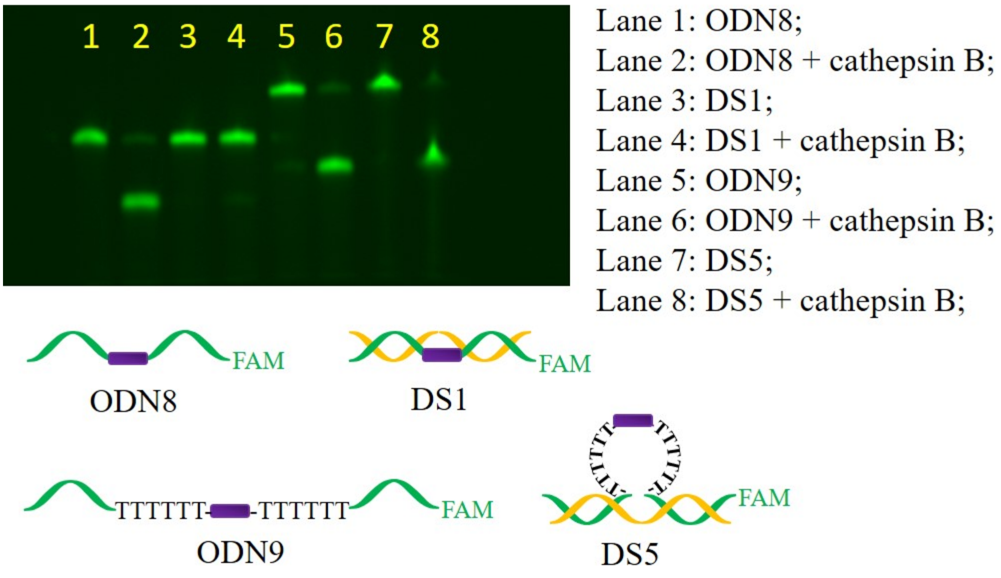


DS1: ODN8/ODN10 double-strand. DS5: ODN9/ODN10 double-strand.

**Figure S14.** 12% Denaturing PAGE gel electrophoresis assay of cathepsin B-mediated cleavage of Val-Ala-02 in double-stranded oligonucleotides. 2 μM ODN8 and ODN9 hybridized with 3 μM ODN10 in buffer B solution, respectively. Then, 0.5 U/mL cathepsin B were added. The mixture was incubated at 37 °C for one hour. After incubation, the samples were subjected to denaturing PAGE gel electrophoresis assay. The gel was run at room temperature for one hour. The power was set as 20 W.

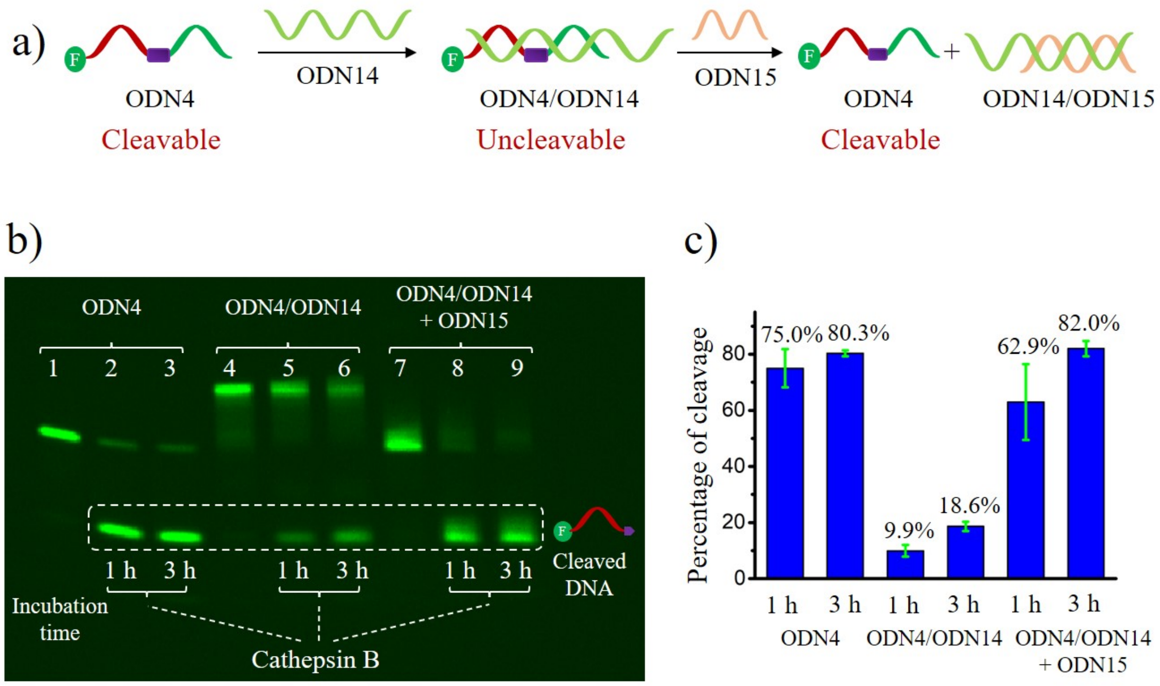


**Figure S15**. Toehold-mediated strand displacement controls the enzymatic cleavage of Val-Ala-02 in ODN4. (**a**) Displacement strand (ODN15) hybridized with ODN14 to release enzyme-cleavable ODN4 from enzyme-uncleavable ODN4/ODN14. (**b**) 12% Native PAGE gel electrophoresis assay of controllable cleavage of Val-Ala-02 in ODN4. Lane 1: ODN4. Lane 2: ODN4 + cathepsin B, 1 h incubation. Lane 3: ODN4 + cathepsin B, 3 h incubation. Lane 4: ODN4/ODN14. Lane 5: ODN4/ODN14 + cathepsin B, 1 h incubation. Lane 6: ODN4/ODN14 + cathepsin B, 3 h incubation. Lane 7: ODN4/ODN14 + ODN15. Lane 8: ODN4/ODN14 + ODN15 + cathepsin B, 1 h incubation. Lane 9: ODN4/ODN14 + ODN15 + cathepsin B, 3 h incubation. (**c**) The percentage cleavage of Val-Ala-02 in ODN4, ODN4/ODN14 and ODN4/ODN14 + ODN15 groups. ODN4 (2 μM), ODN4 (2 μM)/ODN14 (2 μM) and ODN4 (2 μM)/ODN14 (2 μM) + ODN15 (4 μM) groups were incubated with 0.5 U/mL cathepsin B in buffer B for one or three hours at 37 °C, then, subjected to a 12% native PAGE gel electrophoresis assay, and the percentage cleavage was analyzed. The gel was run at 4 °C for one hour. The power was set as 20 W. The error bars indicate the mean ± SD values; *n* = 3.

ODN16: 5' FAM-$U_{OMe}U_{OMe}U_{OMe}$-Val-Ala-02-$U_{OMe}U_{OMe}U_{OMe}$-Dabcyl 3'

ODN17: 5' FAM-$U_{OMe}U_{OMe}U_{OMe}U_{OMe}U_{OMe}U_{OMe}U_{OMe}U_{OMe}$-Dabcyl 3'

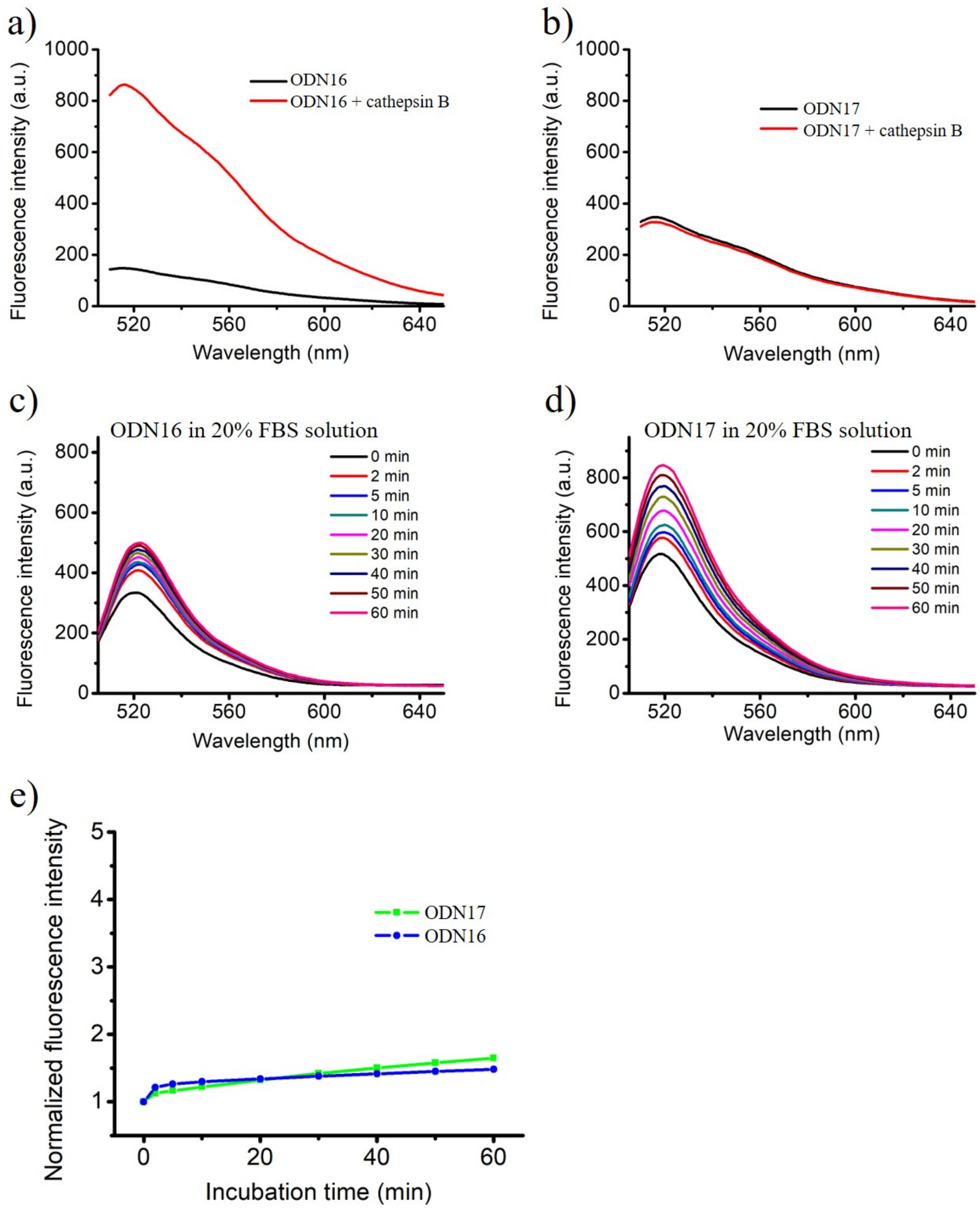


**Figure S16**. Stability analysis of Val-Ala-02-incorporated oligonucleotide in 20% FBS solution. (**a**) Fluorescence spectra of 5 μM ODN16 treated with or without 0.2 U/mL cathepsin B in buffer A at 37 °C for 16 hours. (**b**) Fluorescence spectra of 5 μM ODN17 treated with or without 0.2 U/mL cathepsin B in buffer A at 37 °C for 16 hours. (**c**) Fluorescence spectra of 5 μM ODN16 incubated with 20% FBS in DPBS solution at 37 °C for various times. (**d**) Fluorescence spectra of 5 μM ODN17 incubated with 20% FBS in DPBS solution at 37 °C for various times. (**e**) Normalized fluorescence intensity of ODN16 and ODN17 at 520 nm after incubation with 20% FBS in DPBS solution at 37 °C for various times.

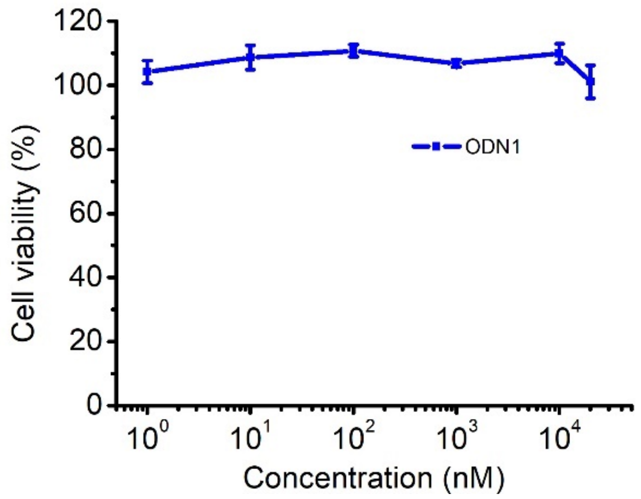


**Figure S17**. Cell viability assay of HepG2 cells treated with ODN1. No obvious decline of cell viability of HepG2 cells was observed indicating that ODN1 shows negligible cytotoxicity to HepG2 cells after 72 hours of incubation, even at concentrations of ODN1 up to 20 μM.

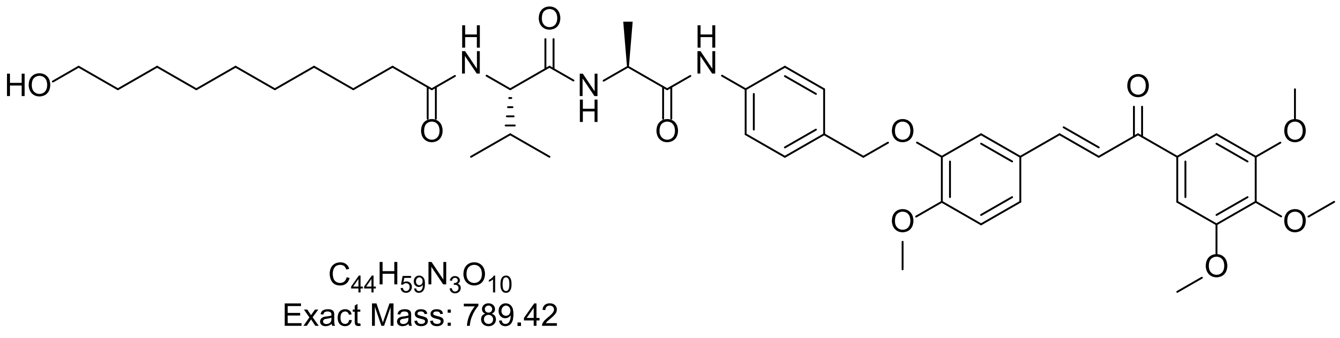


**Figure S18**. Chemical structure of HDA-Val-Ala-PAB-Chalcone

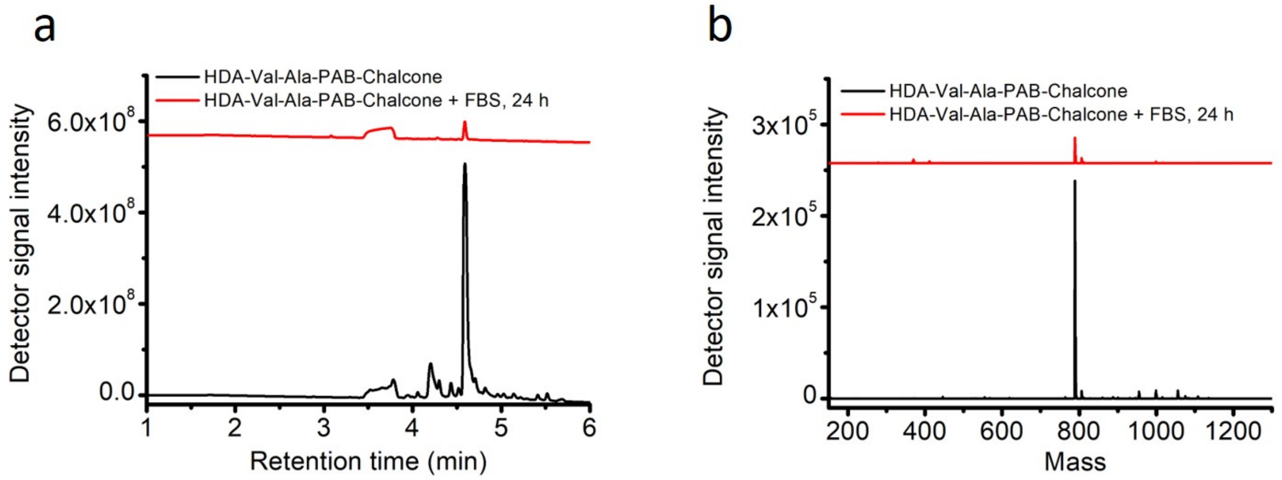


**Figure S19**. Stability assay of HDA-Val-Ala-PAB-Chalcone (Figure S18) in 95% FBS solution. (**a**) HPLC chromatograms. Black line, 5 mM HDA-Val-Ala-PAB-Chalcone. Red line, 0.2 mM HDA-Val-Ala-PAB-Chalcone after incubation with 95% FBS at 37 °C for 24 hours. (**b**) Mass spectra. Black line, 5 mM HDA-Val-Ala-PAB-Chalcone. Red line: 0.2 mM HDA-Val-Ala-PAB-Chalcone after incubation with 95% FBS at 37 °C for 24 hours. 25 μL HDA-Val-Ala-PAB-Chalcone in DMSO (4 mM) mixed with 475 μL FBS and incubated at 37 °C for 24 hours. Then, 500 μL chloroform was added, and the mixture was vortex for 20 seconds. The organic layer was collected and analyzed by mass spectrum. The calculated molecular weight of HDA-Val-Ala-PAB-Chalcone is 789.42 g/mol, and observed molecular weight is 789 g/mol, demonstrating HDA-Val-Ala-PAB-Chalcone is stable in 95% FBS solution, even at an incubation time up to 24 hours.

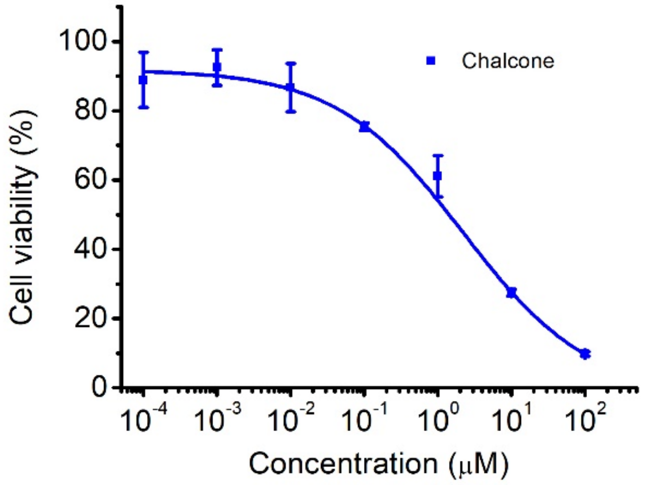


**Figure S20**. Cell viability assay of HepG2 cells treated with chalcone for 96 hours. The $IC_{50}$ value of chalcone to HepG2 cells is 3.6 μM.

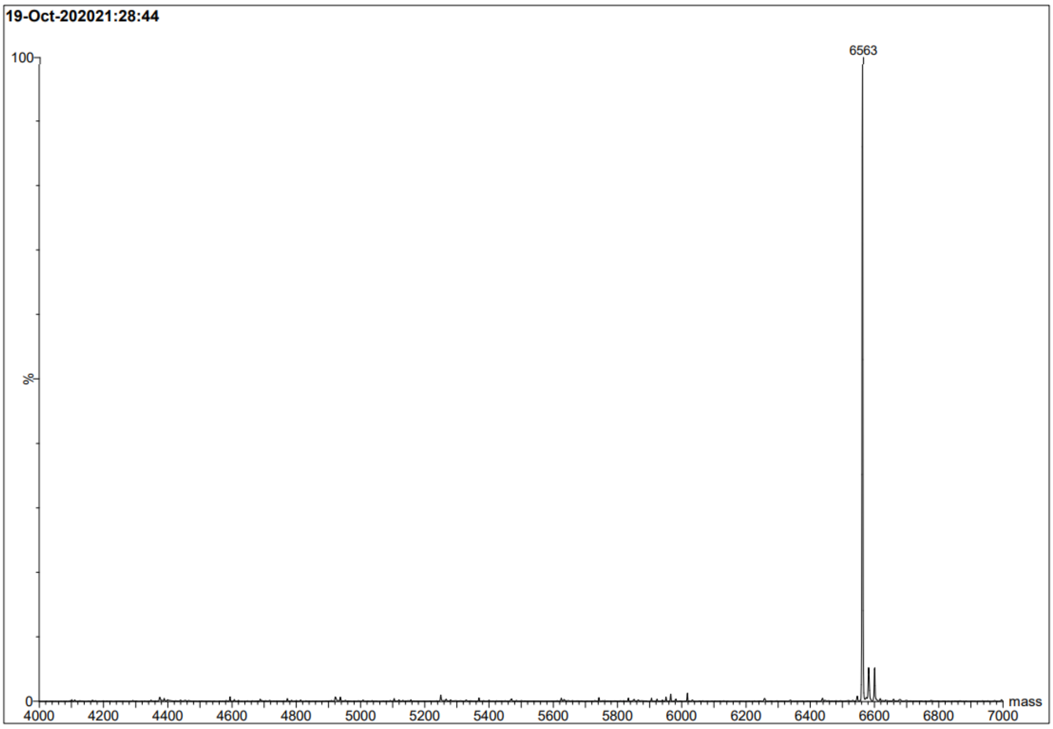


**Figure S21**. Mass spectrum of ODN1. The calculated molecular weight is 6560.5 Da, and the observed molecular weight is 6563 Da.

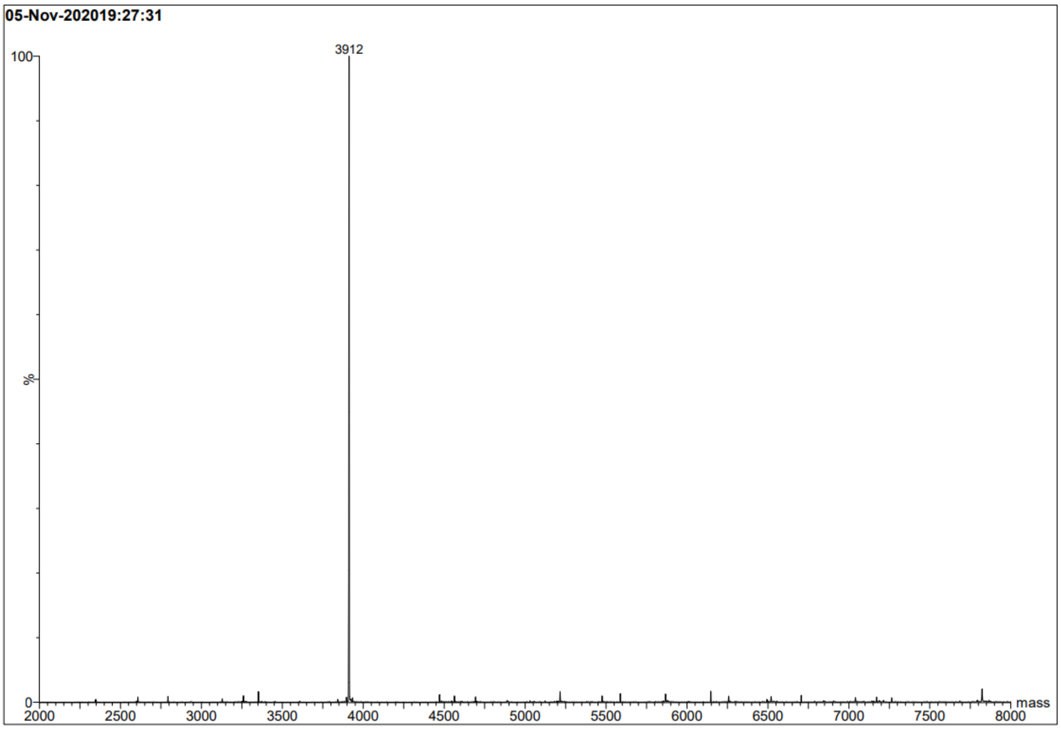


**Figure S22**. Mass spectrum of ODN2. The calculated molecular weight is 3910.2 Da, and the observed molecular weight is 3912 Da.

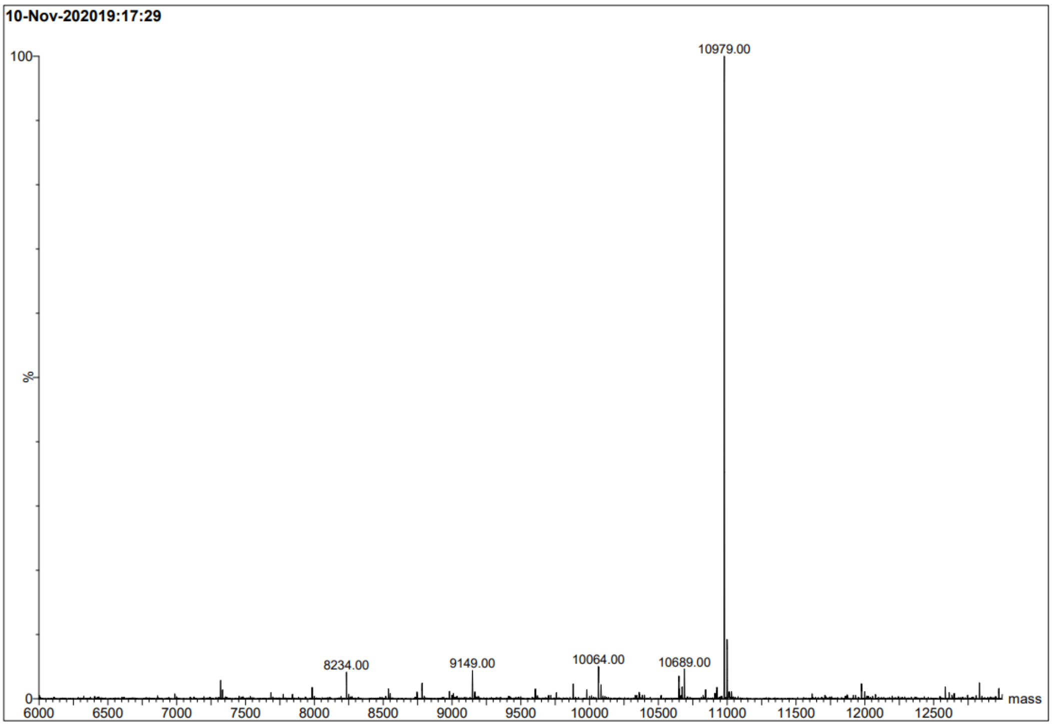


**Figure S23**. Mass spectrum of ODN3. The calculated molecular weight is 10976.7 Da, and the observed molecular weight is 10979 Da.

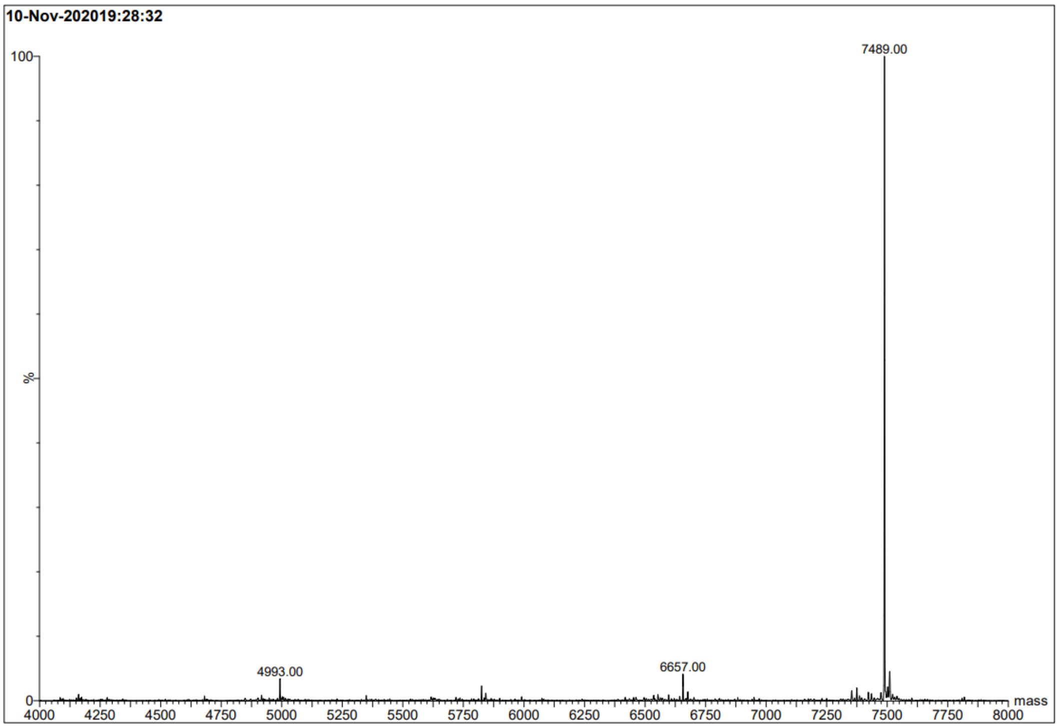


**Figure S24**. Mass spectrum of ODN4. The calculated molecular weight is 7487.4 Da, and the observed molecular weight is 7489.0 Da.

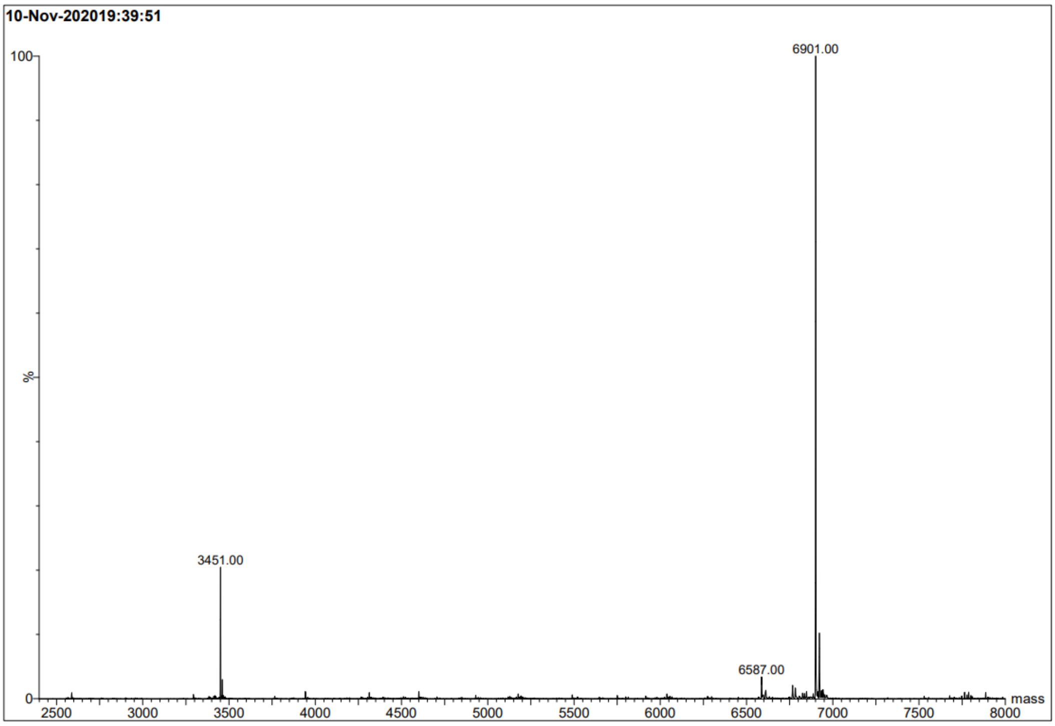


**Figure S25** Mass spectrum of ODN5. The calculated molecular weight is 6900.7 Da, and the observed molecular weight is 6901.0 Da. The mass peak of 3451.0 Da is due to the half molecular weight of ODN5.

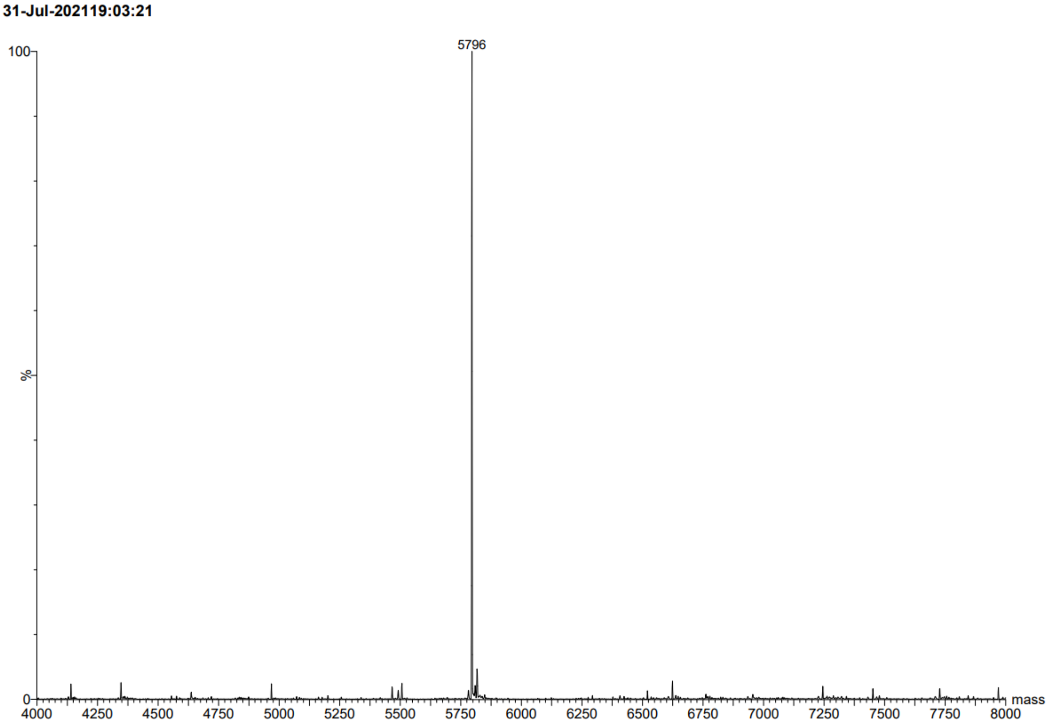


**Figure S26.** Mass spectrum of ODN6. The calculated molecular weight is 5794.4 Da, and the observed molecular weight is 5796 Da.

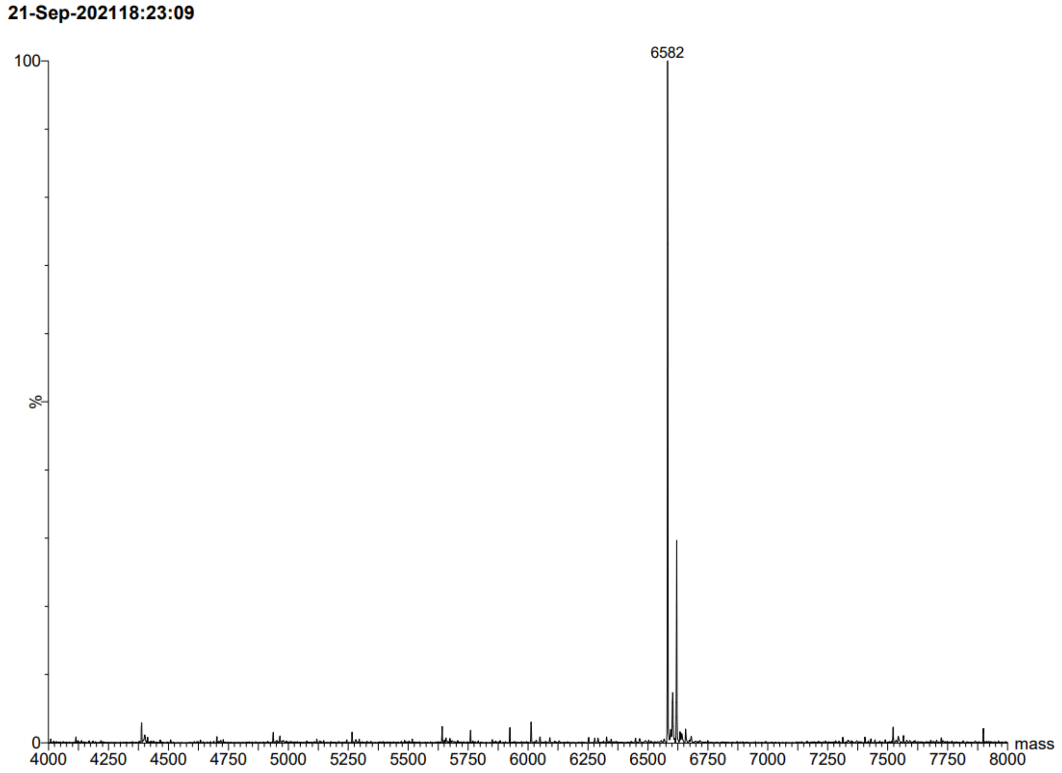


**Figure S27.** Mass spectrum of ODN7. The calculated molecular weight is 6580.7 Da, and the observed molecular weight is 6582 Da.

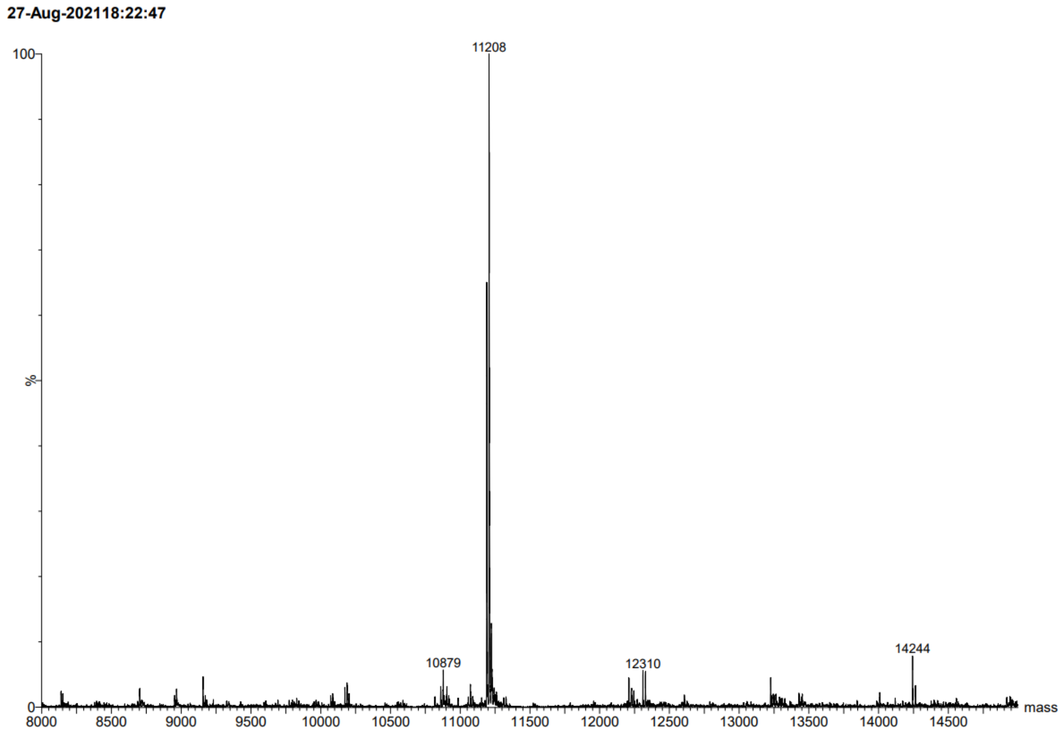


**Figure S28.** Mass spectrum of ODN8. The calculated molecular weight is 11207.7 Da, and the observed molecular weight is 11208 Da.

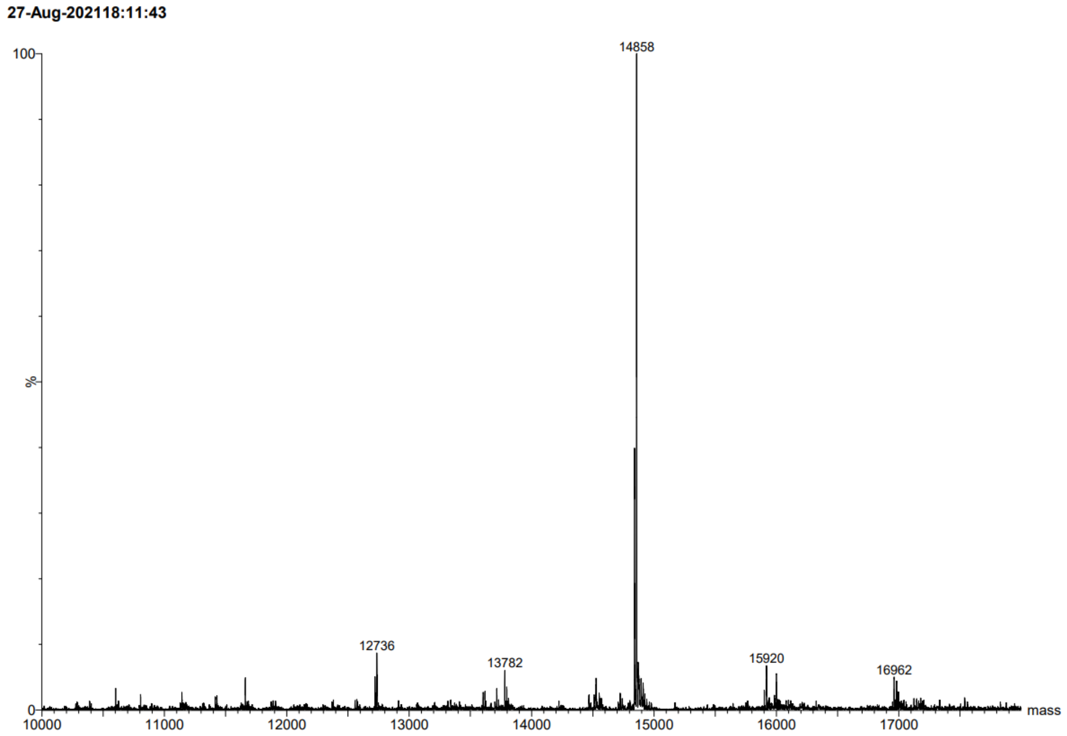


**Figure S29.** Mass spectrum of ODN9. The calculated molecular weight is 14858.1 Da, and the observed molecular weight is 14858 Da.

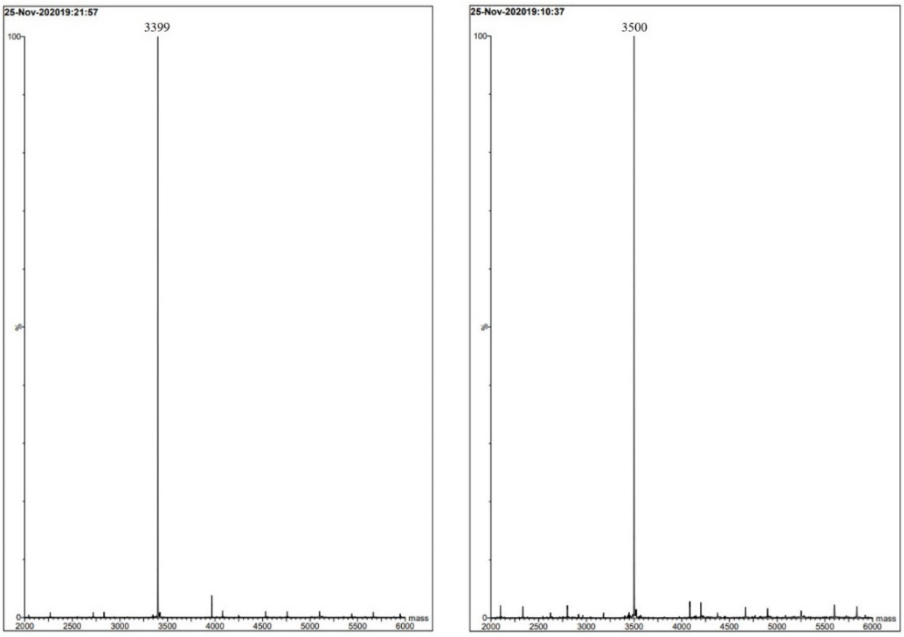


**Figure S30.** Mass spectra of ODN16 (left) and ODN17 (right). The calculated molecular weight of ODN16 is 3397.2 Da, and the observed molecular weight is 3399 Da. The calculated molecular weight of ODN17 is 3498.8 Da, and the observed molecular weight is 3500 Da.

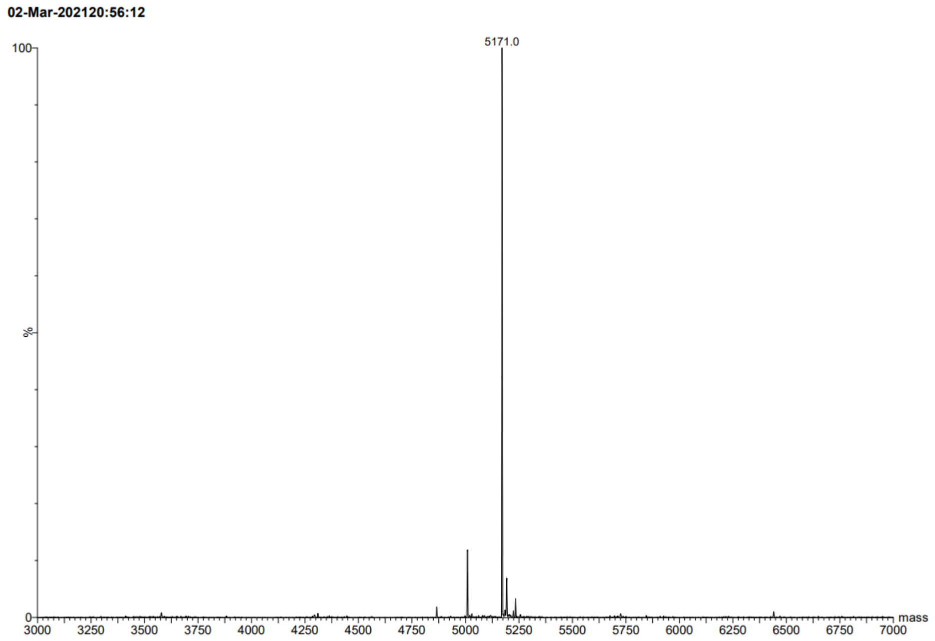


**Figure S31.** Mass spectrum of ODN18. The calculated molecular weight is 5169.7 Da, and the observed molecular weight is 5171.0 Da.

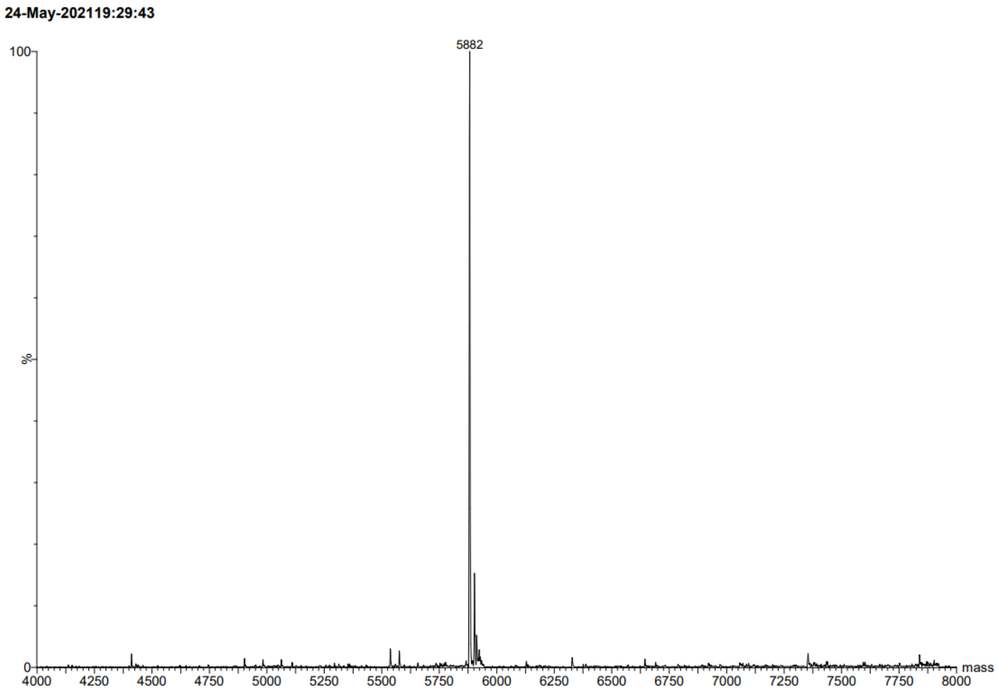


**Figure S32.** Mass spectrum of ODN19. The calculated molecular weight is 5880.3 Da, and the observed molecular weight is 5882 Da.

## 5. Synthesis of phosphoramidites

### Synthesis of Val-Ala-02 phosphoramidite

*Synthesis of Fmoc-Val-Ala-OH*

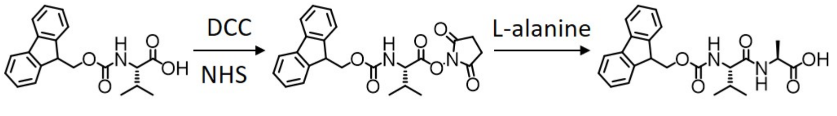


Fmoc-Val-OH Fmoc-Val-NHS Fmoc-Val-Ala-OH

Fmoc-Val-OH (4.0 g, 11.8 mmol), NHS (1.44 g, 12.36 mmol) and DCC (2.56 g, 12.36 mmol) were dissolved in THF (100 mL), and the mixture was stirred at room temperature overnight. Then, the mixture was filtered and the precipitates were washed with THF. The filtrate was collected, and the organic solvent was removed under reduced pressure. After purification by silica gel column chromatography (elution solution: $CH_2Cl_2$/methanol 30:1 v/v), Fmoc-Val-NHS (4.69 g, 10.7 mmol) was obtained as colorless solid (90.6% yield). $^1$H NMR (400 MHz, DMSO-*d6*) δ 8.16 (d, *J* = 8.4 Hz, 1H), 7.90 (d, *J* = 7.5 Hz, 2H), 7.79-7.71 (m, 2H), 7.43 (t, *J* = 7.4 Hz, 2H), 7.37-7.30 (m, 2H), 4.40-4.31 (m, 3H), 4.27 (dd, *J* = 14.6, 7.5 Hz, 1H), 2.82 (s, 4H), 2.21 (dq, *J* = 13.5, 6.7 Hz, 1H), 1.03 (dd, *J* = 6.7, 1.2 Hz, 6H).

Fmoc-Val-NHS (4.69 g, 10.7 mmol) was dissolved in 30 mL THF. Then, a solution of 1.0 g L-alanine (11.3 mmol) and 1.0 g $NaHCO_3$ (11.3 mmol) in 30 mL $H_2O$ was added to the above THF solution. Immediately, the colorless solution turned turbid. Then, a mixture of $H_2O$, THF and diethyl ether (40 mL, 1:1:1 v/v) was added until a clear solution occurred. The mixture was stirred at room temperature for three days. Then, the solvents were removed under reduced pressure, and 50 mL 15% citric acid aqueous solution and 50 mL ethyl acetate were added. The mixture was stirred for an additional one hour at room temperature until the white solid dissolved. The phases were separated, and the aqueous layer was extracted three times with 150 mL ethyl acetate. The organic layer was collected and dried by anhydrous $Na_2SO_4$. The solvent was removed in vacuum, and the residue was purified by silica gel column chromatography (elution solution: $CH_2Cl_2$/methanol/$CH_3COOH$ 10:1:0.01 v/v). After purification, 2.07 g of Fmoc-Val-Ala-OH was obtained as a white solid (47.1% yield). $^1$H NMR (400 MHz, DMSO-*d6*) δ 8.21 (d, *J* = 6.9 Hz, 1H), 7.89 (d, *J* = 7.5 Hz, 2H), 7.75 (t, *J* = 6.6 Hz, 2H), 7.46-7.38 (m, 3H), 7.37-7.30 (m, 2H), 4.34-4.15 (m, 4H), 3.90 (dd, *J* = 9.1, 7.2 Hz, 1H), 1.98 (dd, *J* = 13.7, 6.9 Hz, 1H), 1.27 (d, *J* = 7.3 Hz, 3H), 0.88 (dd, *J* = 13.9, 6.8 Hz, 6H).

*Synthesis of Fmoc-Val-Ala-PAP-OH*

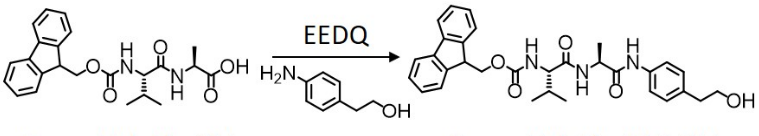


Fmoc-Val-Ala-OH (1.6 g, 3.9 mmol), 1.06 g EEDQ (4.3 mmol) and 0.59 g 4-aminophenethyl alcohol (4.3 mmol) were dissolved in 100 mL dichloromethane. Then, methanol was added until the solution turned clear. The mixture was stirred overnight, and white precipitates occurred. The solid was filtered and washed twice with dichloromethane. After drying, 1.37 g of Fmoc-Val-Ala-PAP-OH was obtained as a white solid (66.1% yield). $^{1}$H NMR (400 MHz, DMSO-*d6*) δ 9.85 (s, 1H), 8.13 (d, *J* = 7.1 Hz, 1H), 7.86 (d, *J* = 7.5 Hz, 2H), 7.71 (t, *J* = 7.2 Hz, 2H), 7.52 – 7.35 (m, 5H), 7.29 (t, *J* = 7.4 Hz, 2H), 7.10 (d, *J* = 8.5 Hz, 2H), 4.57 (t, *J* = 5.2 Hz, 1H), 4.39 (p, *J* = 6.9 Hz, 1H), 4.32 – 4.16 (m, 3H), 3.88 (dd, *J* = 8.7, 7.2 Hz, 1H), 3.52 (td, *J* = 7.1, 5.4 Hz, 2H), 2.63 (t, *J* = 7.1 Hz, 2H), 1.96 (dq, *J* = 13.6, 6.7 Hz, 1H), 1.27 (d, *J* = 7.1 Hz, 3H), 0.84 (dd, *J* = 11.8, 6.8 Hz, 6H). $^{13}$C NMR (101 MHz, DMSO-*d6*) δ 171.46, 171.29, 156.63, 144.35, 144.27, 141.25, 141.18, 137.36, 134.88, 129.52, 128.12, 127.54, 125.84, 120.58, 119.54, 66.17, 62.77, 60.48, 49.43, 47.16, 38.95, 30.86, 19.65, 18.72, 18.63.

*Synthesis of Fmoc-Val-Ala-PAP-DMT*

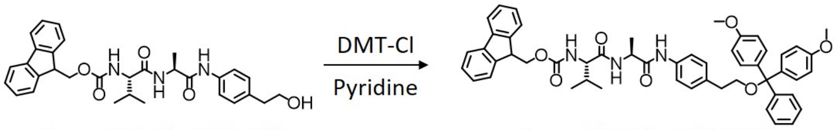


Fmoc-Val-Ala-PAP-OH (1.36 g, 2.57 mmol) was dissolved in 30 mL anhydrous pyridine under argon gas protection. Then, 1.42 g DMT-Cl (4.19 mmol) was added. The mixture was stirred for six hours and then the solvent was removed under high vacuum. After purification by silica gel column chromatography (elution solvents: EtOAc/petroleum ether 1:3 v/v to EtOAc and 1% TEA were added), 1.87 g of Fmoc-Val-Ala-PAP-DMT was obtained as a white solid (87.5% yield). $^{1}$H NMR (400 MHz, $CDCl_3$) δ 8.37 (s, 1H), 7.74 (d, *J* = 7.5 Hz, 2H), 7.57 – 7.49 (m, 2H), 7.43 (d, *J* = 8.0 Hz, 2H), 7.40 – 7.32 (m, 4H), 7.31 – 7.20 (m, 8H), 7.20 – 7.13 (m, 1H), 7.09 (d, *J* = 8.1 Hz, 2H), 6.88 – 6.68 (m, 5H), 5.45 (d, *J* = 7.9 Hz, 1H), 4.72 – 4.59 (m, 1H), 4.50 – 4.32 (m, 2H), 4.19 – 4.15 (m, 1H), 4.05 (t, *J* = 7.0 Hz, 1H), 3.76 (s, 6H), 3.23 (t, *J* = 6.9 Hz, 2H), 2.81 (t, *J* = 6.8 Hz, 2H), 2.19 – 2.07 (m, 1H), 1.43 (d, *J* = 6.9 Hz, 3H), 0.92 (t, *J* = 6.9 Hz, 6H). $^{13}$C NMR (101 MHz, $CDCl_3$) δ 170.76, 170.31, 168.87, 157.46, 144.30, 142.80, 140.46, 135.59, 134.93, 134.76, 129.12, 128.78, 127.28, 126.90, 126.84, 126.22, 125.73, 124.07, 119.14, 118.97, 112.14, 85.11, 66.19, 63.82, 59.54, 54.32, 48.69, 46.30, 35.29, 30.14, 18.31, 17.03, 16.65.

*Synthesis of Val-Ala-PAP-DMT*

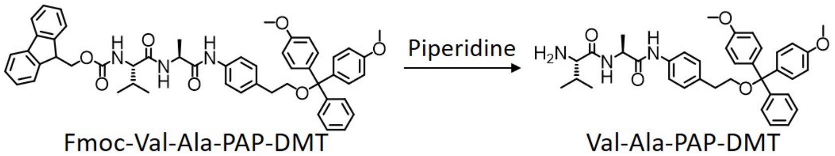


Fmoc-Val-Ala-PAP-DMT Val-Ala-PAP-DMT

Fmoc-Val-Ala-PAP-DMT (1.85 g, 2.23 mmol) was dissolved in 24 mL DMF and 6 mL of piperidine was added. The mixture was stirred for 30 minutes at room temperature. Then, the organic solvent was removed under high vacuum. After purification by silica gel column chromatography (elution solvents: EtOAc to $CH_2Cl_2$/methanol 20:1 v/v and 1% TEA were added), 1.35 g of Val-Ala-PAP-DMT was obtained as a solid foam (99.1% yield). $^1$H NMR (400 MHz, $CDCl_3$) δ 8.78 (s, 1H), 7.82 (d, *J* = 7.7 Hz, 1H), 7.37 (d, *J* = 8.4 Hz, 2H), 7.27 (d, *J* = 7.2 Hz, 2H), 7.21 – 7.13 (m, 6H), 7.10 (t, *J* = 7.2 Hz, 1H), 7.05 (d, *J* = 8.4 Hz, 2H), 6.75 – 6.66 (m, 4H), 4.56 (p, *J* = 7.0 Hz, 1H), 3.70 (s, 6H), 3.23 (d, *J* = 3.6 Hz, 1H), 3.16 (t, *J* = 6.8 Hz, 2H), 2.75 (t, *J* = 6.8 Hz, 2H), 2.25 (dtd, *J* = 13.8, 7.0, 3.8 Hz, 1H), 1.39 (d, *J* = 7.0 Hz, 3H), 0.93 (d, *J* = 7.0 Hz, 3H), 0.77 (d, *J* = 6.9 Hz, 3H). $^{13}$C NMR (101 MHz, $CDCl_3$) δ 175.47, 169.99, 158.33, 145.18, 136.49, 136.47, 136.18, 135.35, 130.00, 129.62, 128.17, 127.71, 126.58, 119.68, 113.00, 85.96, 64.71, 59.87, 55.20, 49.24, 36.16, 30.86, 19.62, 16.94, 16.01.

*Synthesis of HDA-NHS*

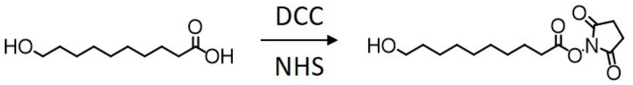


10-Hydroxydecanoic acid HDA-NHS

10-Hydroxydecanoic acid (2.0 g, 10.6 mmol), NHS (1.28 g, 11.15 mmol) and DCC (2.30 g, 11.15 mmol) were dissolved in THF (100 mL), and the mixture was stirred at room temperature overnight. Afterwards, the precipitate was filtered and washed with THF. The filtrate was collected, and the organic solvent was removed under reduced pressure. After purification by silica gel column chromatography (elution solvents: EtOAc/petroleum ether 1:2 to 1:1 v/v), 2.03 g of HDA-NHS (7.11 mmol) was obtained as a white solid (67.1% yield). $^1$H NMR (400 MHz, $CDCl_3$) δ 3.63 (t, *J* = 6.6 Hz, 2H), 2.83 (s, 4H), 2.59 (t, *J* = 7.4 Hz, 2H), 1.74 (dt, *J* = 15.2, 7.4 Hz, 2H), 1.60 – 1.50 (m, 2H), 1.44 – 1.36 (m, 2H), 1.31 (s, 8H). $^{13}$C NMR (101 MHz, $CDCl_3$) δ 169.22, 168.69, 63.01, 32.74, 30.93, 29.22, 28.92, 28.69, 25.64, 25.60, 24.54.

*Synthesis of HDA-Val-Ala-PAP-DMT*

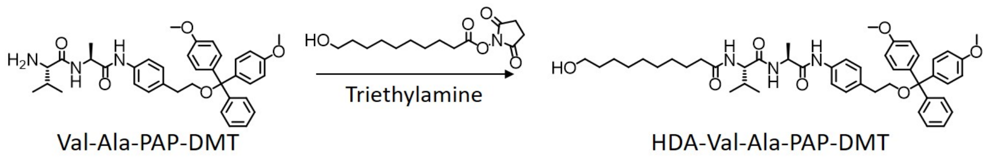


Val-Ala-PAP-DMT (1.34 g, 2.20 mmol), 0.83 g HDA-NHS (2.92 mmol) and 0.6 mL triethylamine were dissolved in 40 mL dichloromethane. The mixture was stirred overnight under argon gas protection at room temperature. Then, the organic solvent was removed under reduced pressure. After purification by silica gel column chromatography (elution solvents: EtOAc to $CH_2Cl_2$/methanol 20:1 v/v and 1% TEA were added), 1.51 g of HDA-Val-Ala-PAP-DMT was obtained as a solid foam (87.7% yield). $^1$H NMR (400 MHz, $CDCl_3$) δ 8.88 (s, 1H), 7.60 (d, *J* = 7.8 Hz, 1H), 7.46 (d, *J* = 8.5 Hz, 2H), 7.34 (d, *J* = 7.2 Hz, 2H), 7.29 – 7.19 (m, 6H), 7.17 (dt, *J* = 8.3, 3.6 Hz, 1H), 7.09 (d, *J* = 8.5 Hz, 2H), 6.86 – 6.74 (m, 4H), 6.70 (d, *J* = 8.8 Hz, 1H), 4.77 (p, *J* = 7.0 Hz, 1H), 4.56 – 4.41 (m, 1H), 3.76 (s, 7H), 3.61 (t, *J* = 6.6 Hz, 2H), 3.23 (t, *J* = 6.9 Hz, 2H), 2.82 (t, *J* = 6.8 Hz, 2H), 2.27 (t, *J* = 7.6 Hz, 2H), 2.09 – 1.94 (m, 1H), 1.67 – 1.47 (m, 4H), 1.43 (d, *J* = 7.0 Hz, 3H), 1.24 (s, 10H), 0.91 (dd, *J* = 6.6, 4.8 Hz, 6H). $^{13}$C NMR (101 MHz, $CDCl_3$) δ 173.68, 171.88, 170.38, 158.33, 145.16, 136.46, 136.11, 135.47, 129.99, 129.55, 128.15, 127.71, 126.59, 119.89, 113.01, 86.00, 64.75, 62.89, 58.22, 55.19, 49.52, 36.56, 36.18, 32.72, 31.51, 29.28, 29.20, 29.09, 29.07, 25.75, 25.65, 19.19, 18.48, 17.96.

*Synthesis of Val-Ala-02 phosphoramidite*

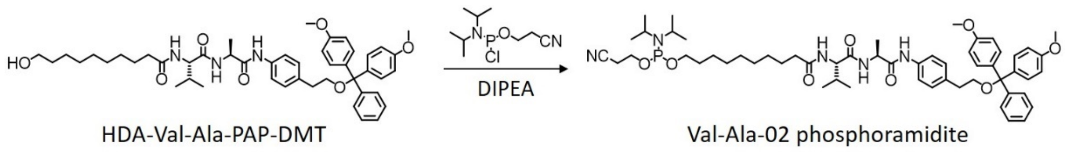


HDA-Val-Ala-PAP-DMT (0.75 g, 0.96 mmol) was dissolved in 30 mL anhydrous dichloromethane under argon gas protection. Then, 0.8 mL N,N-diisopropylethylamine were added. The mixture was allowed to cool down in an ice bath. Then, 0.5 mL 2-cyanoethyl N,N-diisopropylchlorophosphor-amidite were added. The reaction was monitored by thin-layer chromatography. When the reaction was complete, the mixture was washed with saturated $NaHCO_3$ and brine. The organic layer was collected and dried with anhydrous $MgSO_4$. After purification by silica gel column chromatography (elution solvents: EtOAc (1% TEA)), 0.61 g of Val-Ala-02 phosphoramidite was obtained as a colorless solid foam (64.6% yield). $^1$H NMR (400 MHz, $CDCl_3$) δ 8.45 (s, 1H), 7.40 (d, *J* = 8.5 Hz, 2H), 7.31 (dt, *J* = 3.4, 1.9 Hz, 2H), 7.26 – 7.17 (m, 6H), 7.16 – 7.11 (m, 1H), 7.08 (d, *J* = 8.5 Hz, 2H), 6.96 (d, *J* = 7.5 Hz, 1H), 6.83 – 6.67 (m, 4H), 6.25 (d, *J* = 8.4 Hz, 1H), 4.63 (p, *J* = 7.0 Hz, 1H), 4.32 (dd, *J* = 8.2, 7.2 Hz, 1H), 3.87 –

3.68 (m, 8H), 3.57 (m, 4H), 3.20 (t, *J* = 6.9 Hz, 2H), 2.79 (t, *J* = 6.8 Hz, 2H), 2.60 (t, *J* = 6.5 Hz, 2H), 2.20 (dd, *J* = 15.2, 7.8 Hz, 2H), 2.06 (dd, *J* = 13.6, 6.8 Hz, 1H), 1.57 (td, *J* = 13.9, 6.8 Hz, 4H), 1.41 (d, *J* = 7.0 Hz, 3H), 1.23 (m, 10H), 1.15 (dd, *J* = 6.8, 4.5 Hz, 12H), 0.89 (d, *J* = 6.8 Hz, 6H). $^{31}$P NMR (162 MHz, $CDCl_3$) δ 147.23.

**Synthesis of Val-Ala-01 phosphoramidite**

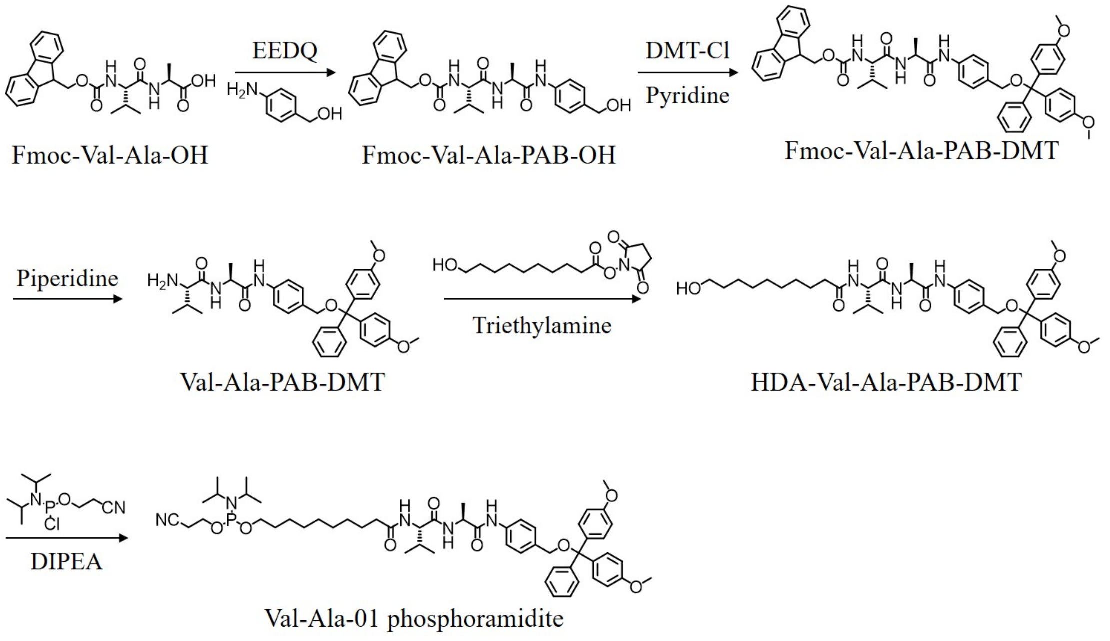


**Figure S33.** Synthesis of Val-Ala-01 phosphoramidite.

*Synthesis of Fmoc-Val-Ala-PAB-OH*

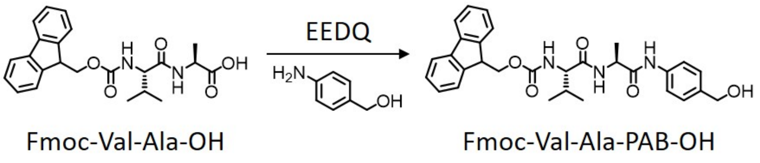


Fmoc-Val-Ala-OH (2.07 g, 5.06 mmol), 4-aminobenzyl alcohol (0.75 g, 6.08 mmol) and 1.50 g EEDQ (6.08 mmol) were dissolved in 50 mL dichloromethane under nitrogen gas protection. Methanol was dropwise added to the mixture until a clear solution was obtained. The reaction was stirred overnight, after which a precipitate was formed. After filtration, the precipitate was washed with diethyl ether and sonicated for 15 min at room temperature. This procedure was repeated twice, and the white solid of Fmoc-Val-Ala-PAB-OH (1.59 g, 3.08 mmol) was dried under high vacuum (60.9% yield). $^{1}$H NMR (400 MHz, DMSO-*d6*) δ 9.93 (s, 1H), 8.18 (d, *J* = 7.0 Hz, 1H), 7.90 (d, *J* = 7.5 Hz, 2H), 7.75 (t, *J* = 7.2 Hz, 2H), 7.54 (d, *J* = 8.4 Hz, 2H), 7.48 – 7.39 (m, 3H), 7.33 (t, *J* = 7.4 Hz, 2H), 7.24 (d, *J* = 8.5 Hz, 2H), 5.11 (t, *J* = 5.6 Hz, 1H), 4.44 (d, *J* = 5.2 Hz, 3H), 4.31 (dd, *J* = 12.5, 10.0 Hz, 1H), 4.23 (d, *J* = 5.7 Hz, 2H), 3.97 – 3.87

(m, 1H), 2.00 (dq, *J* = 13.5, 6.7 Hz, 1H), 1.31 (d, *J* = 7.1 Hz, 3H), 0.88 (dd, *J* = 12.5, 6.8 Hz, 6H). $^{13}$C NMR (101 MHz, DMSO-*d*6) δ 171.48, 171.37, 156.63, 144.35, 144.26, 141.18, 138.03, 137.90, 128.12, 127.54, 127.38, 125.84, 120.58, 119.34, 66.17, 63.06, 60.48, 49.46, 47.15, 30.86, 19.66, 18.72, 18.60.

*Synthesis of Fmoc-Val-Ala-PAB-DMT*

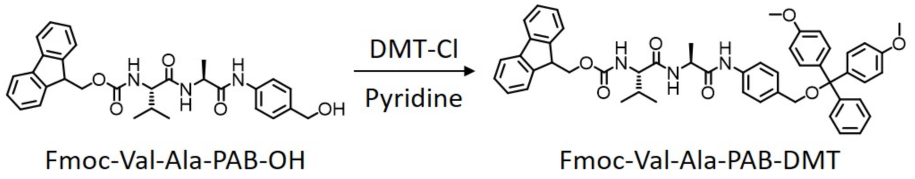


Fmoc-Val-Ala-PAB-OH (0.71 g, 1.38 mmol) was dissolved in 30 mL anhydrous pyridine under the protection of argon gas. Then, 4,4'-dimethoxytrityl chloride (0.70 g, 2.06 mmol) was added. The mixture was stirred overnight, and then the organic solvent was removed under high vacuum. After purification by silica gel column chromatography (elution solvents: EtOAc/petroleum ether 1:3 v/v to ethyl acetate and 1% TEA were added), 0.82 g of Fmoc-Val-Ala-PAB-DMT (1.0 mmol) was obtained as a solid foam (72.5% yield). $^{1}$H NMR (400 MHz, $CDCl_3$) δ 8.41 (s, 1H), 7.74 (d, *J* = 7.5 Hz, 2H), 7.61 – 7.46 (m, 6H), 7.44 – 7.34 (m, 6H), 7.32 – 7.25 (m, 6H), 7.23 – 7.18 (m, 1H), 6.87 – 6.76 (m, 4H), 6.68 (d, *J* = 7.2 Hz, 1H), 5.40 (d, *J* = 7.5 Hz, 1H), 4.72 – 4.62 (m, 1H), 4.52 – 4.39 (m, 2H), 4.20 (t, *J* = 6.6 Hz, 1H), 4.10 – 4.00 (m, 3H), 3.79 (s, 6H), 2.15 (td, *J* = 13.2, 7.1 Hz, 1H), 1.46 (d, *J* = 6.9 Hz, 3H), 0.94 (t, *J* = 7.6 Hz, 6H). $^{13}$C NMR (101 MHz, $CDCl_3$) δ 171.64, 158.48, 145.06, 143.65, 141.35, 136.32, 130.08, 128.22, 127.83, 127.79, 127.70, 127.13, 127.11, 126.75, 124.96, 124.93, 120.03, 119.82, 113.13, 100.00, 67.10, 65.24, 60.41, 55.22, 49.60, 47.20, 30.95, 19.19, 17.47, 14.20.

*Synthesis of Val-Ala-PAB-DMT*

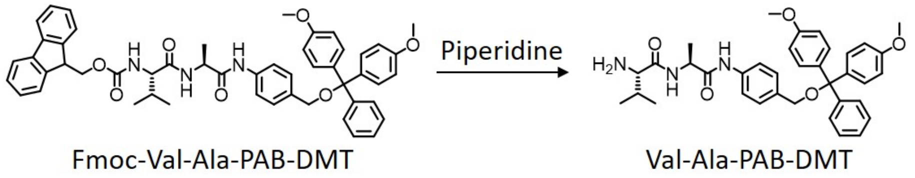


Fmoc-Val-Ala-PAB-DMT (0.81 g, 0.99 mmol) was dissolved in 20 mL DMF solution and 5 mL piperidine was added. The mixture was stirred for 30 min at room temperature. Then, the solvent was removed under high vacuum, and the residue was purified by silica gel column chromatography (elution solvents: $CH_2Cl_2$/MeOH 20:1 v/v and 1% TEA was added). After purification, 0.59 g of Val-Ala-PAB-DMT (0.99 mmol) was obtained as a solid foam (100% yield). $^{1}$H NMR (400 MHz, $CDCl_3$) δ 8.87 (s, 1H), 7.84 (d, *J* = 7.7 Hz, 1H), 7.50 – 7.40 (m, 4H), 7.37 – 7.29 (m, 4H), 7.27 – 7.19 (m, 4H), 7.20 – 7.09 (m, 1H),

6.84 – 6.73 (m, 4H), 4.59 (p, *J* = 7.0 Hz, 1H), 4.04 (s, 2H), 3.73 (s, 6H), 3.25 (d, *J* = 3.7 Hz, 1H), 2.27 (dtd, *J* = 13.9, 6.9, 3.8 Hz, 1H), 1.41 (d, *J* = 7.0 Hz, 3H), 0.95 (d, *J* = 7.0 Hz, 3H), 0.80 (d, *J* = 6.9 Hz, 3H). $^{13}C$ NMR (101 MHz, $CDCl_3$) δ 175.49, 170.10, 158.46, 145.08, 137.03, 136.35, 135.09, 130.07, 128.21, 127.82, 127.71, 126.73, 119.68, 113.12, 86.38, 65.31, 59.90, 55.22, 49.27, 30.87, 19.62, 16.95, 16.03.

*Synthesis of HDA-Val-Ala-PAB-DMT*

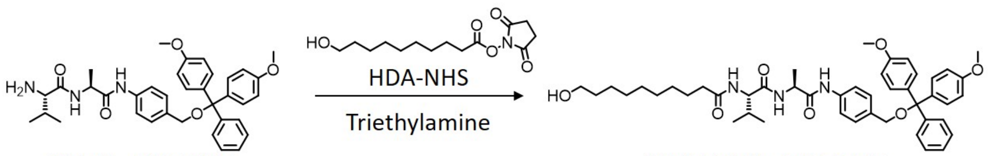


Val-Ala-PAB-DMT (0.59 g, 0.99 mmol), 0.2 mL triethylamine and 0.38 g HDA-NHS (1.33 mmol) were dissolved in 30 mL dichloromethane, and the mixture was stirred overnight. Then, the organic solvent was removed under reduced pressure. After purification by silica gel column chromatography (elution solvents: EtOAc to $CH_2Cl_2$/MeOH 20:1 v/v and 1% TEA was added), 0.66 g of HDA-Val-Ala-PAB-DMT (0.86 mmol) was obtained as a solid foam (86.9% yield). $^1H$ NMR (400 MHz, $CDCl_3$) δ 8.88 (s, 1H), 7.54 (d, *J* = 8.5 Hz, 2H), 7.47 (dd, *J* = 12.4, 5.2 Hz, 3H), 7.41 – 7.34 (m, 4H), 7.28 (dd, *J* = 16.5, 8.2 Hz, 4H), 7.22 – 7.16 (m, 1H), 6.86 – 6.77 (m, 4H), 6.60 (d, *J* = 8.7 Hz, 1H), 4.76 (p, *J* = 7.1 Hz, 1H), 4.50 – 4.40 (m, 1H), 4.10 (s, 2H), 3.78 (d, *J* = 4.3 Hz, 6H), 3.59 (t, *J* = 6.6 Hz, 2H), 2.28 (dd, *J* = 8.0, 6.3 Hz, 2H), 2.06 (dt, *J* = 20.7, 6.8 Hz, 1H), 1.68 – 1.59 (m, 2H), 1.51 (dd, *J* = 14.1, 6.9 Hz, 2H), 1.46 (d, *J* = 7.0 Hz, 3H), 1.25 (s, 10H), 0.94 (dd, *J* = 6.7, 2.8 Hz, 6H).

*Synthesis of Val-Ala-01 phosphoramidite*

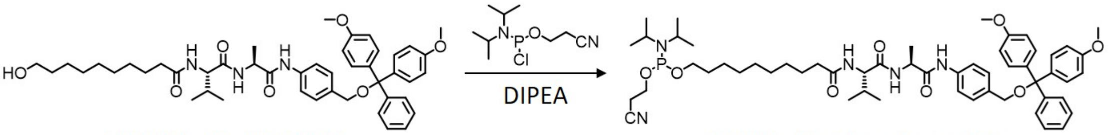


HDA-Val-Ala-PAB-DMT (0.65 g, 0.84 mmol) was dissolved in 30 mL anhydrous dichloromethane under the protection of argon gas. Then, 1.0 mL DIPEA was added. The mixture was allowed to cool down in an ice bath and then 0.6 mL 2-cyanoethyl N,N-diisopropylchlorophosphoramidite was added. The reaction was monitored by thin-layer chromatography. When the reaction complete, the mixture was washed by saturated $NaHCO_3$ and brine. The organic layer was collected and dried by anhydrous $Na_2SO_4$. After purification by silica gel column chromatography (elution solvent: ethyl acetate (1% TEA)), 0.45 g of Val-Ala-01 phosphoramidite (0.46 mmol) was obtained as a solid foam (54.7% yield). $^1H$

NMR (400 MHz, $CDCl_3$) δ 8.55 (s, 1H), 7.50 (dd, *J* = 14.8, 7.8 Hz, 4H), 7.41 – 7.34 (m, 4H), 7.29 (dd, *J* = 12.5, 5.1 Hz, 4H), 7.24 – 7.16 (m, 1H), 6.98 (d, *J* = 7.5 Hz, 1H), 6.86 – 6.79 (m, 4H), 6.24 (d, *J* = 8.3 Hz, 1H), 4.66 (p, *J* = 7.1 Hz, 1H), 4.38 – 4.30 (m, 1H), 4.10 (s, 2H), 3.78 (s, 6H), 3.69 – 3.50 (m, 4H), 2.62 (dt, *J* = 9.1, 6.4 Hz, 2H), 2.29 – 2.22 (m, 2H), 2.10 (dq, *J* = 13.6, 6.8 Hz, 1H), 1.64 (s, 6H), 1.46 (d, *J* = 7.0 Hz, 3H), 1.35 – 1.26 (m, 10H), 1.17 (dd, *J* = 6.8, 4.8 Hz, 12H), 0.95 (dd, *J* = 6.8, 2.5 Hz, 6H). $^{31}P$ NMR (162 MHz, $CDCl_3$) δ 147.24.

**Synthesis of Val-Ala-Coumarin phosphoramidite**

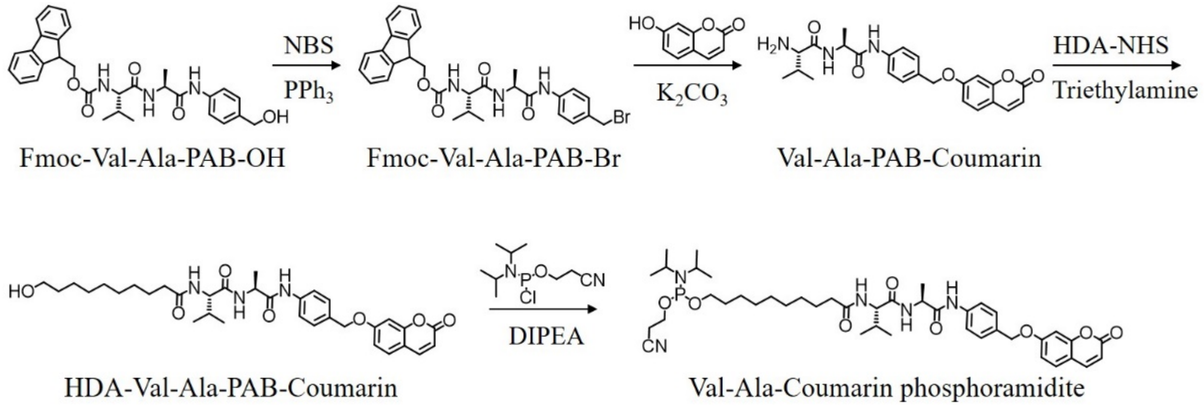


**Figure S34.** Synthesis of Val-Ala-Coumarin phosphoramidite. NBS: N-bromosuccinimide. $PPh_3$: triphenylphosphine. DIPEA: N,N-diisopropylethylamine.

*Synthesis of Fmoc-Val-Ala-PAB-Br*

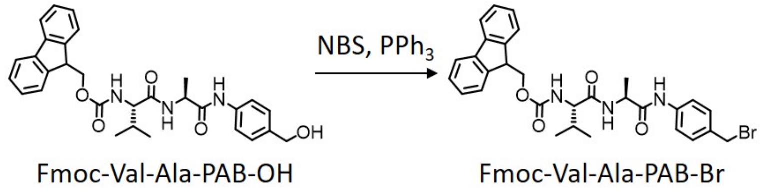


Fmoc-Val-Ala-PAB-OH (0.9 g, 1.74 mmol) and triphenylphosphine (0.91 g, 3.49 mmol) were dissolved in 100 mL dry THF under argon gas protection. The reaction was allowed to cool down in an ice bath. Then, 0.62 g *N*-bromosuccinimide (3.49 mmol) was added. After stirring at room temperature for 0.5 hours, the organic solvent was removed under reduced pressure. The residue was dissolved in ethyl acetate and washed by saturated $NaHCO_3$ and brine. The organic layers were collected and dried by anhydrous $Na_2SO_4$. Fmoc-Val-Ala-PAB-Br was used for the next step without further purification.

*Synthesis of Val-Ala-PAB-Coumarin*

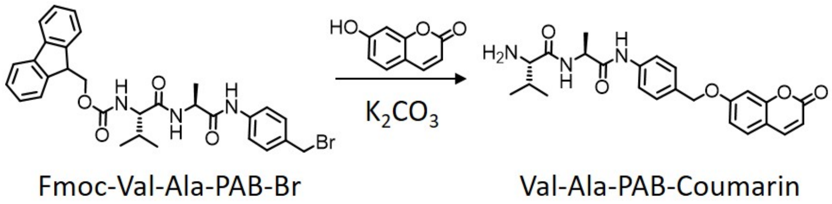


7-Hydroxycoumarin (0.17 g, 1.04 mmol) was dissolved in 10 mL anhydrous DMF, and 0.14 g anhydrous $K_2CO_3$ was added. The mixture was stirred for 30 minutes under argon gas protection. Then, Fmoc-Val-Ala-PAB-Br (0.3 g, 0.52 mmol) in 5 mL anhydrous DMF was added. The mixture was stirred overnight under room temperature. Then, the mixture was poured into saturated $NaHCO_3$ solution and extracted with 100 mL EtOAc. The organic layer was collected and dried by $Na_2SO_4$. After purification by silica gel column chromatography (elution solvents: ethyl acetate to $CH_2Cl_2$/methanol 3:1 v/v), 108 mg Val-Ala-PAB-Coumarin was obtained as a light-yellow solid (47.3% yield). $^1$H NMR (400 MHz, DMSO-*d6*) δ 10.12 (s, 1H), 8.22 (d, *J* = 6.2 Hz, 1H), 7.98 (d, *J* = 9.5 Hz, 1H), 7.62 (dd, *J* = 8.6, 2.3 Hz, 3H), 7.41 (d, *J* = 8.6 Hz, 2H), 7.07 (d, *J* = 2.4 Hz, 1H), 7.00 (dd, *J* = 8.6, 2.4 Hz, 1H), 6.29 (d, *J* = 9.5 Hz, 1H), 5.15 (s, 2H), 4.55 – 4.41 (m, 1H), 3.07 (d, *J* = 5.0 Hz, 1H), 2.00 – 1.87 (m, 1H), 1.31 (d, *J* = 7.0 Hz, 3H), 0.89 (d, *J* = 6.9 Hz, 3H), 0.80 (d, *J* = 6.8 Hz, 3H).

*Synthesis of HDA-Val-Ala-PAB-Coumarin*

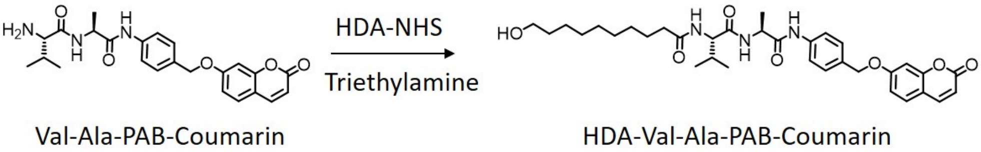


Val-Ala-PAB-Coumarin (0.3 g, 0.68 mmol), 0.1 mL triethylamine and 0.29 g HDA-NHS (1.02 mmol) were added to a 50 mL flask with 30 mL dichloromethane. Methanol was dropwise added until the solid completely dissolved. The mixture was stirred overnight under room temperature. Then, the organic solvent was removed under reduced pressure, and the residue was purified by silica gel column chromatography (elution solvents: EtOAc to $CH_2Cl_2$/methanol 10:1 v/v). After purification, 0.28 g of HDA-Val-Ala-PAB-Coumarin (0.46 mmol) was obtained as a light-yellow solid (67.6% yield). $^1$H NMR (400 MHz, DMSO-*d6*) δ 9.96 (s, 1H), 8.15 (d, *J* = 7.0 Hz, 1H), 7.98 (d, *J* = 9.5 Hz, 1H), 7.81 (d, *J* = 8.6 Hz, 1H), 7.63 (d, *J* = 8.6 Hz, 3H), 7.41 (d, *J* = 8.6 Hz, 2H), 7.06 (d, *J* = 2.3 Hz, 1H), 7.00 (dd, *J* = 8.6, 2.4 Hz, 1H), 6.28 (d, *J* = 9.5 Hz, 1H), 5.15 (s, 2H), 4.39 (p, *J* = 7.0 Hz, 1H), 4.31 (t, *J* = 5.2 Hz, 1H), 4.17 (dd, *J* = 8.5, 7.0 Hz, 1H), 3.36 (dd, *J* = 11.7, 6.5 Hz, 2H), 2.24 – 2.07 (m, 2H), 1.97 (dq, *J* = 13.5, 6.7 Hz, 1H), 1.47 (m, 2H), 1.38 (m, *J* = 12.9, 6.3 Hz, 2H), 1.30 (d, *J* = 7.1 Hz, 3H), 1.23 (m, 10H), 0.85 (dd, *J* = 14.5, 6.8 Hz, 6H).

*Synthesis of Val-Ala-Coumarin phosphoramidite*

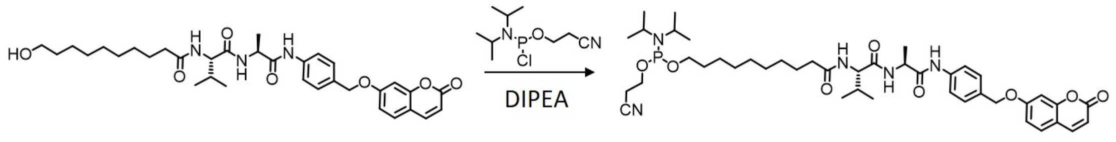


HDA-Val-Ala-PAB-Coumarin (0.26 g, 0.42 mmol) and 0.8 mL DIPEA were added to a 50 mL flask with 30 mL dry dichloromethane under argon gas protection. The mixture was cooled down in an ice bath. Then, 0.4 mL 2-cyanoethyl N,N-diisopropylchlorophosphoramidite was added. The mixture was stirred for 20 minutes. Then, the mixture was diluted with 100 mL dichloromethane and washed by saturated $NaHCO_3$ and brine. The organic layer was collected and dried by $Na_2SO_4$ and removed under reduced pressure. After purification by silica gel column chromatography (elution solvent: EtOAc (1% TEA)), 0.23 g of Val-Ala-Coumarin phosphoramidite was obtained as a light-yellow solid. $^1$H NMR (400 MHz, DMSO-*d6*) δ 10.00 (s, 1H), 8.19 (d, *J* = 7.0 Hz, 1H), 8.04 (d, *J* = 9.5 Hz, 1H), 7.85 (d, *J* = 8.6 Hz, 1H), 7.69 (d, *J* = 8.4 Hz, 3H), 7.47 (d, *J* = 8.6 Hz, 2H), 7.12 (d, *J* = 2.3 Hz, 1H), 7.06 (dd, *J* = 8.6, 2.4 Hz, 1H), 6.34 (d, *J* = 9.5 Hz, 1H), 5.21 (s, 2H), 4.45 (p, *J* = 7.0 Hz, 1H), 4.23 (dd, *J* = 8.5, 6.9 Hz, 1H), 3.84 – 3.70 (m, 2H), 3.62 (m, 4H), 2.80 (t, *J* = 5.9 Hz, 2H), 2.30 – 2.13 (m, 2H), 2.03 (dq, *J* = 13.6, 6.7 Hz, 1H), 1.57 (m, 4H), 1.37 (d, *J* = 7.1 Hz, 3H), 1.28 (m, 10H), 1.18 (dd, *J* = 6.7, 4.3 Hz, 12H), 0.91 (dd, *J* = 13.9, 6.8 Hz, 6H). $^{31}$P NMR (162 MHz, DMSO-*d6*) δ 146.37.

**Synthesis of Val-Ala-Chalcone phosphoramidite**

*Synthesis of chalcone*

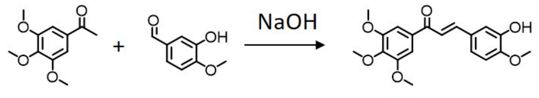


3,4,5-Trimethoxyacetophenone (1.5 g, 7.13 mmol) and 3-hydroxy-4-methoxybenzaldehyde (1.09 g, 7.16 mmol) were dissolved in 22 mL ethanol. Then, 11.4 mL aqueous NaOH (10% w/v, 4 equiv.) was added to the above solution. The mixture was stirred for 24 hours and then poured into water. 1 N HCl was added to adjust the aqueous pH to 4.0. Then, a yellow precipitate was formed. The precipitate was filtered, washed with water, and purified by silica gel column chromatography (elution solvent: $CH_2Cl_2$/methanol 20:1 v/v). After purification, 1.85 g of chalcone (5.37 mmol) was obtained as a yellow solid (75.3% yield). $^1$H NMR (400 MHz, DMSO-*d6*): δ 9.17 (s, 1H), 7.67 (q, *J* = 15.4 Hz, 2H), 7.41 (s, 2H), 7.38 (d, *J* = 2.1 Hz, 1H), 7.30 (dd, *J* = 8.4, 2.1 Hz, 1H), 7.00 (d, *J* = 8.4 Hz, 1H), 3.90 (s, 6H), 3.85 (s, 3H),

3.77 (s, 3H). $^{13}$C NMR (101 MHz, DMSO-*d6*): δ 188.23, 153.36, 150.77, 147.11, 144.86, 142.23, 133.79, 128.16, 122.84, 119.74, 115.35, 112.32, 106.49, 60.65, 56.66, 56.17.

*Synthesis of TBS-Thymidine*

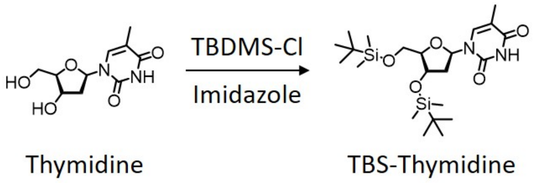


Thymidine (2.8 g, 11.56 mmol), 2.36 g imidazole (34.68 mmol) and 3.83 g TBDMS-Cl (25.4 mmol) were dissolved in 60 mL dry DMF under the protection of argon gas. The mixture was stirred overnight. Then, the solvent was removed by high vacuum. The residue was dissolved in 150 mL $CH_2Cl_2$ and washed three times with saturated brine (150 mL) solution. The organic layer was collected and dried by anhydrous $Na_2SO_4$. After removing organic solvents under reduced pressure, TBS-Thymidine was obtained and used for the next step without further purification. $^1$H NMR (400 MHz, $CDCl_3$) δ 8.58 (s, 1H), 7.47 (d, *J* = 1.2 Hz, 1H), 6.33 (dd, *J* = 7.9, 5.8 Hz, 1H), 4.42 – 4.38 (m, 1H), 3.93 (q, *J* = 2.5 Hz, 1H), 3.87 (dd, *J* = 11.4, 2.6 Hz, 1H), 3.76 (dd, *J* = 11.4, 2.4 Hz, 1H), 2.24 (ddd, *J* = 13.1, 5.8, 2.6 Hz, 1H), 2.04 – 1.96 (m, 1H), 1.91 (d, *J* = 1.2 Hz, 3H), 0.93 (s, 9H), 0.89 (s, 9H), 0.11 (d, *J* = 1.0 Hz, 6H), 0.07 (d, *J* = 2.8 Hz, 6H). $^{13}$C NMR (101 MHz, $CDCl_3$) δ 163.79, 150.34, 135.62, 110.95, 87.98, 84.98, 72.41, 63.13, 41.52, 26.08, 25.89, 18.55, 18.15, 12.67, -4.50, -4.69, -5.22, -5.31.

*Synthesis of TBS-Thymidine-Triazole*

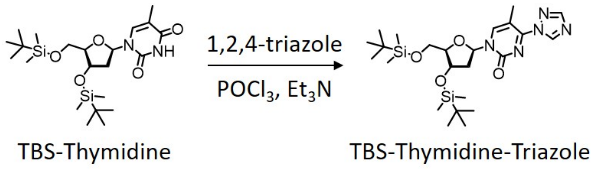


1,2,4-Triazole (2.74 g, 39.5 mmol) was suspended in 30 mL dry acetonitrile. The mixture was cooled down in an ice bath, and 0.93 mL phosphoryl chloride ($POCl_3$, 10.2 mmol) was added slowly. Then, 5.6 mL of anhydrous triethylamine (46.5 mmol) was added under vigorous stirring. After 15 minutes of stirring, a solution of 1.4 g TBS-Thymidine (2.97 mmol) in 12 mL dry acetonitrile was added over 30 minutes, and the reaction mixture was stirred overnight. Then, the solvent was removed under reduced pressure, and the residue was dissolved in $CH_2Cl_2$ then washed consecutively with saturated $NaHCO_3$ and brine. The organic layer was collected and dried by $Na_2SO_4$ and the solvent was removed under reduced pressure. TBS-Thymidine-Triazole (1.36 g, 2.6 mmol) was obtained as a yellow foam

and used for the next step without further purification (87.5% yield). $^1$H NMR (400 MHz, $CDCl_3$) δ 9.28 (s, 1H), 8.25 (d, *J* = 0.7 Hz, 1H), 8.11 (s, 1H), 6.29 (t, *J* = 6.3 Hz, 1H), 4.42 – 4.37 (m, 1H), 4.06 (dd, *J* = 5.8, 2.6 Hz, 1H), 3.96 (dd, *J* = 11.5, 2.6 Hz, 1H), 3.80 (dd, *J* = 11.5, 2.5 Hz, 1H), 2.64 (ddd, *J* = 13.5, 6.2, 3.7 Hz, 1H), 2.45 (d, *J* = 0.8 Hz, 3H), 2.08 (dt, *J* = 13.4, 6.3 Hz, 1H), 0.91 (d, *J* = 6.2 Hz, 18H), 0.10 (dd, *J* = 13.8, 5.1 Hz, 12H). $^{13}$C NMR (101 MHz, $CDCl_3$) δ 158.08, 153.93, 153.38, 146.61, 145.08, 105.24, 88.79, 87.82, 71.68, 62.62, 42.64, 25.94, 25.76, 18.42, 18.01, 17.27, -4.53, -4.87, -5.34, -5.38.

*Synthesis of Val-Ala-PAB-Chalcone*

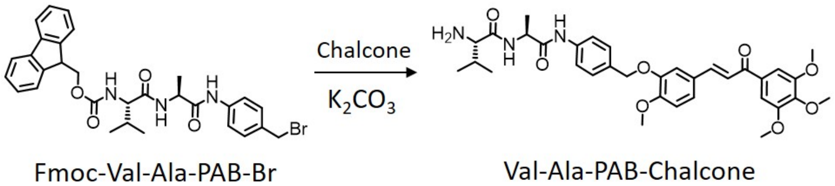


Fmoc-Val-Ala-PAB-Br Val-Ala-PAB-Chalcone

Chalcone (1.0 g, 2.90 mmol) was dissolved in 15 mL anhydrous DMF and 0.48 g anhydrous $K_2CO_3$ (3.46 mmol) was added. The mixture was stirred for 30 minutes under argon gas protection. Then, 1.12 g Fmoc-Val-Ala-PAB-Br (1.94 mmol) in 10 mL anhydrous DMF was added. The mixture was stirred overnight at room temperature. Then, the organic solvent was removed under high vacuum, and the residue was dissolved in 100 mL EtOAc. The mixture was washed with saturated $NaHCO_3$ and brine, and the organic layer was collected and dried by anhydrous $Na_2SO_4$. After purification by silica gel column chromatography (elution solvents: EtOAc to $CH_2Cl_2$/methanol 5:1 v/v), 0.46 g of Val-Ala-PAB-Chalcone was obtained as a yellow solid (38.1% yield). $^1$H NMR (400 MHz, DMSO-*d6*) δ 10.08 (s, 1H), 8.17 (s, 1H), 7.73 (dd, *J* = 35.6, 15.5 Hz, 2H), 7.61 (dd, *J* = 5.3, 3.3 Hz, 3H), 7.49 – 7.38 (m, 5H), 7.05 (d, *J* = 8.5 Hz, 1H), 5.12 (s, 2H), 4.52 – 4.41 (m, 1H), 3.91 (s, 6H), 3.82 (s, 3H), 3.77 (s, 3H), 3.17 (d, *J* = 4.9 Hz, 1H), 3.01 (d, *J* = 5.0 Hz, 1H), 1.96 – 1.85 (m, 2H), 1.30 (d, *J* = 7.0 Hz, 3H), 0.88 (d, *J* = 6.9 Hz, 3H), 0.78 (d, *J* = 6.8 Hz, 3H).

*Synthesis of HDA-Val-Ala-PAB-Chalcone*

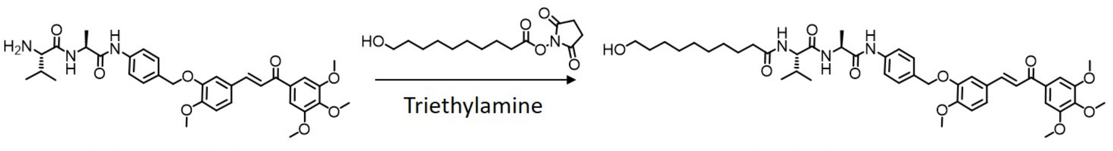


Val-Ala-PAB-Chalcone HDA-Val-Ala-PAB-Chalcone

Val-Ala-PAB-Chalcone (0.45 g, 0.73 mmol), 0.2 mL triethylamine and 0.31 g HDA-NHS (1.09 mmol) were added to a 50 mL flask with 20 mL dichloromethane. The mixture was stirred overnight under room temperature. Then, the organic solvent was removed and the residue was purified by silica gel

column chromatography (elution solvents: EtOAc to $CH_2Cl_2$/methanol 10:1 v/v). After purification, 0.22 g of HDA-Val-Ala-PAB-Chalcone (0.28 mmol) was obtained as a yellow solid (38.3% yield). $^1$H NMR (400 MHz, DMSO-*d6*) δ 8.15 (d, *J* = 6.9 Hz, 1H), 7.81 (d, *J* = 8.6 Hz, 1H), 7.73 (dd, *J* = 35.3, 15.5 Hz, 2H), 7.62 (d, *J* = 8.6 Hz, 3H), 7.48 – 7.38 (m, 5H), 7.05 (d, *J* = 8.5 Hz, 1H), 5.12 (s, 2H), 4.41 – 4.37 (m, 1H), 4.31 (t, *J* = 5.2 Hz, 1H), 4.18 (dd, *J* = 8.4, 7.0 Hz, 1H), 3.91 (s, 5H), 3.82 (s, 3H), 3.77 (s, 3H), 3.36 (dd, *J* = 11.8, 6.6 Hz, 2H), 2.23 – 2.08 (m, 2H), 1.97 (dq, *J* = 13.6, 6.7 Hz, 1H), 1.47 (s, 2H), 1.38 (dd, *J* = 13.0, 6.4 Hz, 2H), 1.30 (d, *J* = 7.1 Hz, 3H), 1.22 (s, 11H), 0.85 (dd, *J* = 14.9, 6.8 Hz, 6H). $^{13}$C NMR (101 MHz, DMSO-*d6*) δ 187.86, 172.47, 171.10, 171.03, 152.88, 151.60, 147.89, 144.26, 141.85, 138.76, 133.30, 131.61, 128.77, 127.44, 123.87, 119.64, 118.97, 113.14, 111.90, 106.16, 69.82, 60.71, 60.19, 57.52, 56.23, 55.68, 49.01, 35.12, 32.54, 30.36, 29.00, 28.90, 28.75, 28.62, 25.48, 25.37, 19.19, 18.17, 17.93.

*Synthesis of TBS-Thymidine-HDA-Val-Ala-PAB-Chalcone*

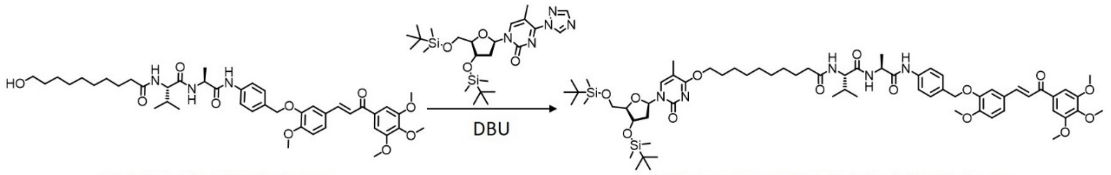


HDA-Val-Ala-PAB-Chalcone (0.45 g, 0.57 mmol), 0.36 g TBS-Thymidine-Triazole (0.68 mmol) and 0.15 mL DBU (1.0 mmol) were dissolved in 20 mL anhydrous THF under argon gas protection. The mixture was stirred overnight. Then, the solvent was removed under reduced pressure, and the residue was purified by silica gel column chromatography (elution solvents: EtOAc/petroleum ether 1:1 v/v to EtOAc). After purification, 0.32 g of TBS-Thymidine-HDA-Val-Ala-PAB-Chalcone (0.26 mmol) was obtained as a yellow solid (45.6% yield). $^1$H NMR (400 MHz, DMSO-*d6*) δ 9.95 (s, 1H), 8.13 (d, *J* = 7.0 Hz, 1H), 7.81 (d, *J* = 8.6 Hz, 1H), 7.79 – 7.65 (m, 3H), 7.62 (d, *J* = 8.6 Hz, 3H), 7.48 – 7.38 (m, 5H), 7.04 (d, *J* = 8.5 Hz, 1H), 6.13 (t, *J* = 6.7 Hz, 1H), 5.12 (s, 2H), 4.36 (ddd, *J* = 10.0, 8.8, 5.0 Hz, 2H), 4.23 (t, *J* = 6.6 Hz, 2H), 4.17 (dd, *J* = 8.5, 6.9 Hz, 1H), 3.90 (s, 6H), 3.86 (dd, *J* = 6.7, 3.7 Hz, 1H), 3.82 (s, 3H), 3.77 (s, 4H), 3.75 – 3.71 (m, 1H), 2.24 – 2.04 (m, 4H), 1.95 (dd, *J* = 13.7, 6.9 Hz, 1H), 1.86 (d, *J* = 0.8 Hz, 3H), 1.71 – 1.61 (m, 2H), 1.53 – 1.44 (m, 2H), 1.34 (s, 2H), 1.30 (d, *J* = 7.1 Hz, 3H), 1.24 (m, 8H), 0.89 – 0.80 (m, 24H), 0.07 (d, *J* = 4.1 Hz, 12H). $^{13}$C NMR (101 MHz, DMSO-*d6*) δ 187.82, 172.47, 171.06, 171.01, 169.63, 154.54, 152.87, 151.59, 147.88, 144.23, 141.84, 140.08, 138.75, 133.30, 131.60, 128.73, 127.43, 119.63, 118.95, 113.11, 111.88, 106.16, 103.09, 87.18, 85.45, 72.05, 69.82, 66.51, 62.60, 60.17, 57.56, 56.21, 55.67, 40.46, 35.10, 30.31, 28.83, 28.67, 28.60, 28.57, 27.95, 25.71, 25.63, 25.32, 19.18, 18.15, 17.94, 17.67, 11.82, -4.80, -4.97, -5.53, -5.54.

*Synthesis of Thymidine-HDA-Val-Ala-PAB-Chalcone*

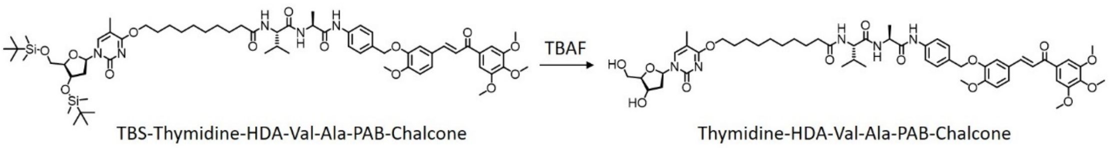


TBS-Thymidine-HDA-Val-Ala-PAB-Chalcone (0.3 g, 0.24 mmol) was dissolved in 20 mL dry THF under argon gas protection, and 0.6 mL TBAF (1 M in THF) was added. The mixture was stirred for two hours at room temperature. Then, the solvent was removed under reduced pressure, and the residue was purified by silica gel column chromatography (elution solvents: EtOAc to $CH_2Cl_2$/methanol 10:1 v/v). After purification, 0.17 g of Thymidine-HDA-Val-Ala-PAB-Chalcone (0.17 mmol) was obtained as a yellow solid (79.2% yield). $^1$H NMR (400 MHz, DMSO-*d6*) δ 9.96 (s, 1H), 8.15 (d, *J* = 7.0 Hz, 1H), 8.01 (d, *J* = 0.9 Hz, 1H), 7.82 (d, *J* = 8.6 Hz, 1H), 7.73 (dd, *J* = 36.4, 15.5 Hz, 2H), 7.63 (d, *J* = 8.5 Hz, 3H), 7.48 – 7.38 (m, 5H), 7.05 (d, *J* = 8.5 Hz, 1H), 6.14 (t, *J* = 6.5 Hz, 1H), 5.23 (d, *J* = 4.3 Hz, 1H), 5.13 (s, 2H), 5.06 (t, *J* = 5.2 Hz, 1H), 4.45 – 4.34 (m, 1H), 4.23 (t, *J* = 6.4 Hz, 3H), 4.18 (dd, *J* = 8.4, 7.0 Hz, 1H), 3.91 (s, 6H), 3.85 – 3.80 (m, 4H), 3.78 (s, 3H), 3.61 (qdd, *J* = 9.0, 7.7, 5.3 Hz, 2H), 2.23 – 2.10 (m, 3H), 1.99 (tt, *J* = 13.6, 6.6 Hz, 2H), 1.87 (d, *J* = 0.7 Hz, 3H), 1.71 – 1.63 (m, 2H), 1.49 (d, *J* = 6.2 Hz, 2H), 1.35 (s, 2H), 1.31 (d, *J* = 7.1 Hz, 3H), 1.25 (m, 8H), 0.86 (dd, *J* = 14.6, 6.8 Hz, 6H). $^{13}$C NMR (101 MHz, DMSO-*d6*) δ 187.85, 172.48, 171.09, 171.02, 169.59, 154.65, 152.88, 151.60, 147.88, 144.25, 141.84, 140.78, 138.75, 133.30, 131.61, 128.76, 127.43, 119.64, 118.97, 113.13, 111.89, 106.16, 102.95, 87.53, 85.39, 70.01, 69.81, 66.40, 61.01, 60.18, 57.56, 56.23, 55.67, 49.00, 40.56, 35.10, 30.33, 28.84, 28.68, 28.62, 28.58, 27.98, 25.34, 19.18, 18.16, 17.94, 11.81.

*Synthesis of DMT-Thymidine-HDA-Val-Ala-PAB-Chalcone*

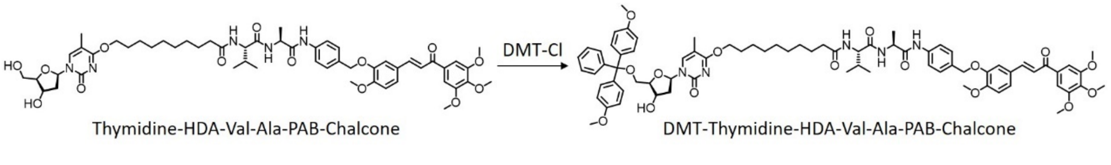


Thymidine-HDA-Val-Ala-PAB-Chalcone (0.16 g, 0.16 mmol) was dissolved in 5 mL anhydrous pyridine under argon gas protection. Then, 76 mg 4,4'-dimethoxytrityl chloride was added. The mixture was stirred overnight. Then, the solvent was removed under high vacuum. After purification by silica gel column chromatography (elution solvents: EtOAc to $CH_2Cl_2$/methanol 20:1 v/v and 1% TEA was added), 0.12 g of DMT-Thymidine-HDA-Val-Ala-PAB-Chalcone (0.091 mmol) was obtained as a yellow solid (56.9% yield). $^1$H NMR (400 MHz, DMSO-*d6*) δ 9.96 (s, 1H), 8.15 (d, *J* = 7.0 Hz, 1H), 7.84 – 7.65 (m, 4H), 7.63 (d, *J* = 8.6 Hz, 3H), 7.48 – 7.35 (m, 7H), 7.33 – 7.21 (m, 7H), 7.04 (d, *J* = 8.5 Hz, 1H), 6.89 (dd, *J* = 8.9,

1.4 Hz, 4H), 6.17 (t, *J* = 6.4 Hz, 1H), 5.33 (d, *J* = 4.6 Hz, 1H), 5.12 (s, 2H), 4.39 (p, *J* = 7.0 Hz, 1H), 4.30 (td, *J* = 8.7, 4.4 Hz, 1H), 4.23 (t, *J* = 6.6 Hz, 2H), 4.17 (dd, *J* = 8.4, 7.0 Hz, 1H), 3.95 – 3.92 (m, 1H), 3.90 (s, 6H), 3.82 (s, 3H), 3.77 (s, 3H), 3.73 (s, 6H), 3.24 – 3.19 (m, 2H), 2.27 (ddd, *J* = 13.2, 6.2, 4.3 Hz, 1H), 2.21 – 2.09 (m, 3H), 2.04 – 1.92 (m, 1H), 1.70 – 1.61 (m, 2H), 1.53 (s, 3H), 1.48 (m, 2H), 1.34 (m, 2H), 1.30 (d, *J* = 7.1 Hz, 3H), 1.23 (m, 8H), 0.85 (dd, *J* = 14.5, 6.8 Hz, 6H).

*Synthesis of Val-Ala-Chalcone phosphoramidite*

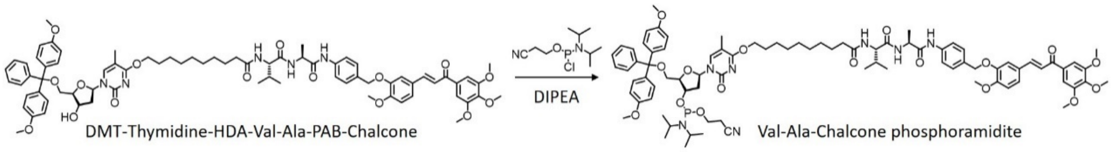


DMT-Thymidine-HDA-Val-Ala-PAB-Chalcone (110 mg, 0.084 mmol) was dissolved in 20 mL anhydrous dichloromethane under argon gas protection. The reaction was stirred in an ice bath, and 0.2 mL anhydrous DIPEA was added, then, 0.1 mL 2-cyanoethyl N,N-diisopropylchlorophosphoramidite was added. The reaction was monitored by thin-layer chromatography. When the reaction was completed, the mixture was washed by saturated $NaHCO_3$ and brine. The organic layer was collected and dried by anhydrous $Na_2SO_4$. After purification by silica gel column chromatography (elution solvents: EtOAc (1% TEA)), 70 mg of Val-Ala-Chalcone phosphoramidite was obtained as a yellow solid foam. $^1$H NMR (400 MHz, DMSO-*d*6) δ 9.92 (s, 1H), 8.11 (d, *J* = 7.0 Hz, 1H), 7.86 – 7.78 (m, 2H), 7.71 (dd, *J* = 35.1, 15.6 Hz, 2H), 7.63 – 7.56 (m, 3H), 7.46 – 7.33 (m, 7H), 7.32 – 7.20 (m, 7H), 7.03 (d, *J* = 8.5 Hz, 1H), 6.87 (ddd, *J* = 8.8, 5.8, 2.6 Hz, 4H), 6.17 (dt, *J* = 9.6, 6.4 Hz, 1H), 5.11 (s, 2H), 4.51 (dt, *J* = 10.4, 4.7 Hz, 1H), 4.38 (p, *J* = 6.9 Hz, 1H), 4.22 (t, *J* = 6.6 Hz, 2H), 4.16 (dd, *J* = 8.5, 6.9 Hz, 1H), 4.06 (dd, *J* = 23.1, 3.7 Hz, 1H), 3.89 (s, 6H), 3.81 (s, 3H), 3.76 (s, 3H), 3.72 (d, *J* = 2.1 Hz, 8H), 3.61 – 3.49 (m, 2H), 3.27 – 3.22 (m, 2H), 2.74 (t, *J* = 5.9 Hz, 1H), 2.62 (t, *J* = 5.9 Hz, 1H), 2.33 – 2.24 (m, 1H), 2.22 – 2.07 (m, 2H), 1.97 (m, 1H), 1.69 – 1.60 (m, 2H), 1.55 (d, *J* = 10.1 Hz, 3H), 1.50 – 1.44 (m, 2H), 1.34 (d, *J* = 4.7 Hz, 2H), 1.29 (d, *J* = 7.1 Hz, 3H), 1.23 (s, 8H), 1.14 – 1.05 (m, 9H), 0.97 (d, *J* = 6.8 Hz, 3H), 0.84 (dd, *J* = 14.1, 6.8 Hz, 6H). $^{31}$P NMR (162 MHz, DMSO-*d*6) δ 147.59, 147.27.

## 6. NMR spectra

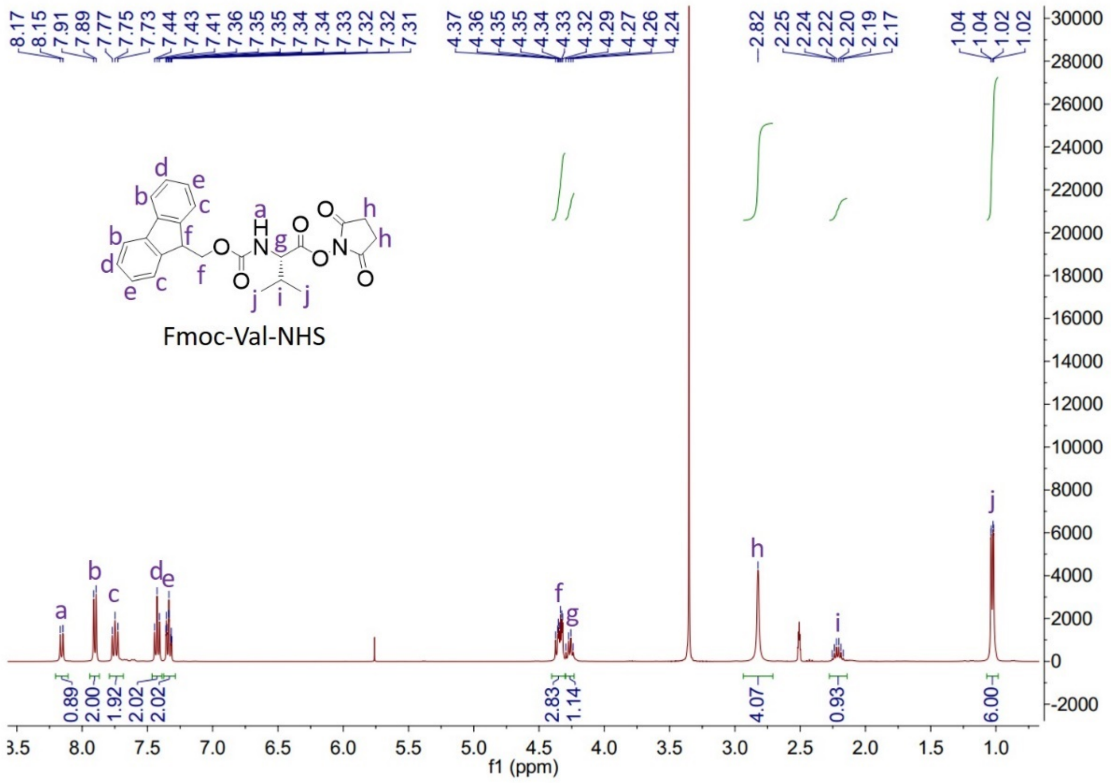


**Figure S35.** $^{1}$H NMR spectrum of Fmoc-Val-NHS.

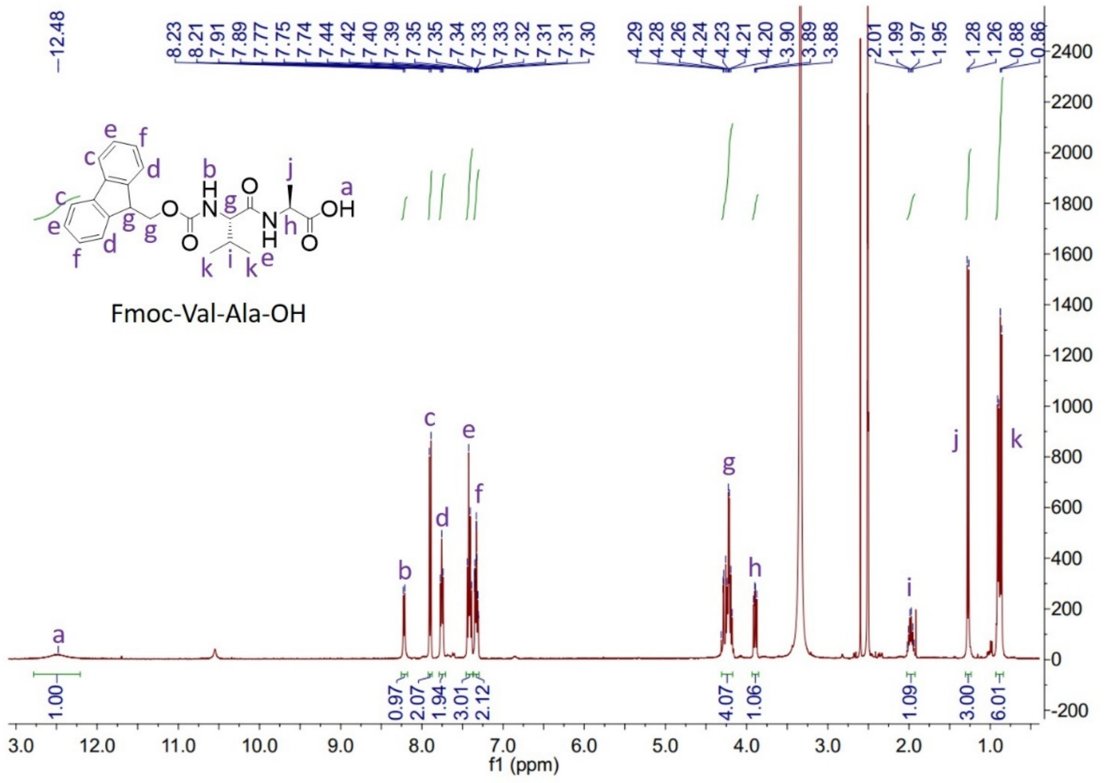


**Figure S36.** $^{1}$H NMR spectrum of Fmoc-Val-Ala-OH.

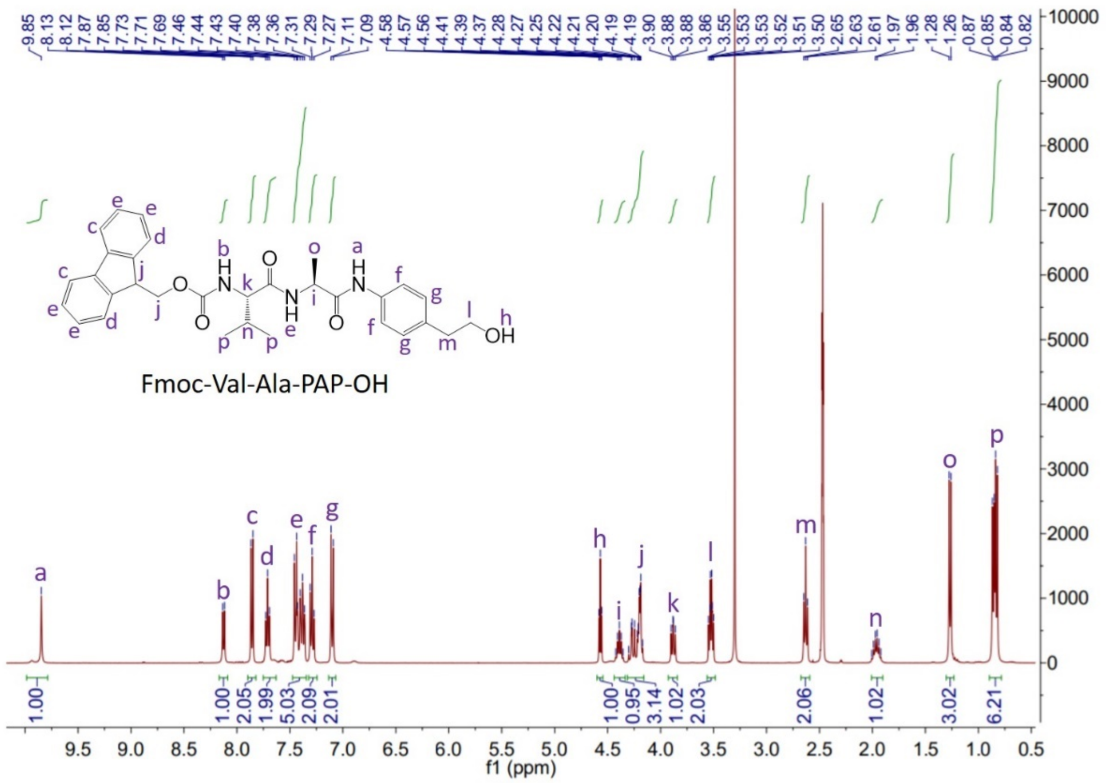


**Figure S37.** $^{1}H$ NMR spectrum of Fmoc-Val-Ala-PAP-OH.

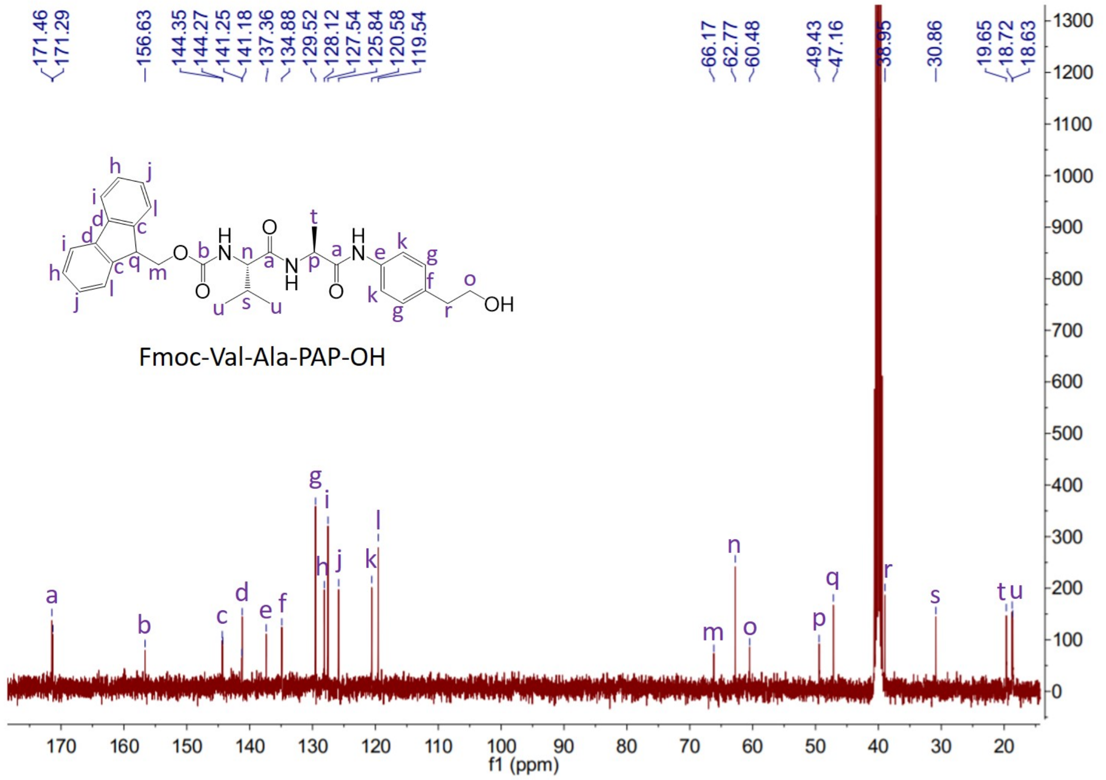


**Figure S38.** $^{13}C$ NMR spectrum of Fmoc-Val-Ala-PAP-OH.

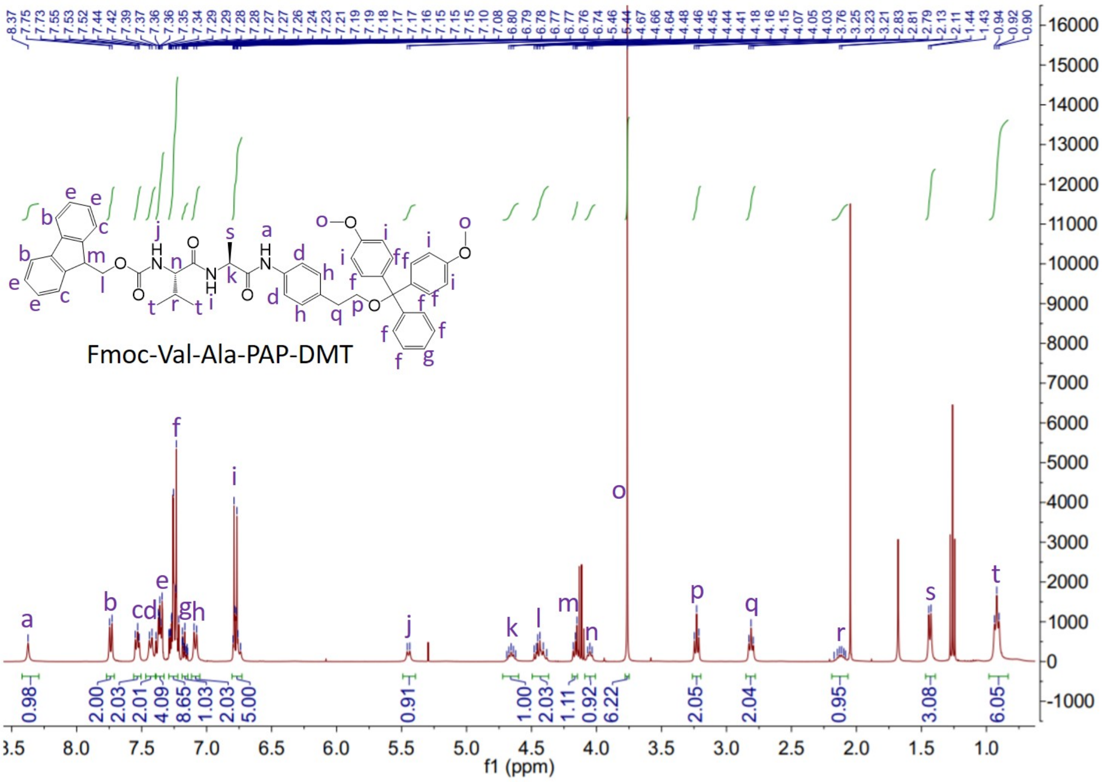


**Figure S39.** $^{1}$H NMR spectrum of Fmoc-Val-Ala-PAP-DMT.

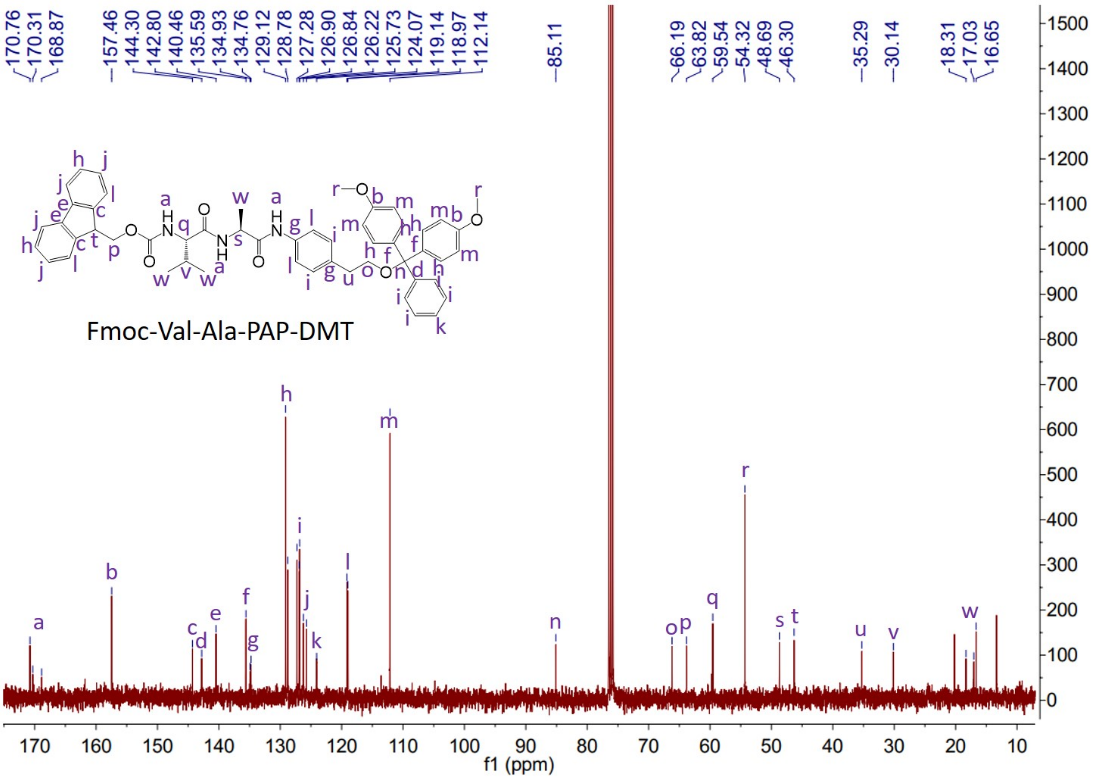


**Figure S40.** $^{13}$C NMR spectrum of Fmoc-Val-Ala-PAP-DMT.

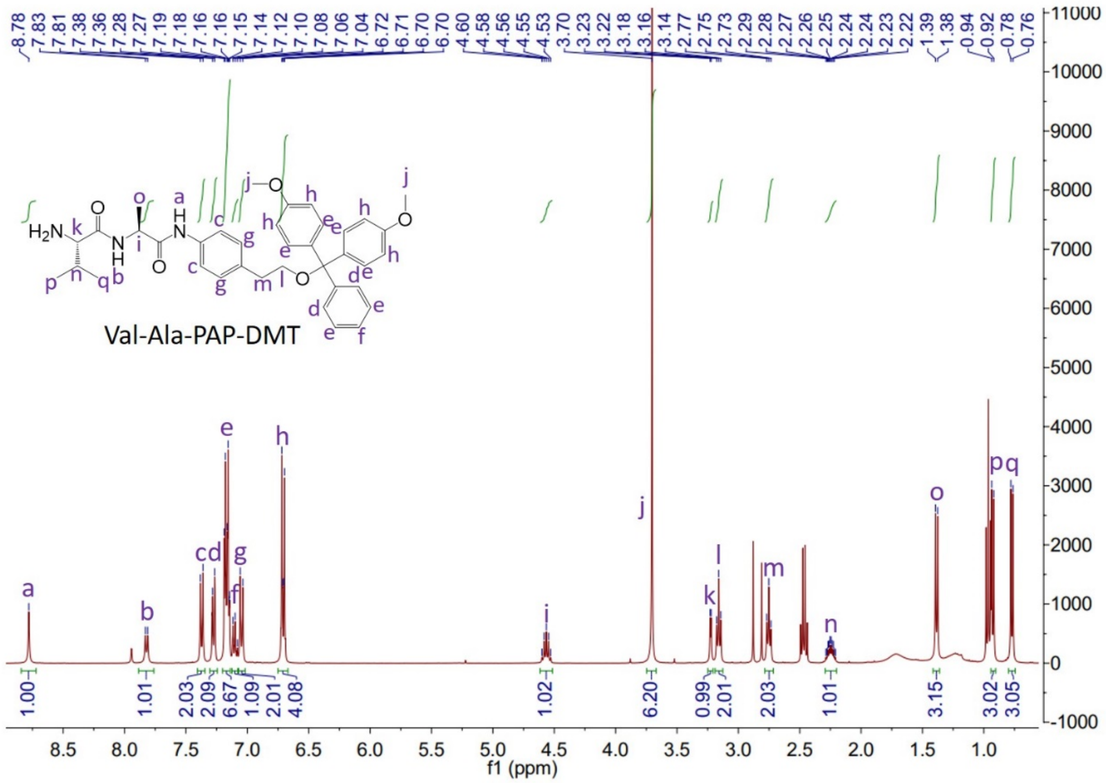


**Figure S41.** $^{1}$H NMR spectrum of Val-Ala-PAP-DMT.

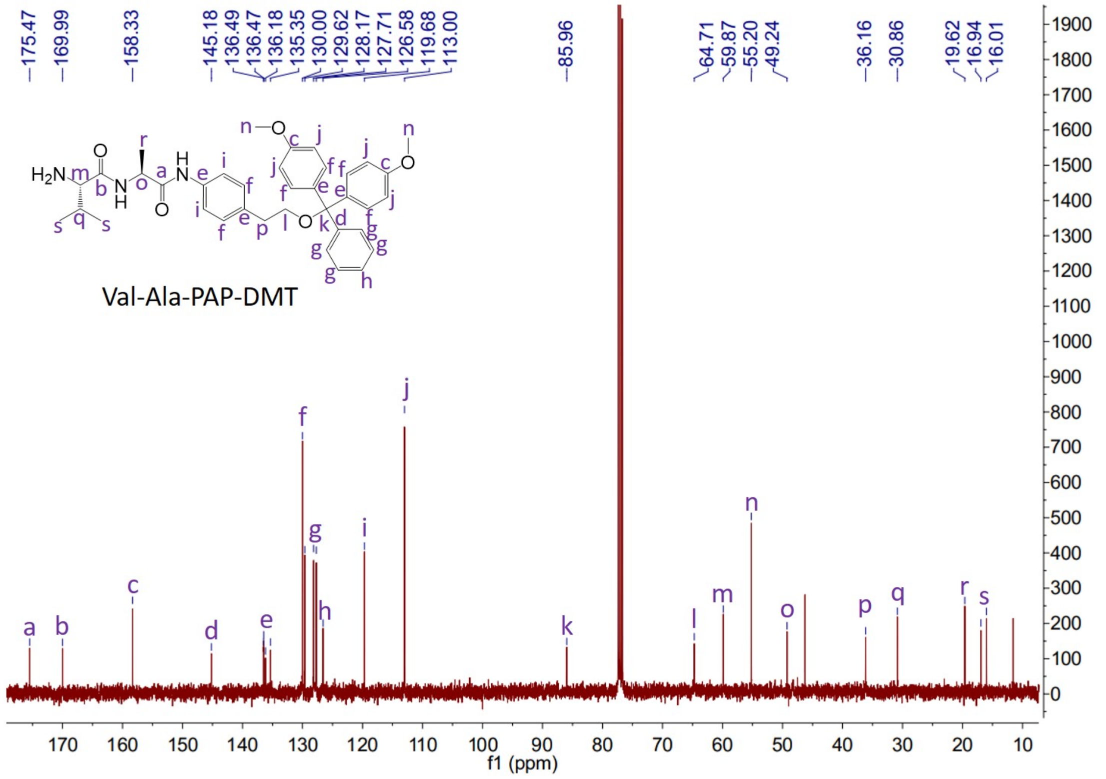


**Figure S42.** $^{13}$C NMR spectrum of Val-Ala-PAP-DMT.

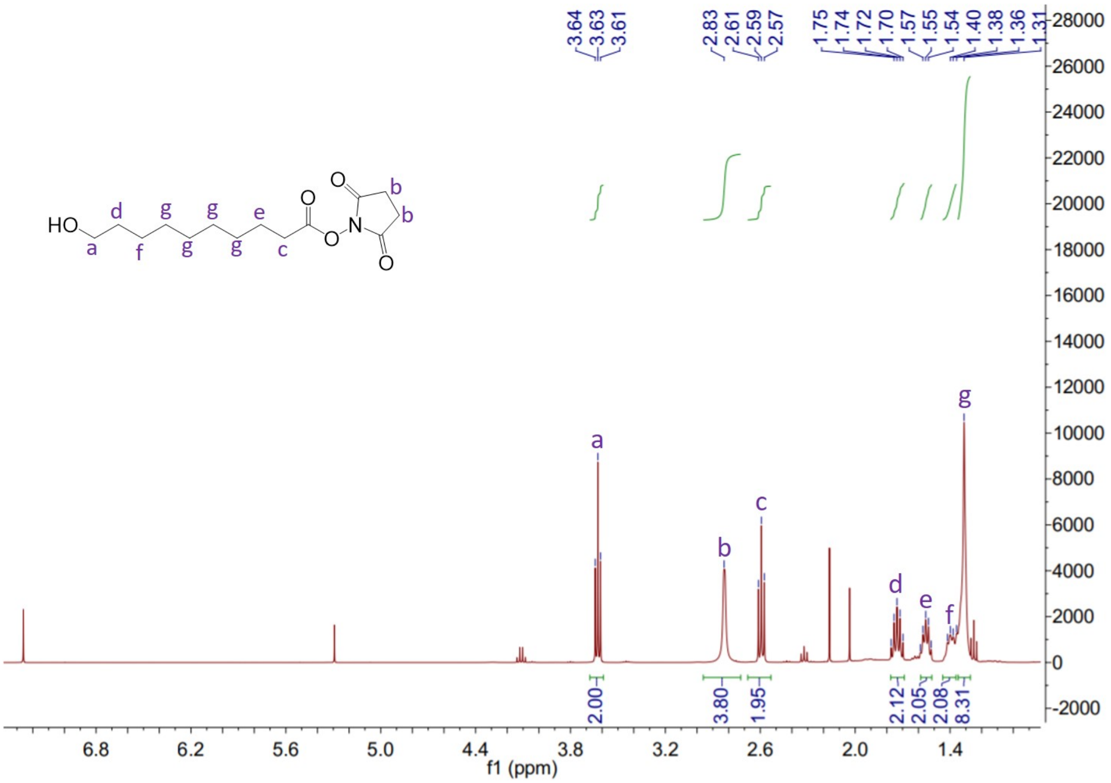


**Figure S43.** $^{1}H$ NMR spectrum of HDA-NHS.

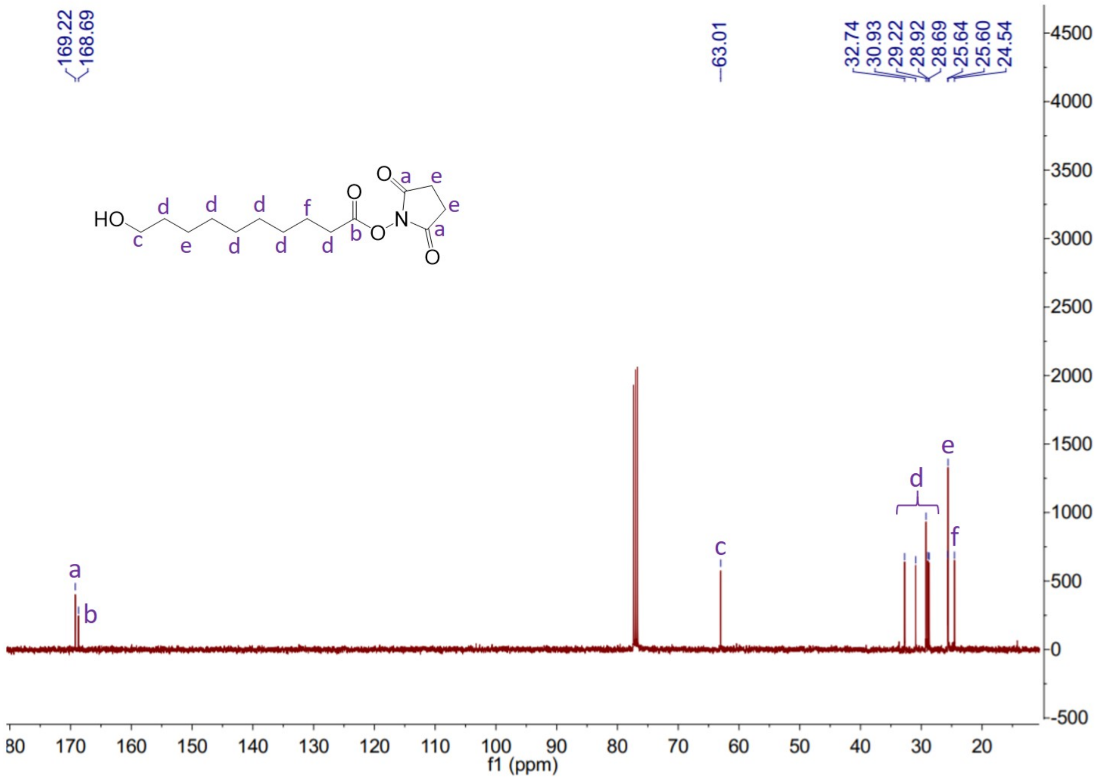


**Figure S44.** $^{13}C$ NMR spectrum of HDA-NHS.

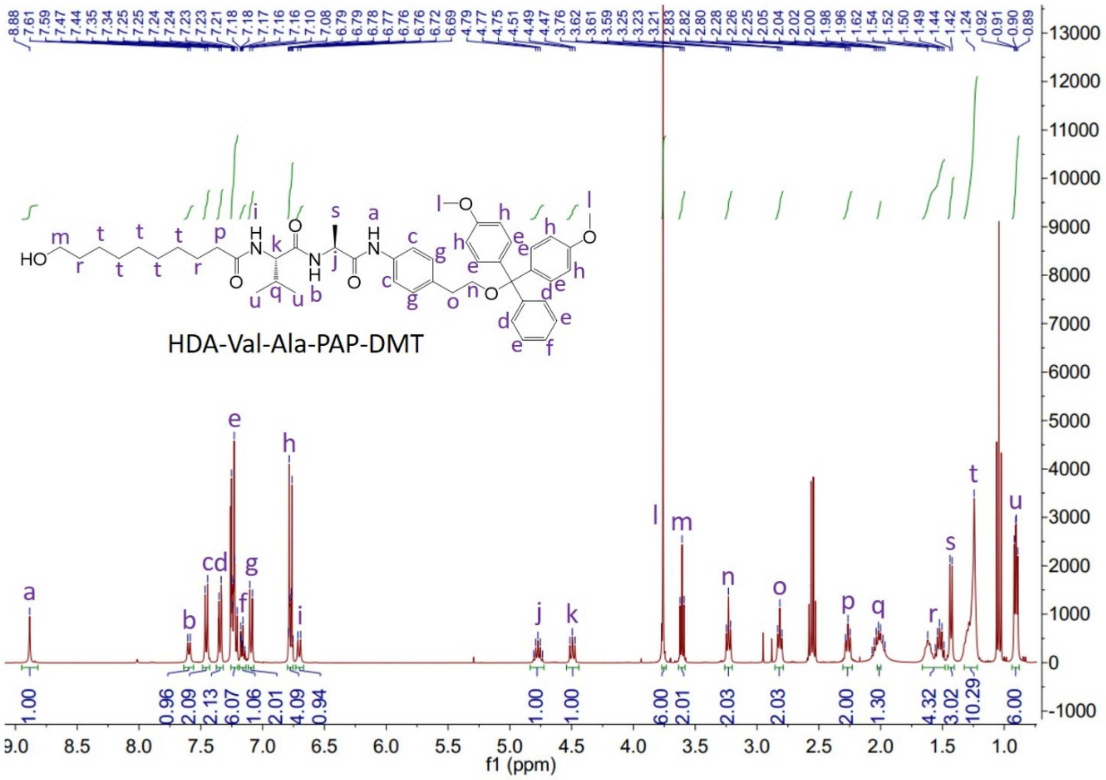

**Figure S45.** $^{1}H$ NMR spectrum of HDA-Val-Ala-PAP-DMT.

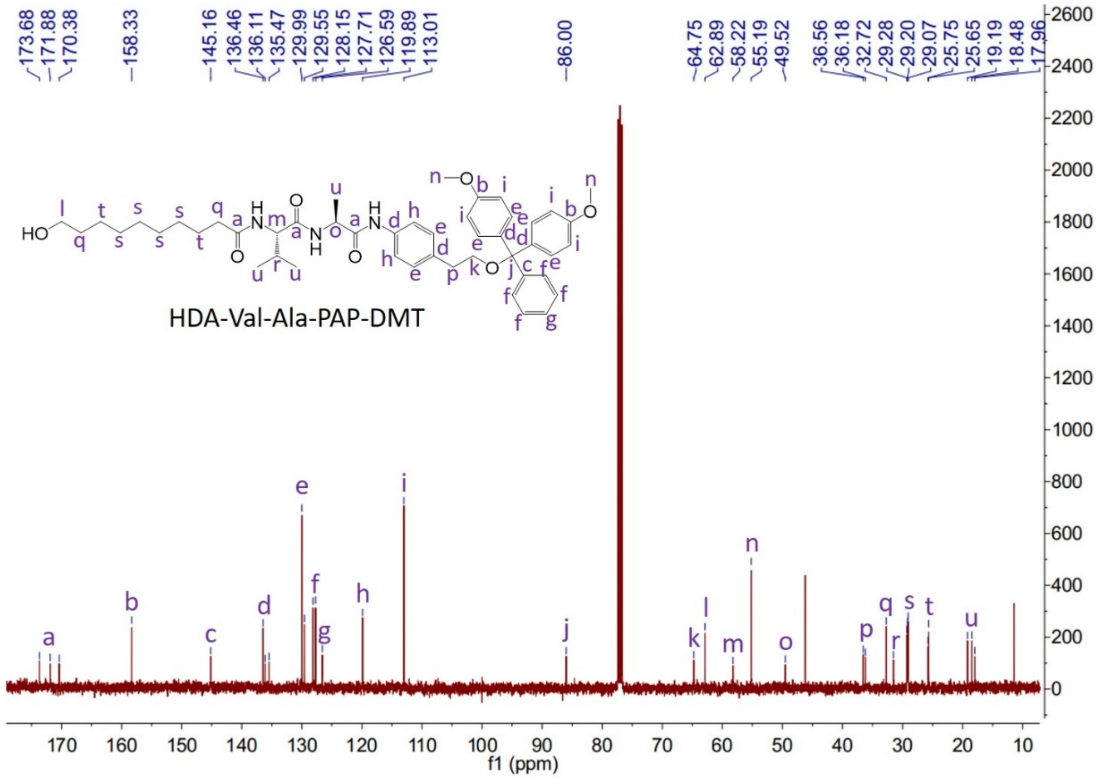

**Figure S46.** $^{13}C$ NMR spectrum of HDA-Val-Ala-PAP-DMT.

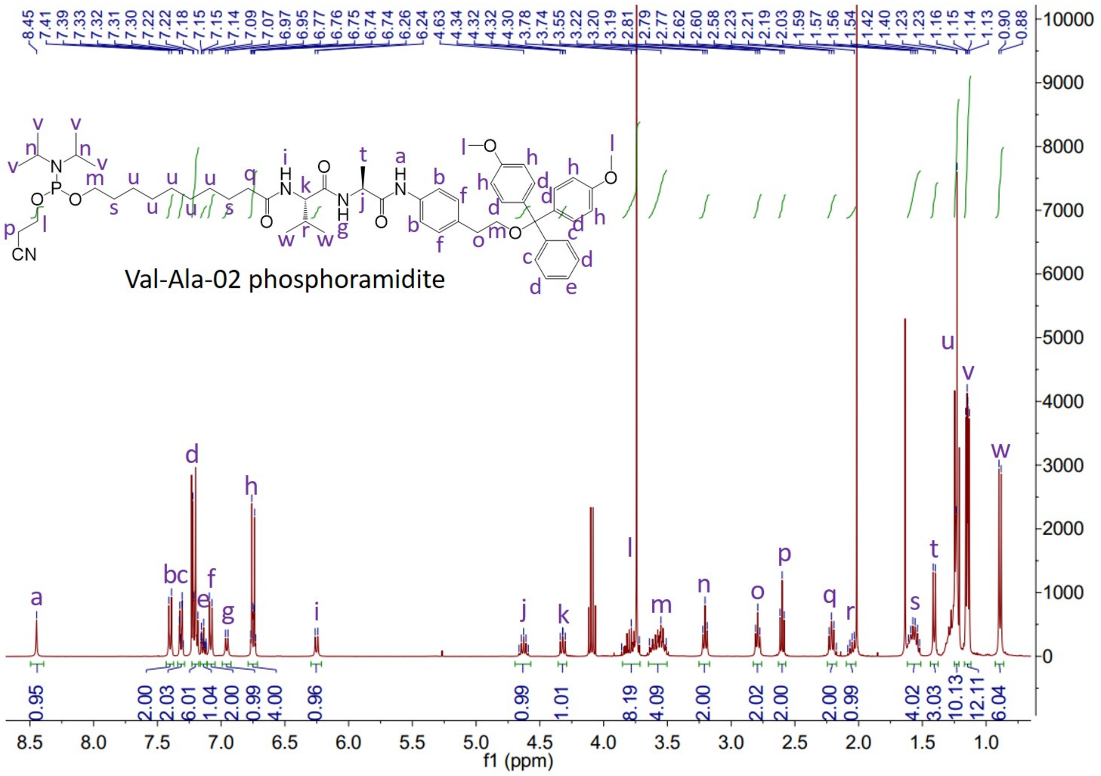


**Figure S47.** $^{1}H$ NMR spectrum of Val-Ala-02 phosphoramidite.

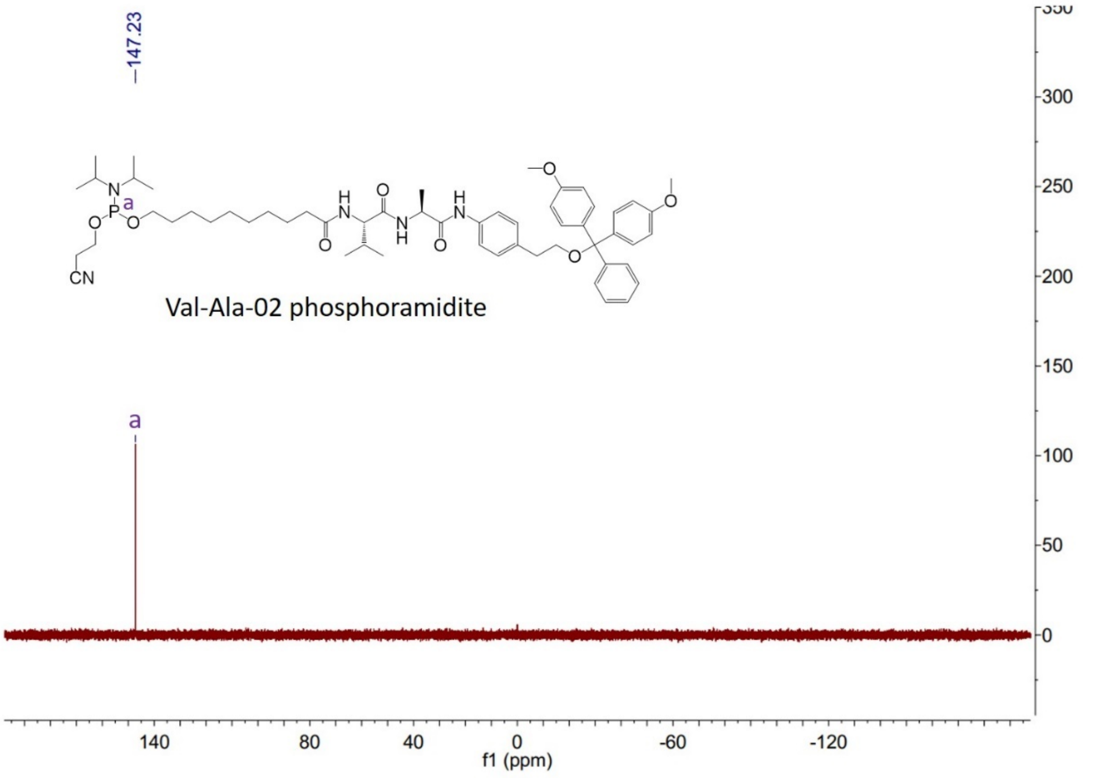


**Figure S48.** $^{31}P$ NMR spectrum of Val-Ala-02 phosphoramidite.

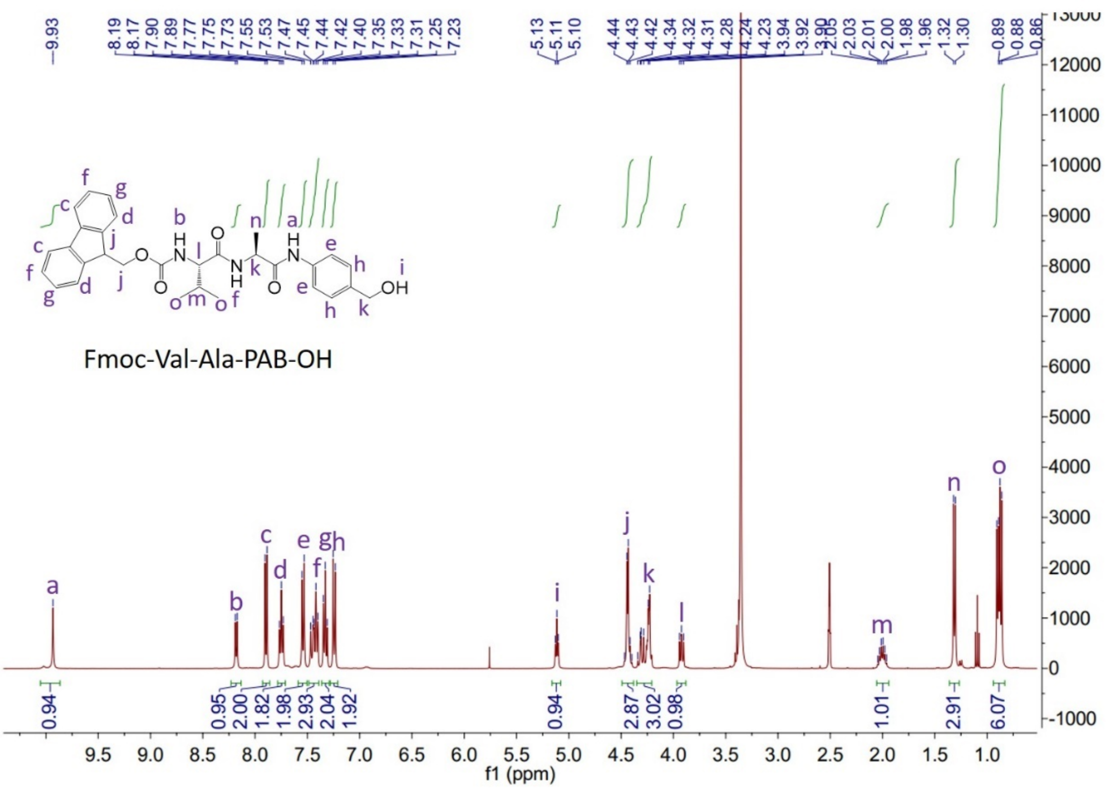


**Figure S49.** $^{1}H$ NMR spectrum of Fmoc-Val-Ala-PAB-OH.

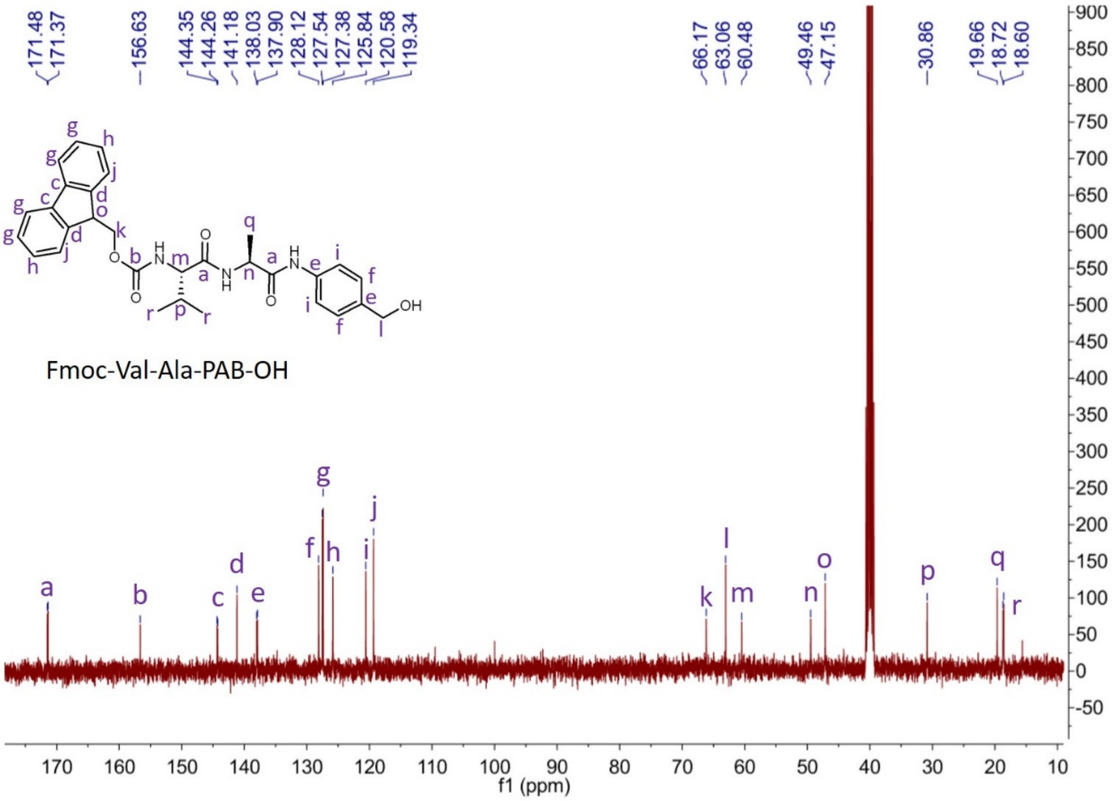


**Figure S50.** $^{13}C$ NMR spectrum of Fmoc-Val-Ala-PAB-OH.

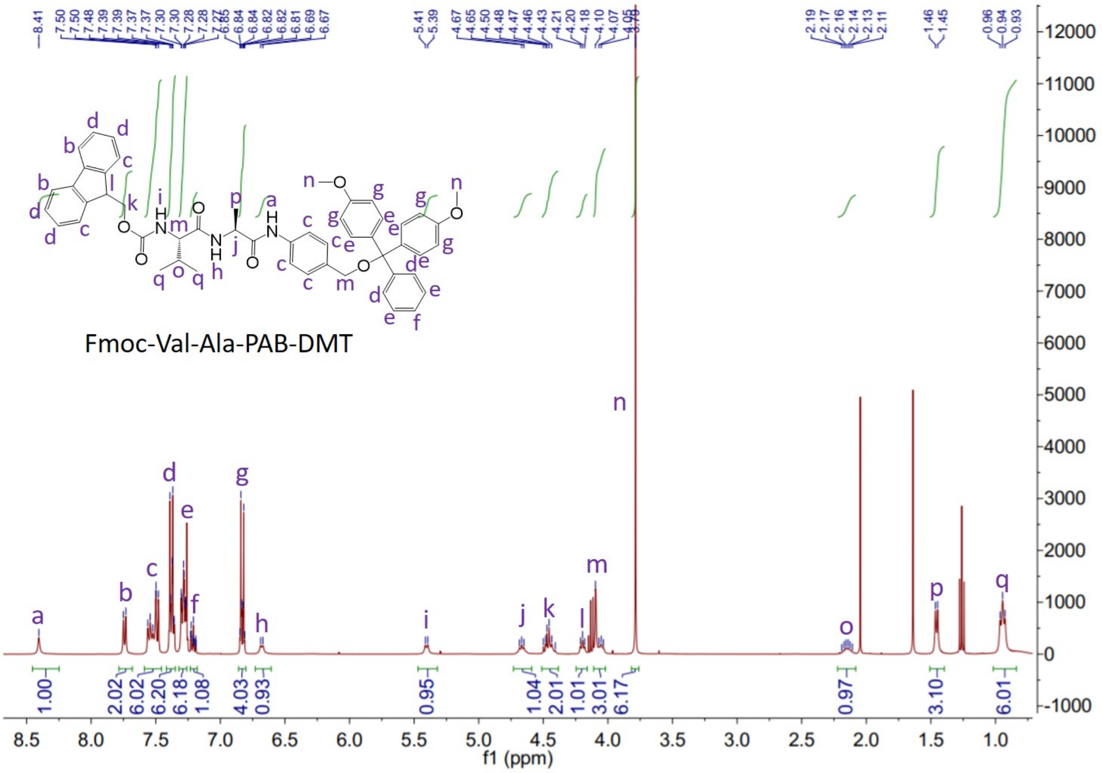

**Figure S51.** $^{1}H$ NMR spectrum of Fmoc-Val-Ala-PAB-DMT

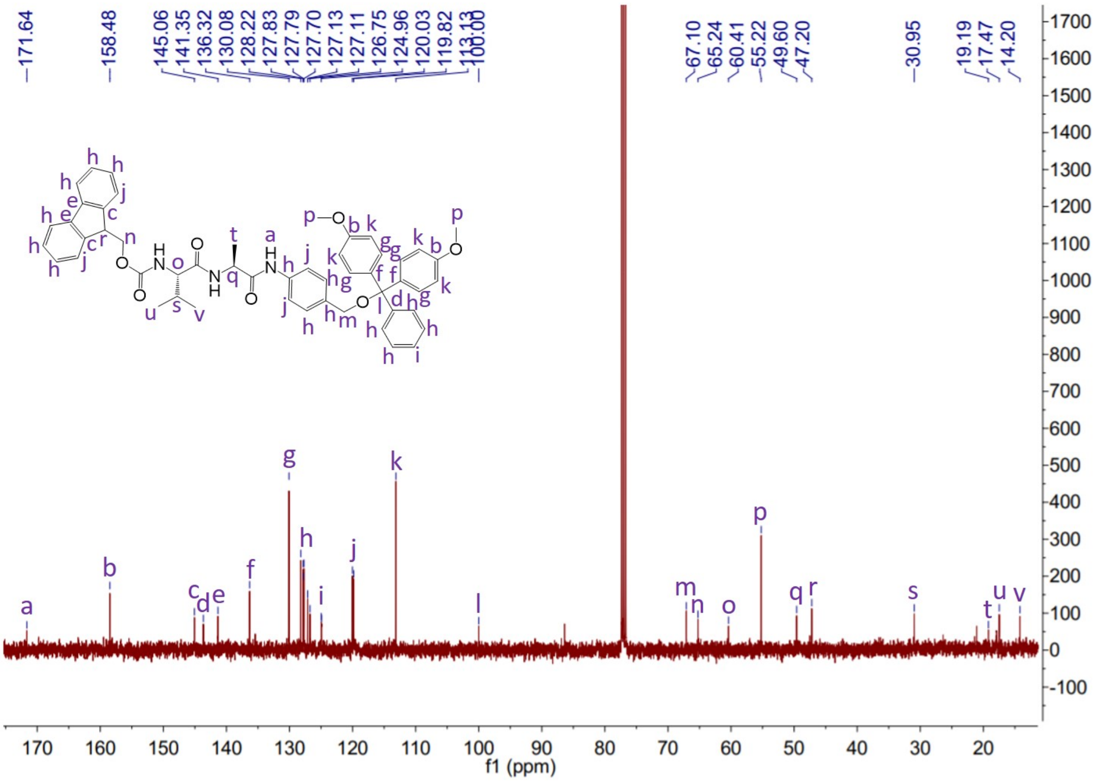

**Figure S52.** $^{13}C$ NMR spectrum of Fmoc-Val-Ala-PAB-DMT

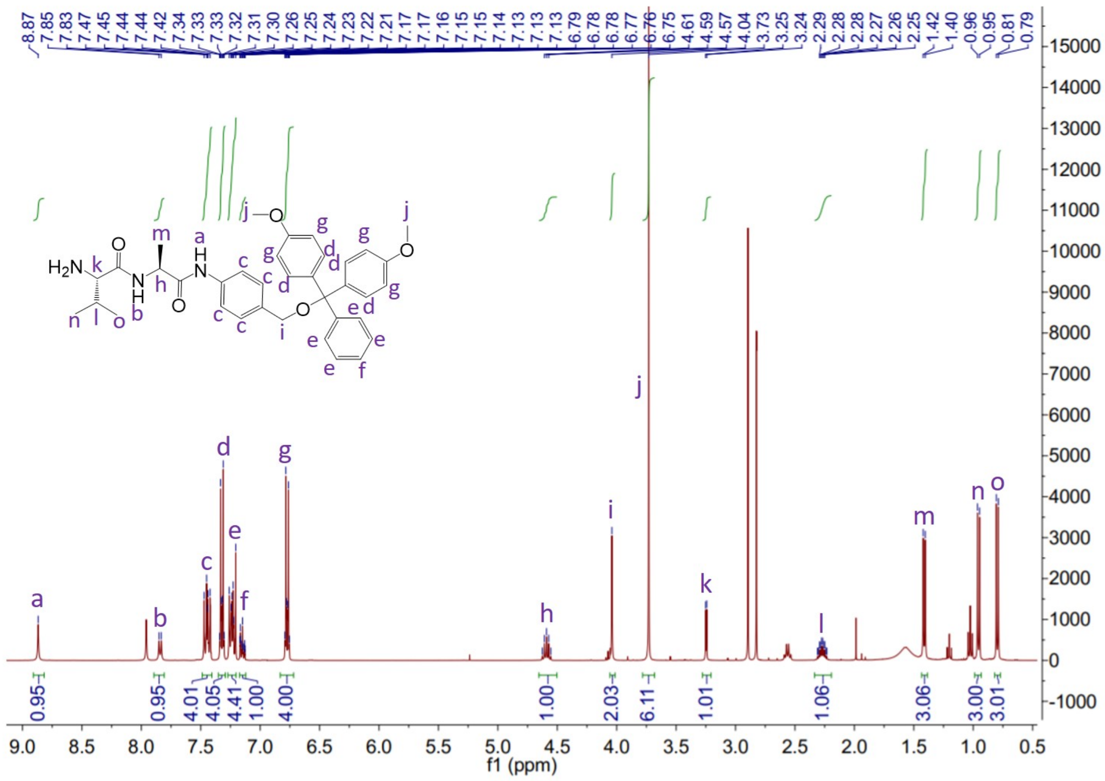

**Figure S53.** $^{1}$H NMR spectrum of Val-Ala-PAB-DMT

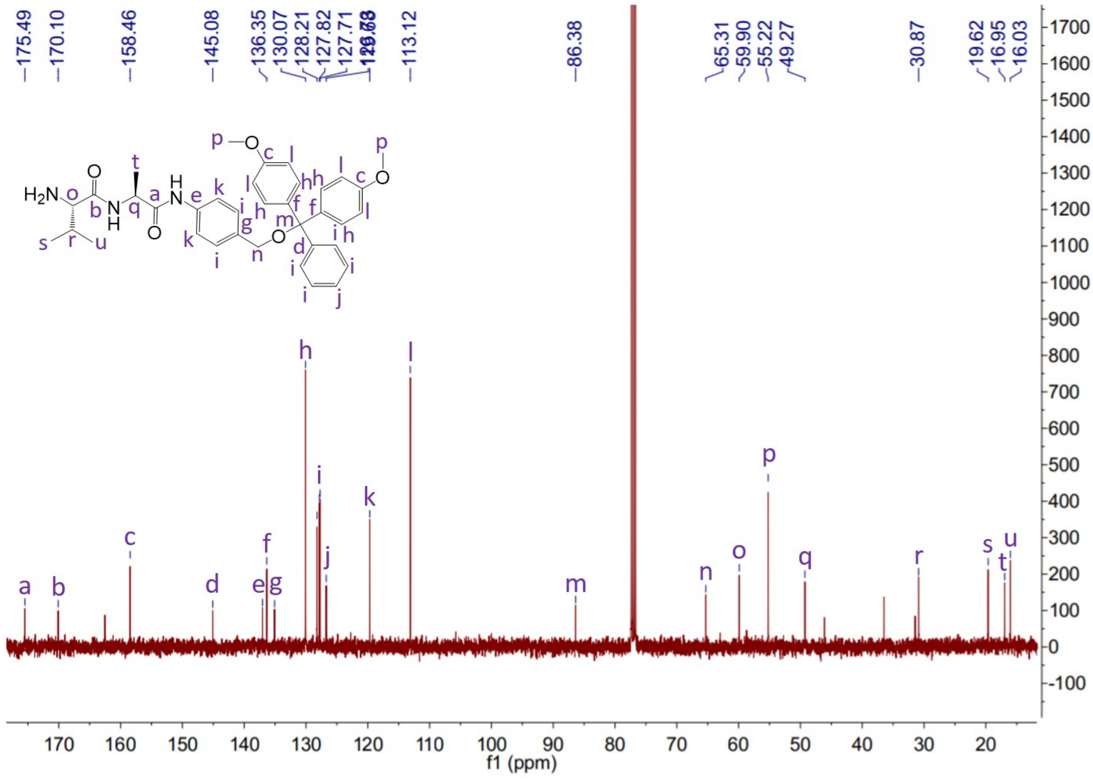

**Figure S54.** $^{13}$C NMR spectrum of Val-Ala-PAB-DMT.

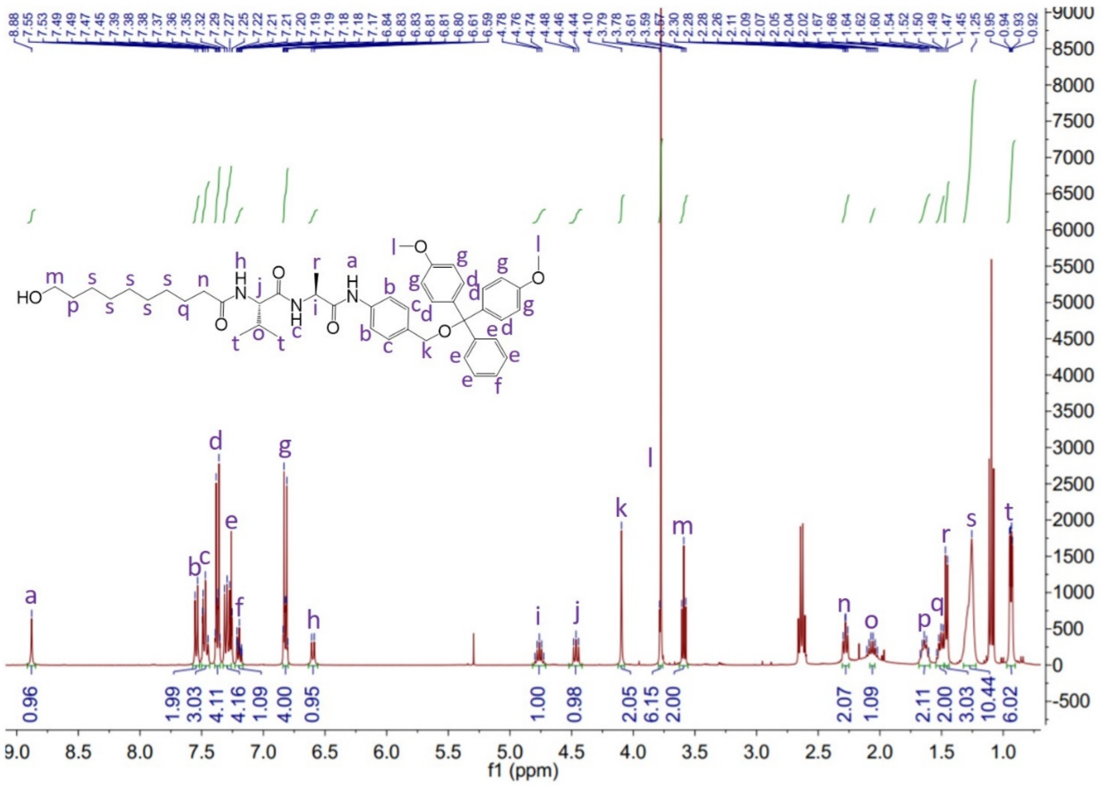

**Figure S55.** $^{1}$H NMR spectrum of HDA-Val-Ala-PAB-DMT

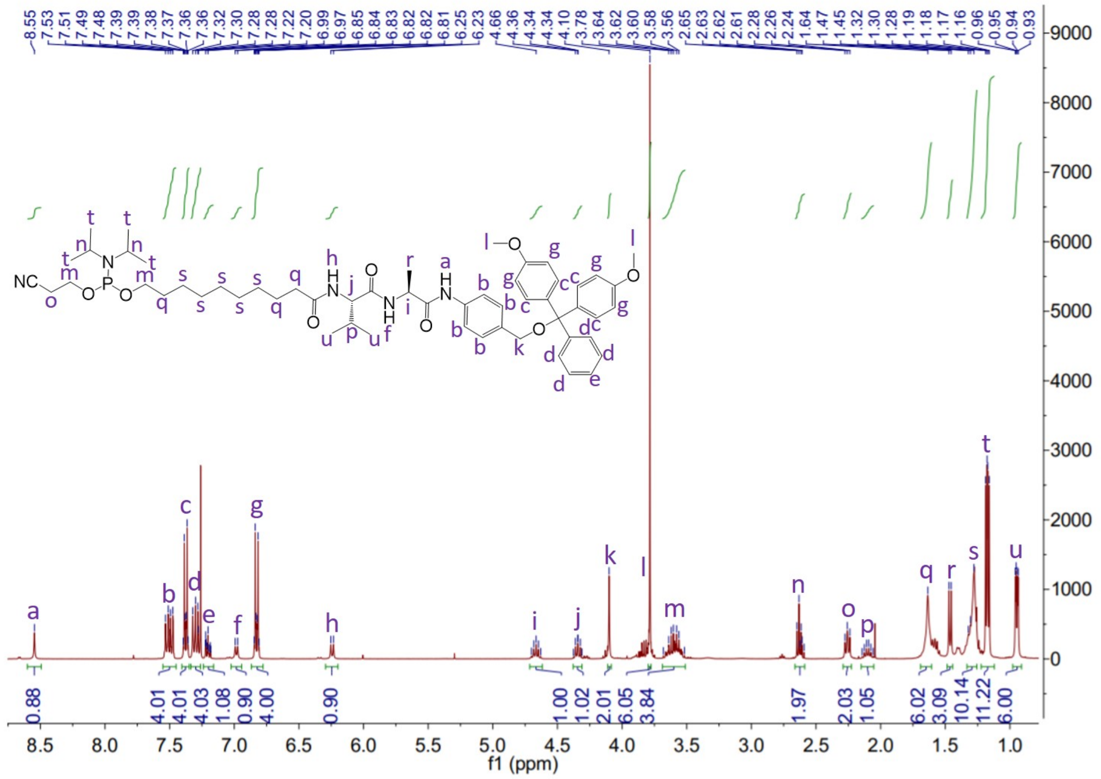

**Figure S56.** $^{1}$H NMR spectrum of Val-Ala-01 phosphoramidite.

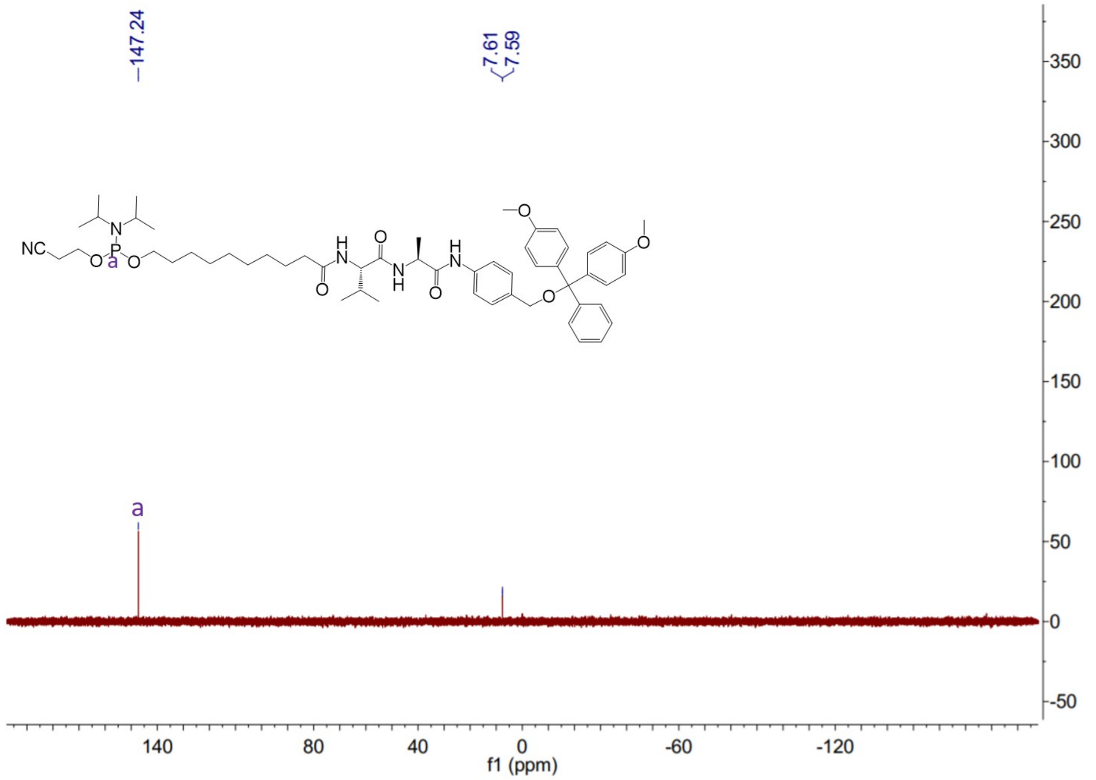


**Figure S57.** $^{31}$P NMR spectrum of Val-Ala-01 phosphoramidite.

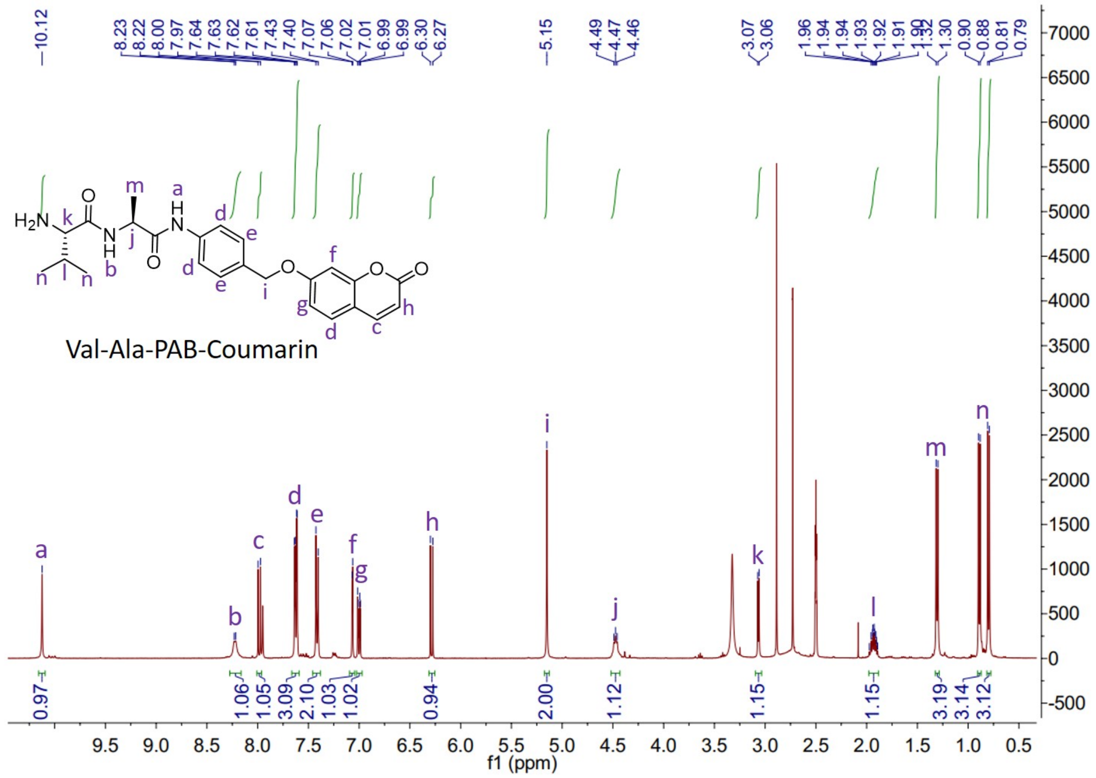


**Figure S58.** $^{1}$H NMR spectrum of Val-Ala-PAB-Coumarin.

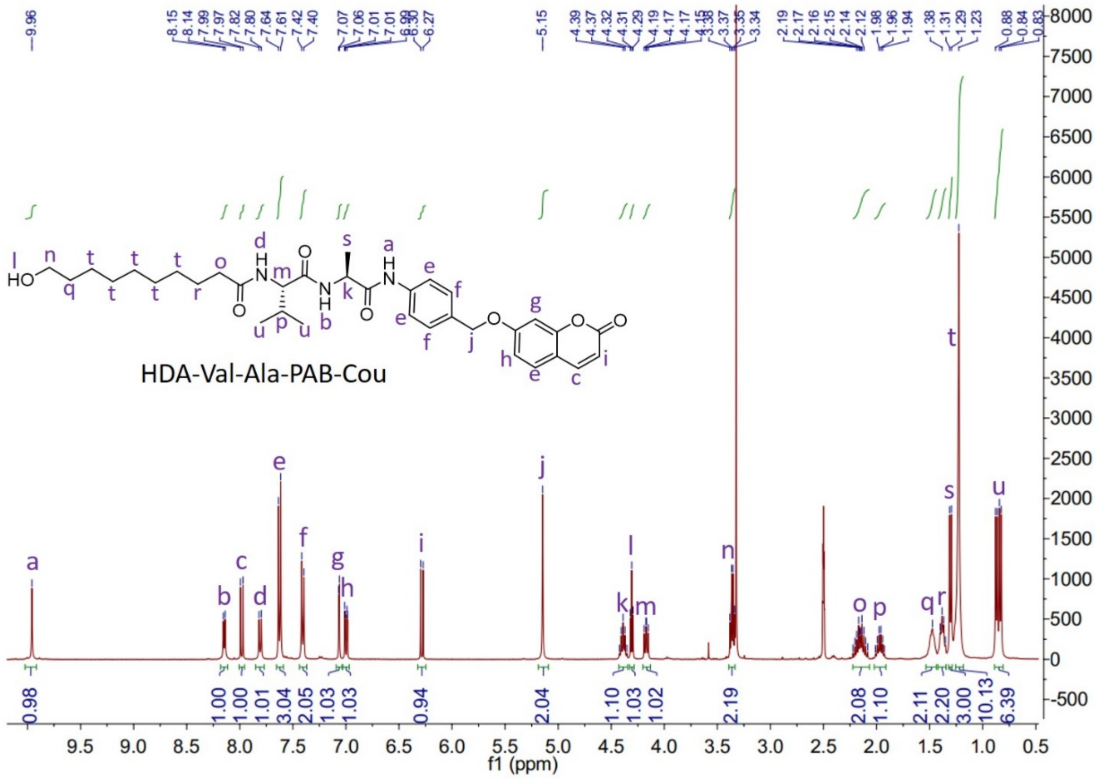


**Figure S59.** $^1$H NMR spectrum of HDA-Val-Ala-PAB-Coumarin.

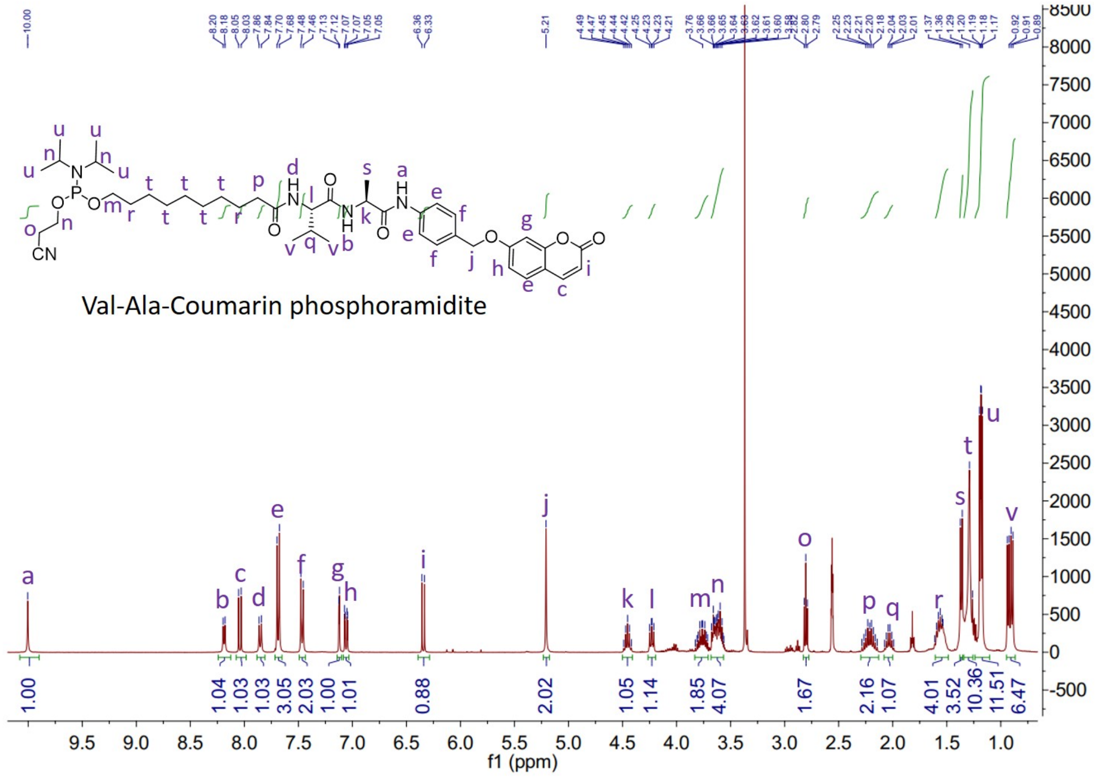


**Figure S60.** $^1$H NMR spectrum of Val-Ala-Coumarin phosphoramidite.

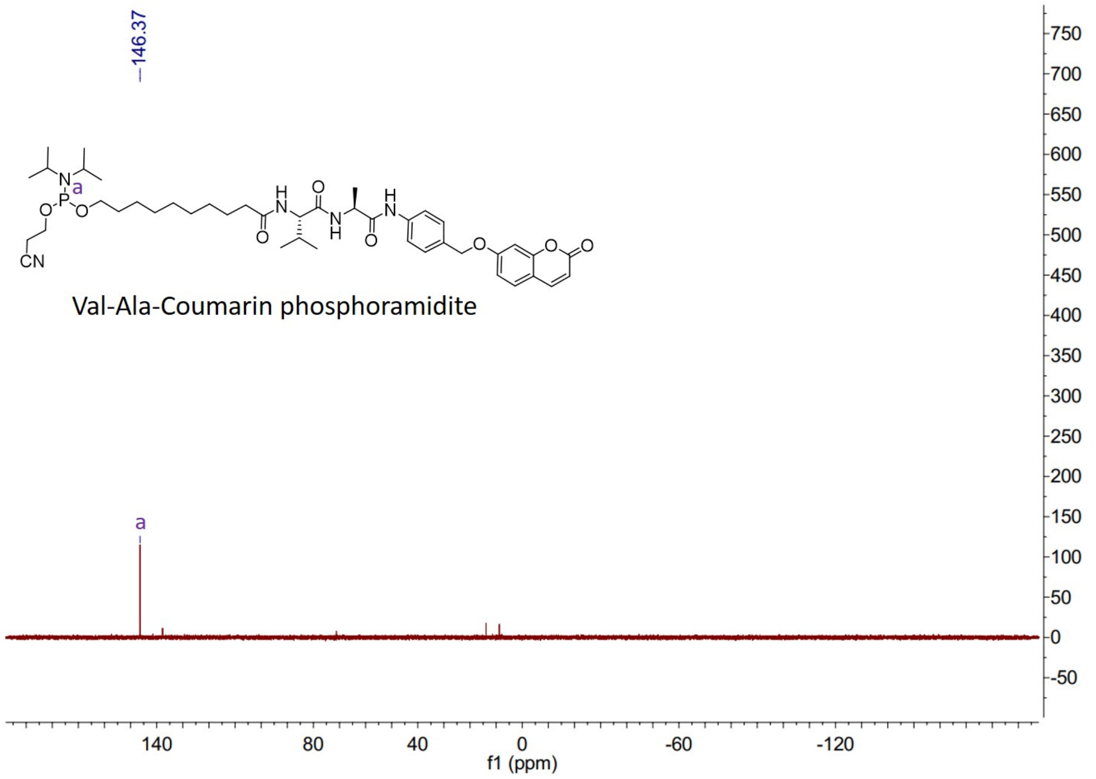


**Figure S61.** $^{31}P$ NMR spectrum of Val-Ala-Coumarin phosphoramidite.

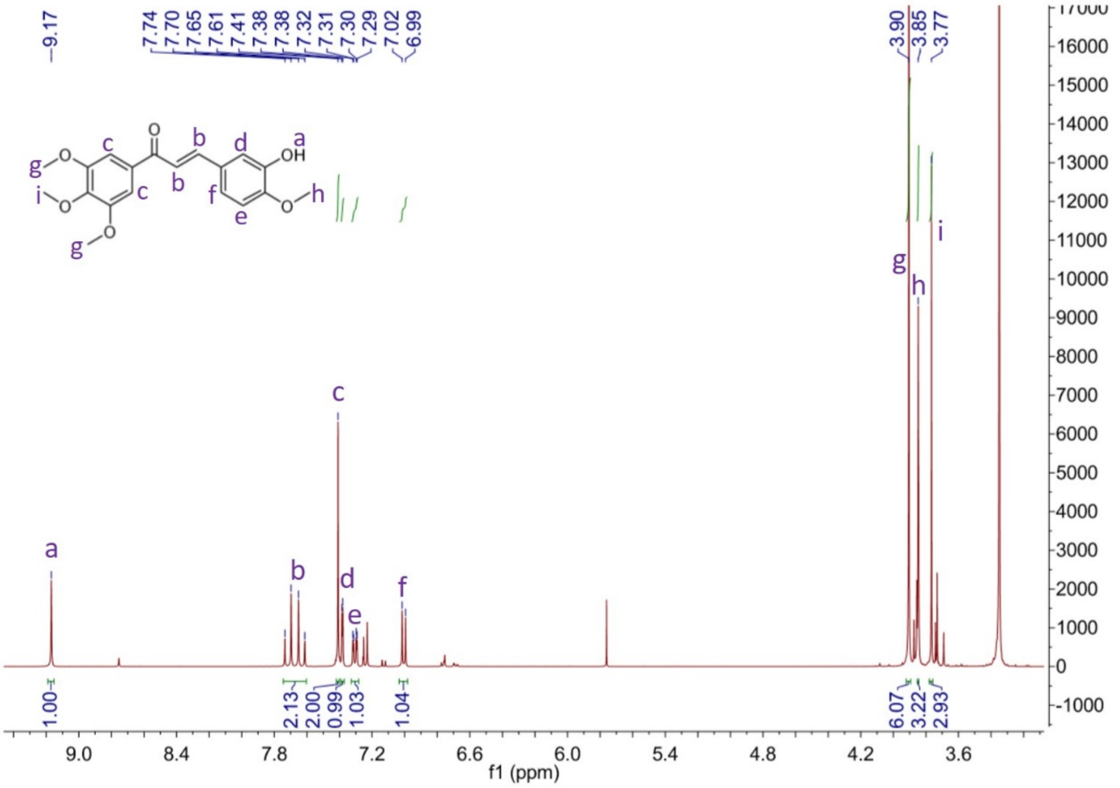


**Figure S62.** $^{1}H$ NMR spectrum of chalcone.

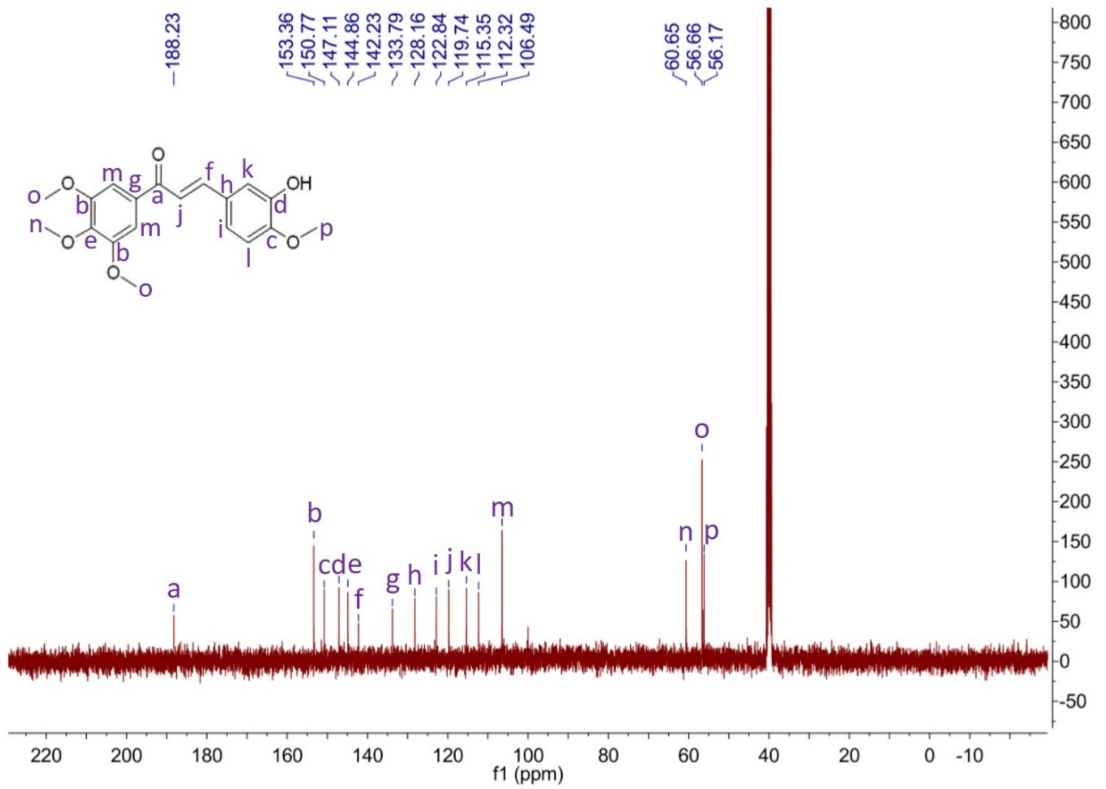

**Figure S63.** $^{13}C$ NMR spectrum of chalcone.

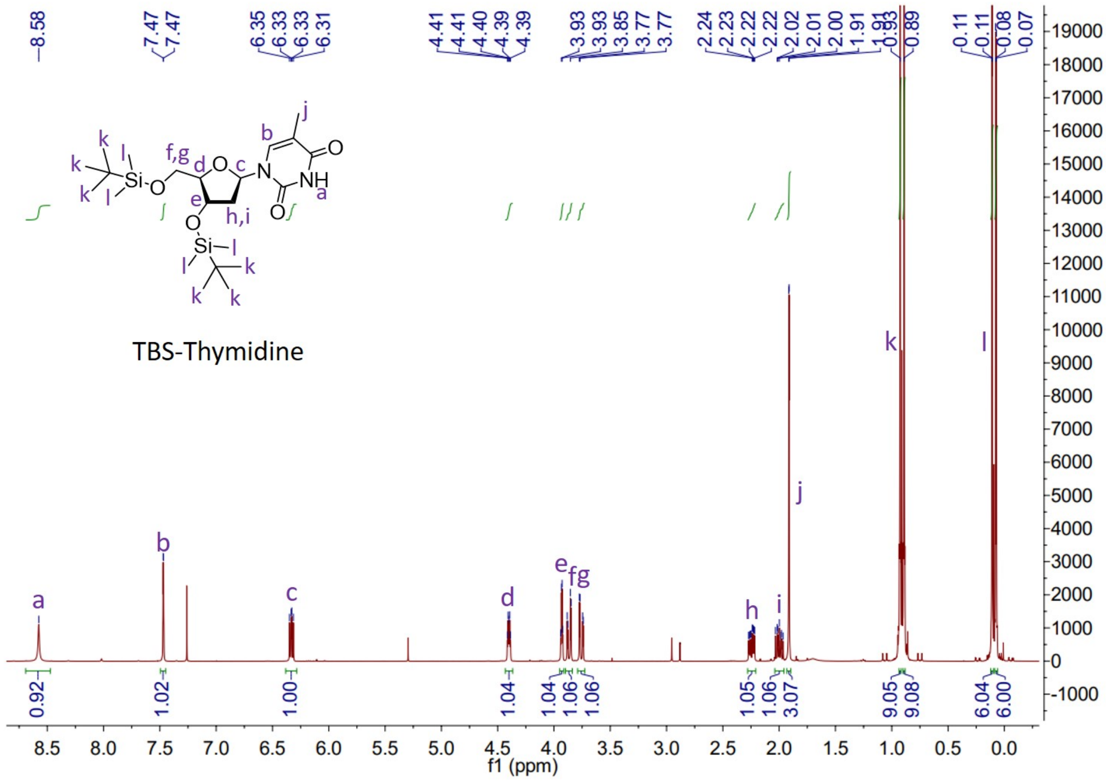

**Figure S64.** $^{1}H$ NMR spectrum of TBS-Thymidine.

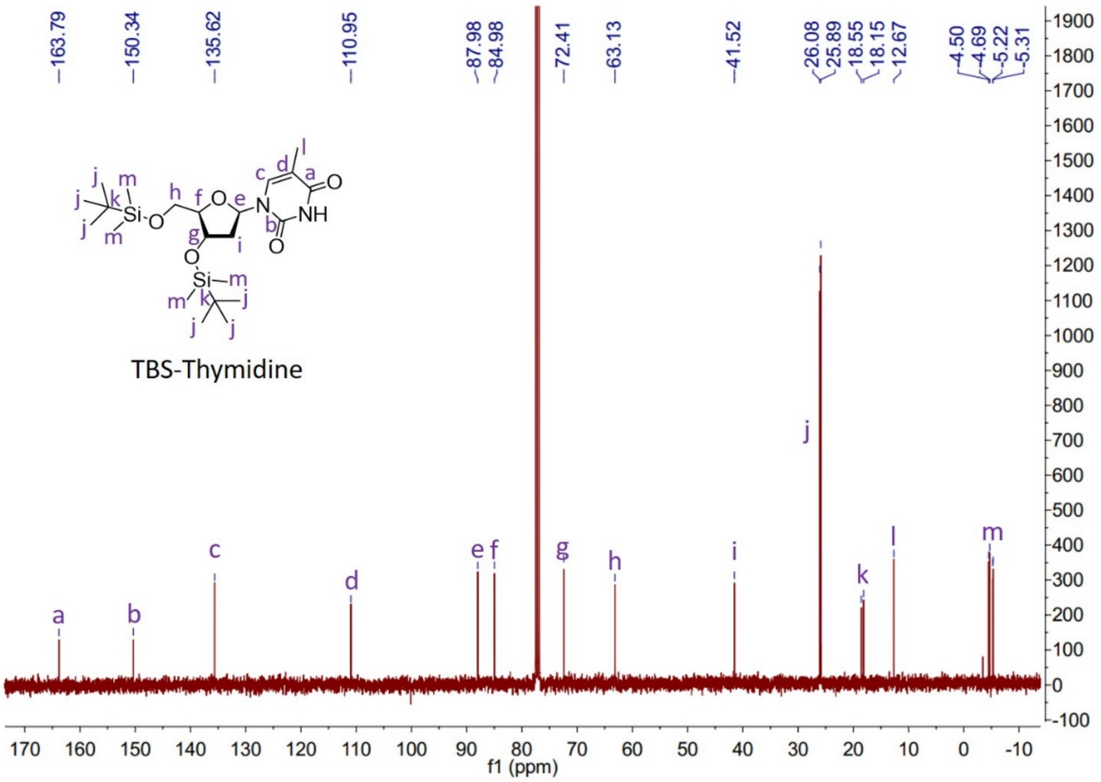


**Figure S65.** $^{13}C$ NMR spectrum of TBS-Thymidine.

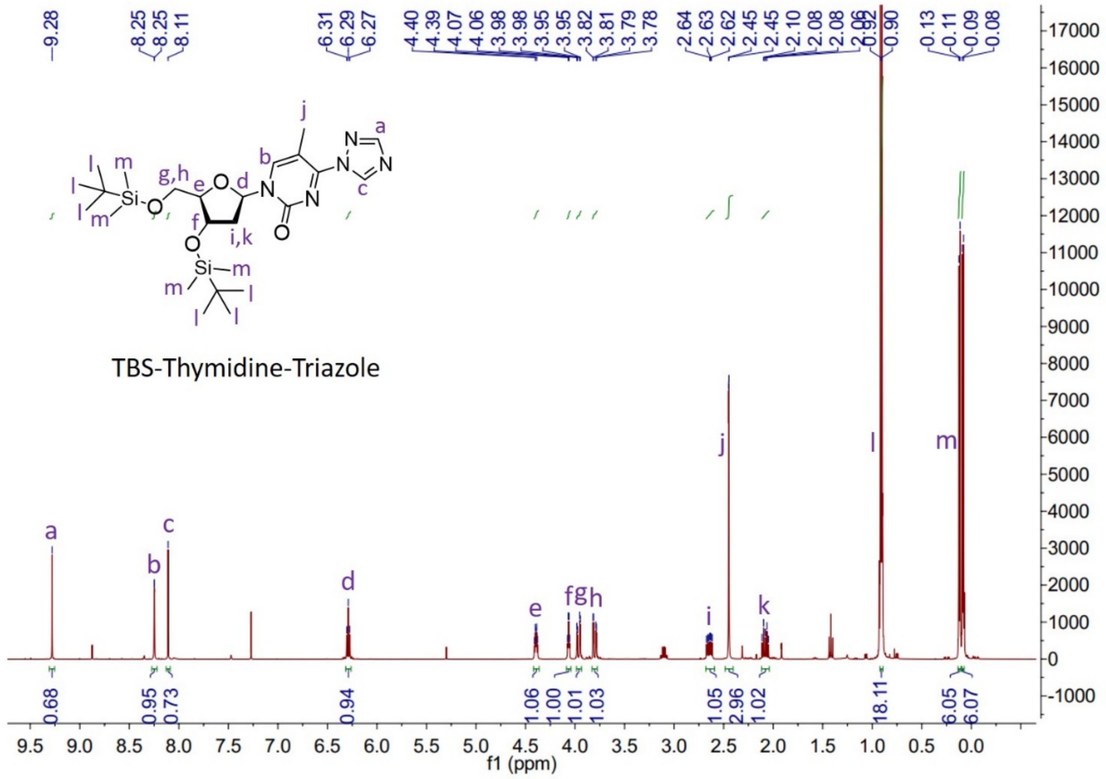


**Figure S66.** $^{1}H$ NMR spectrum of TBS-Thymidine-Triazole.

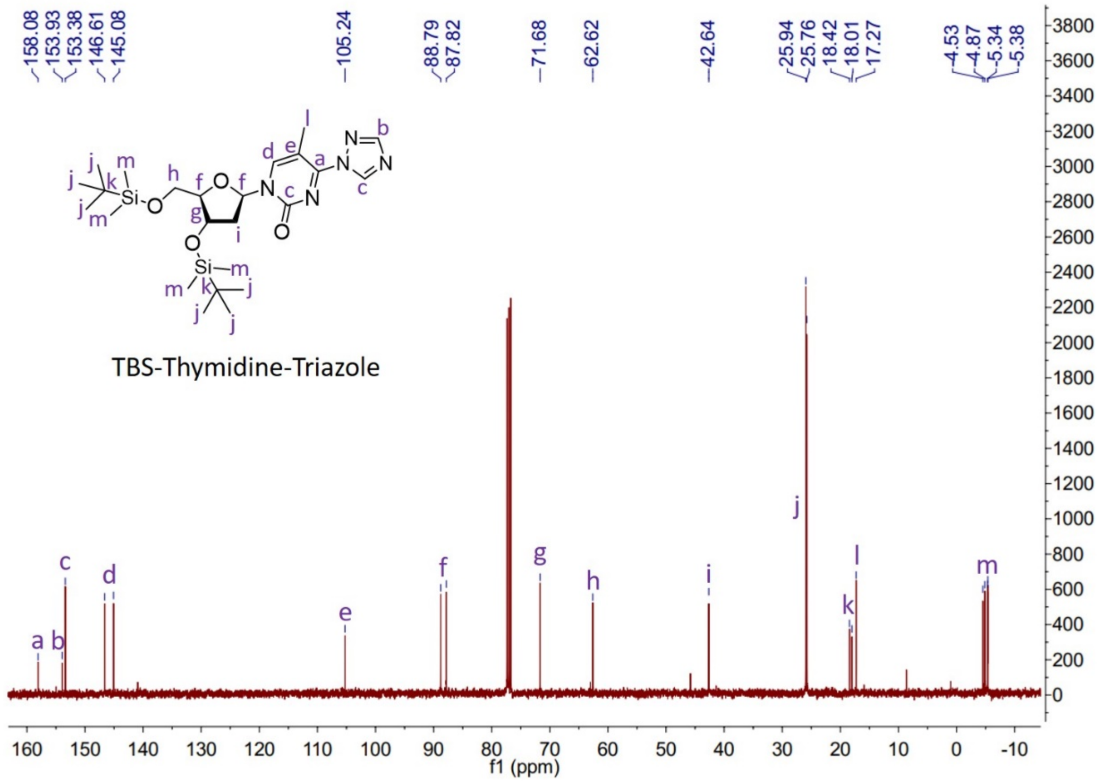

**Figure S67.** $^{13}C$ NMR spectrum of TBS-Thymidine-Triazole.

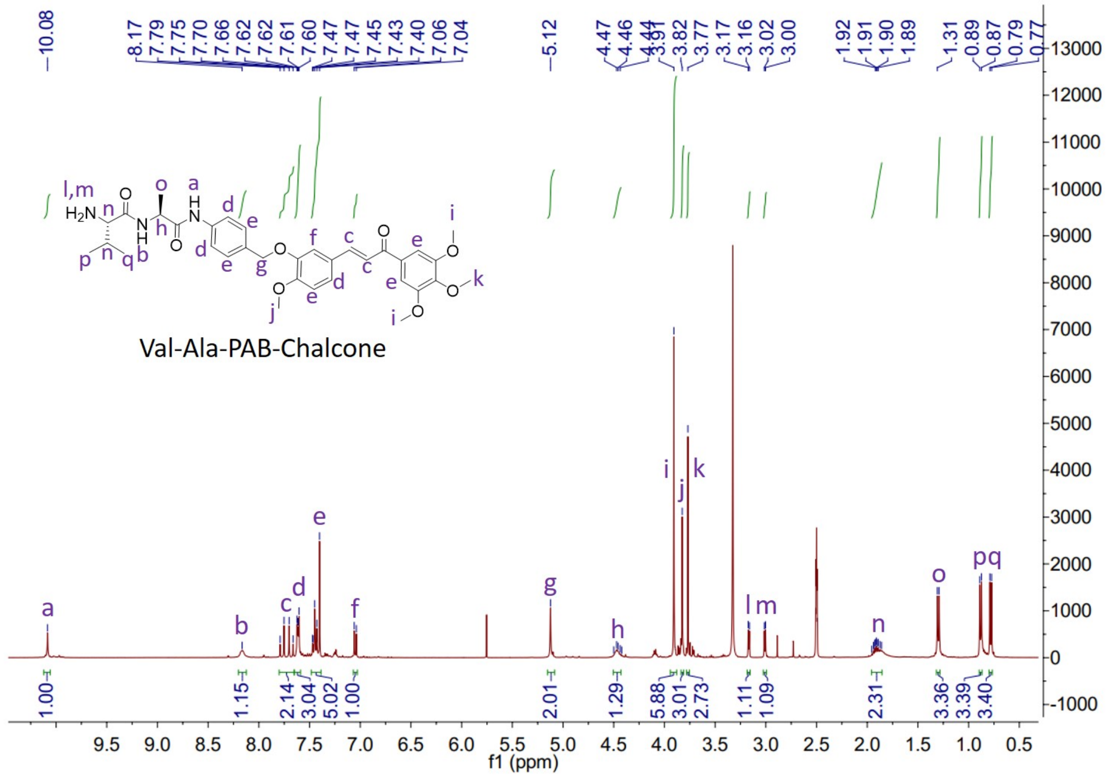

**Figure S68.** $^{1}H$ NMR spectrum of Val-Ala-PAB-Chalcone.

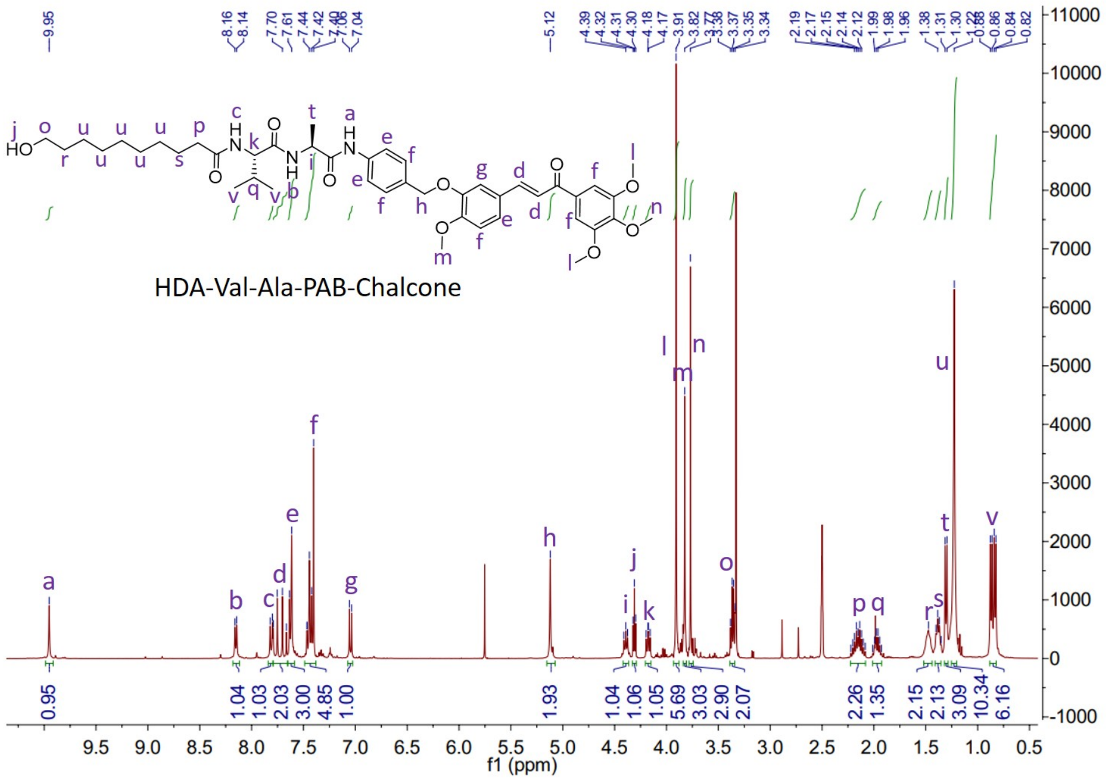


**Figure S69.** $^{1}H$ NMR spectrum of HDA-Val-Ala-PAB-Chalcone.

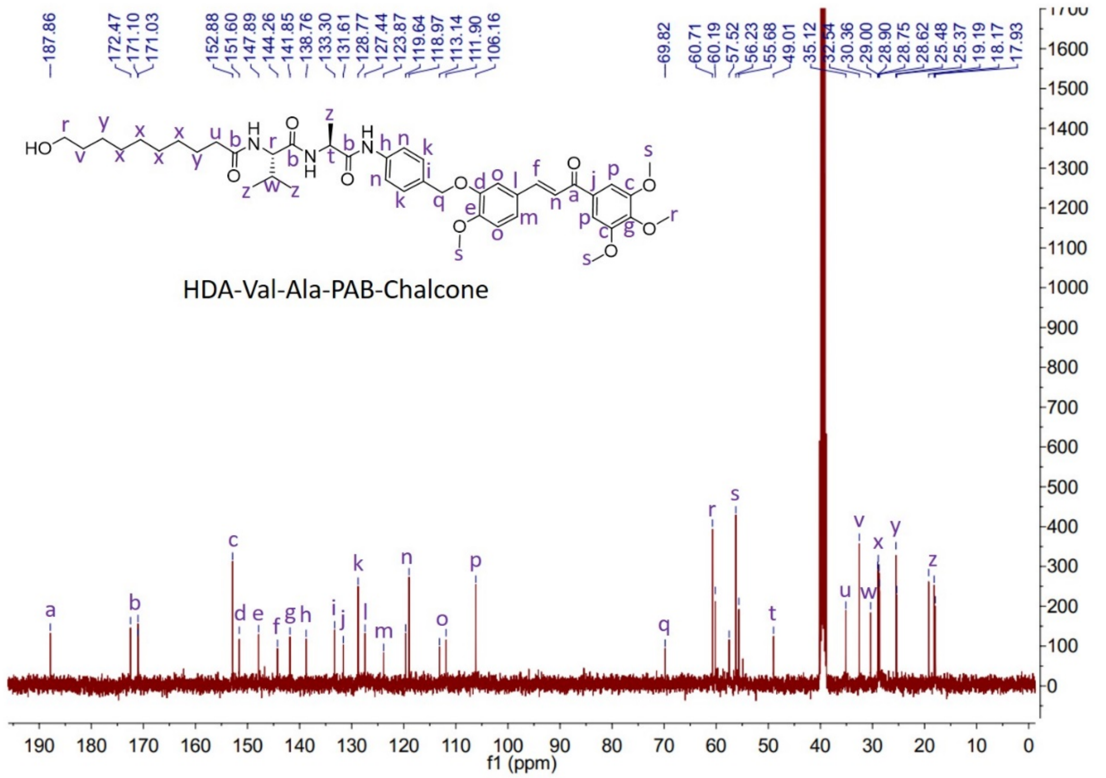


**Figure S70.** $^{13}C$ NMR spectrum of HDA-Val-Ala-PAB-Chalcone.

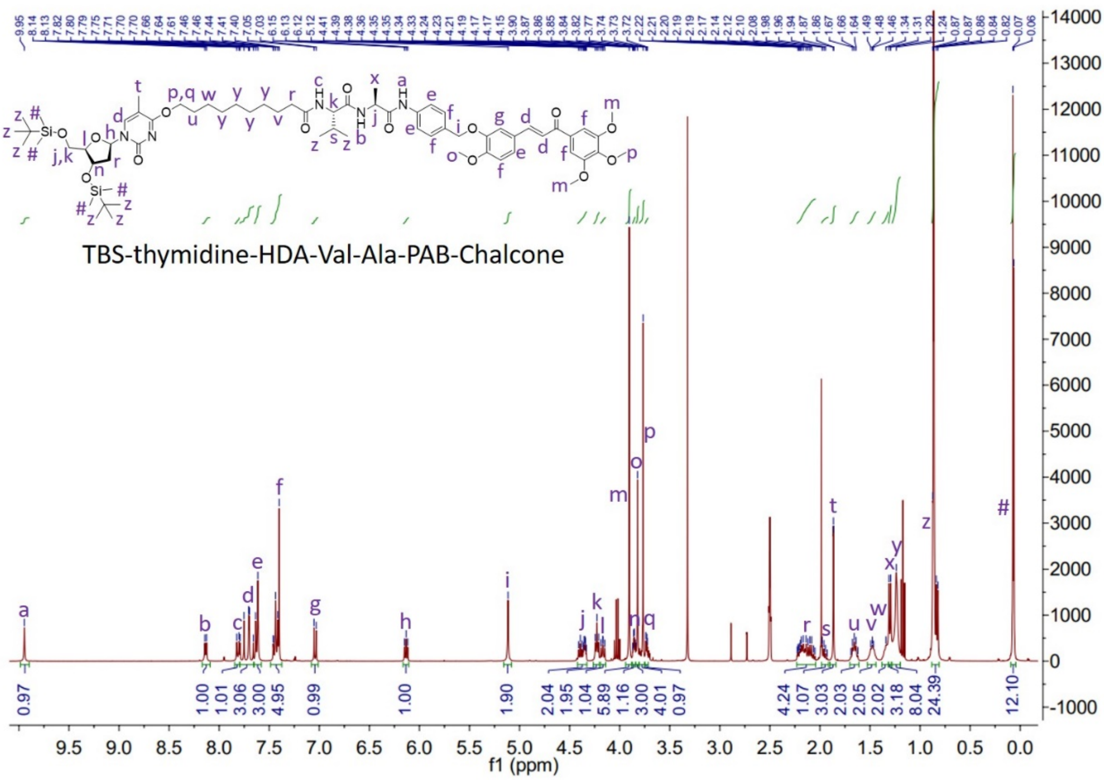


**Figure S71.** $^{1}$H NMR spectrum of TBS-Thymidine-HDA-Val-Ala-PAB-Chalcone.

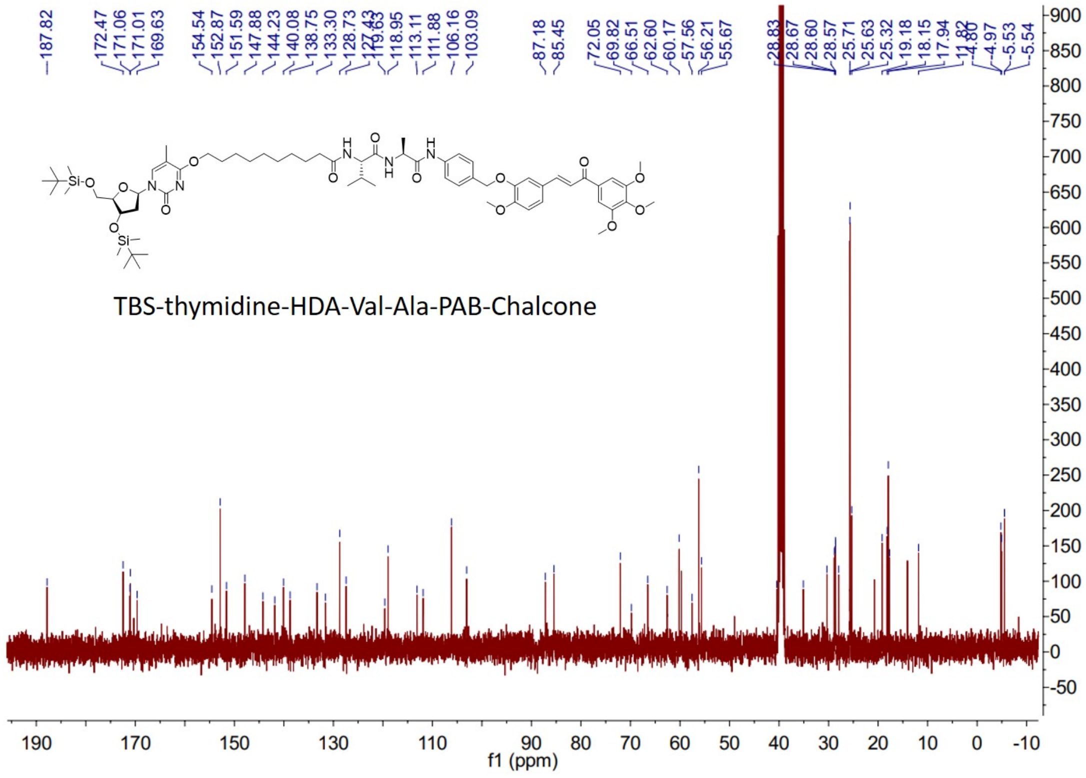


**Figure S72.** $^{13}$C NMR spectrum of TBS-Thymidine-HDA-Val-Ala-PAB-Chalcone.

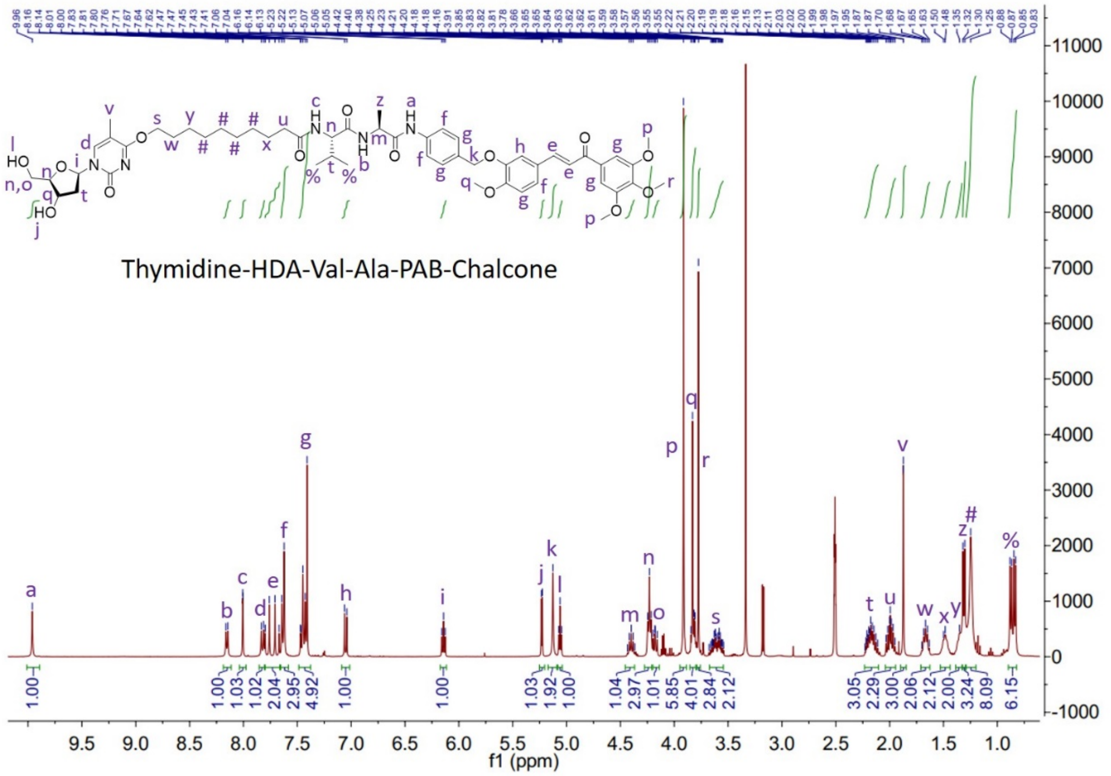


**Figure S73.** $^{1}H$ NMR spectrum of Thymidine-HDA-Val-Ala-PAB-Chalcone.

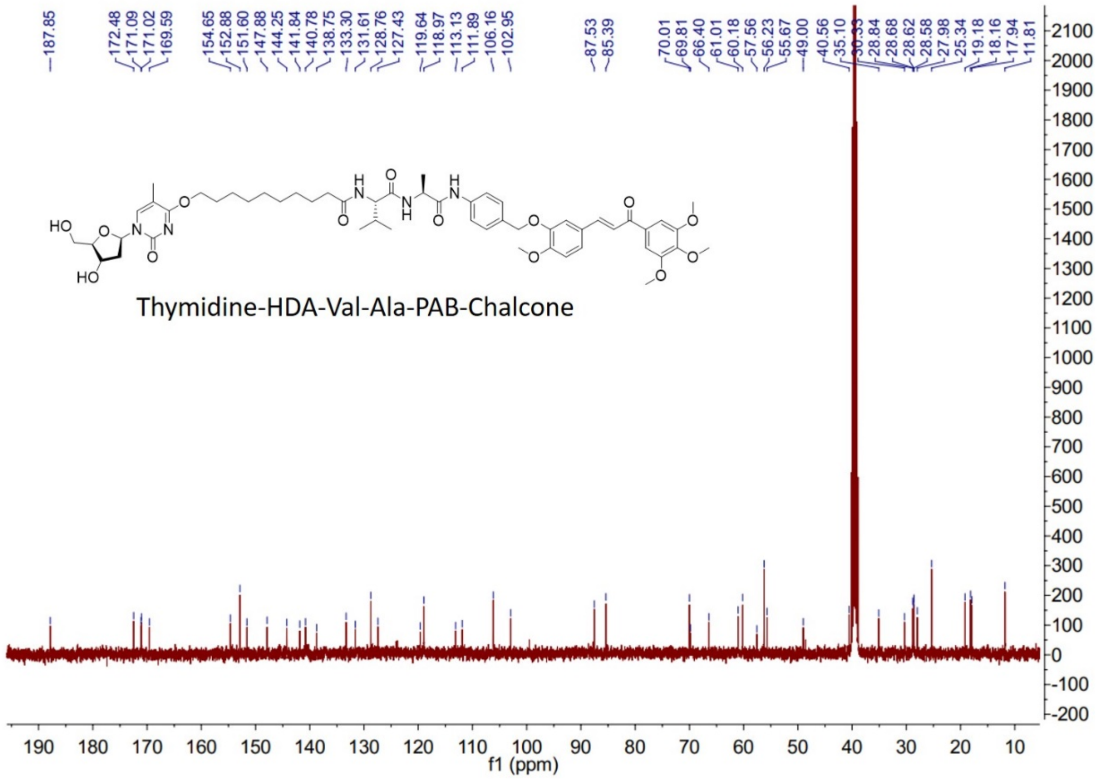


**Figure S74.** $^{13}C$ NMR spectrum of Thymidine-HDA-Val-Ala-PAB-Chalcone.

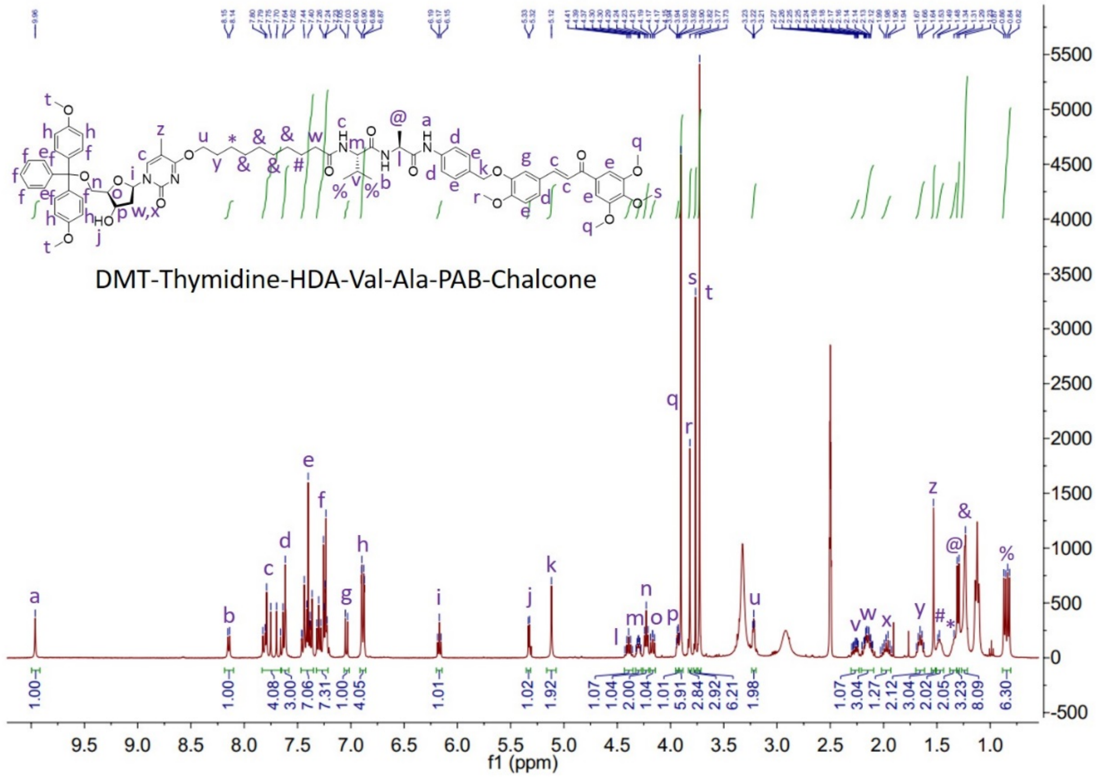


**Figure S75.** $^{1}$H NMR spectrum of DMT-Thymidine-HDA-Val-Ala-PAB-Chalcone.

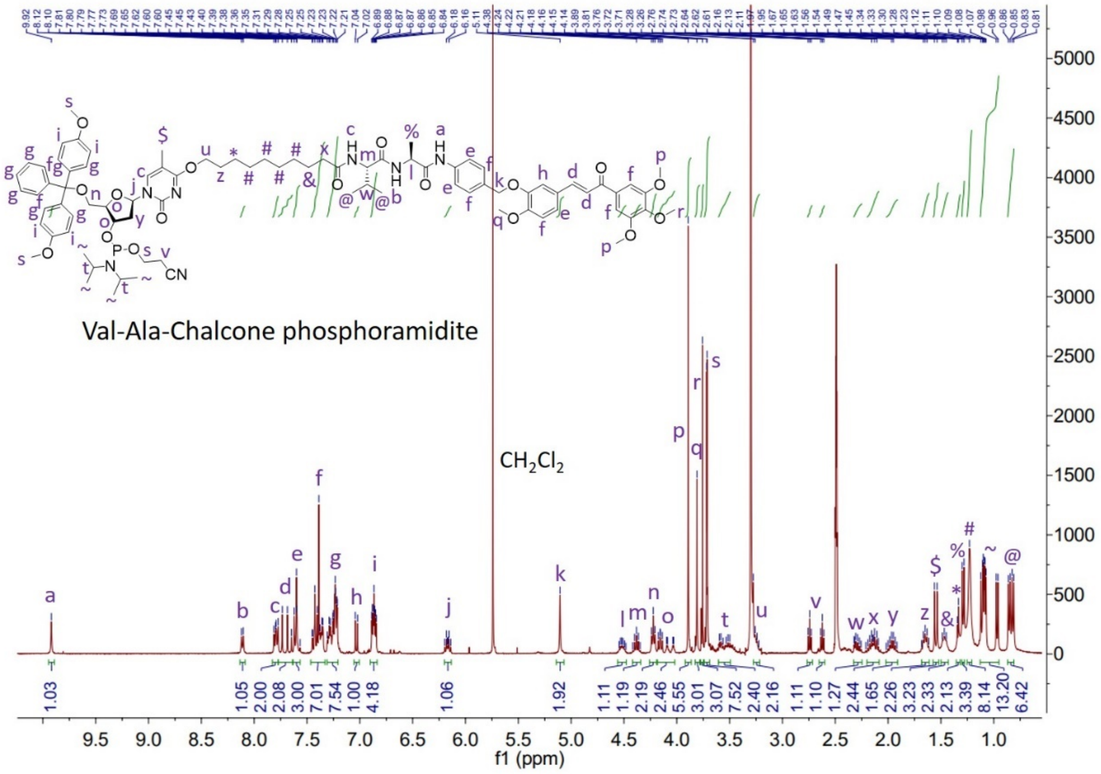


**Figure S76.** $^{1}$H NMR spectrum of Val-Ala-Chalcone phosphoramidite.

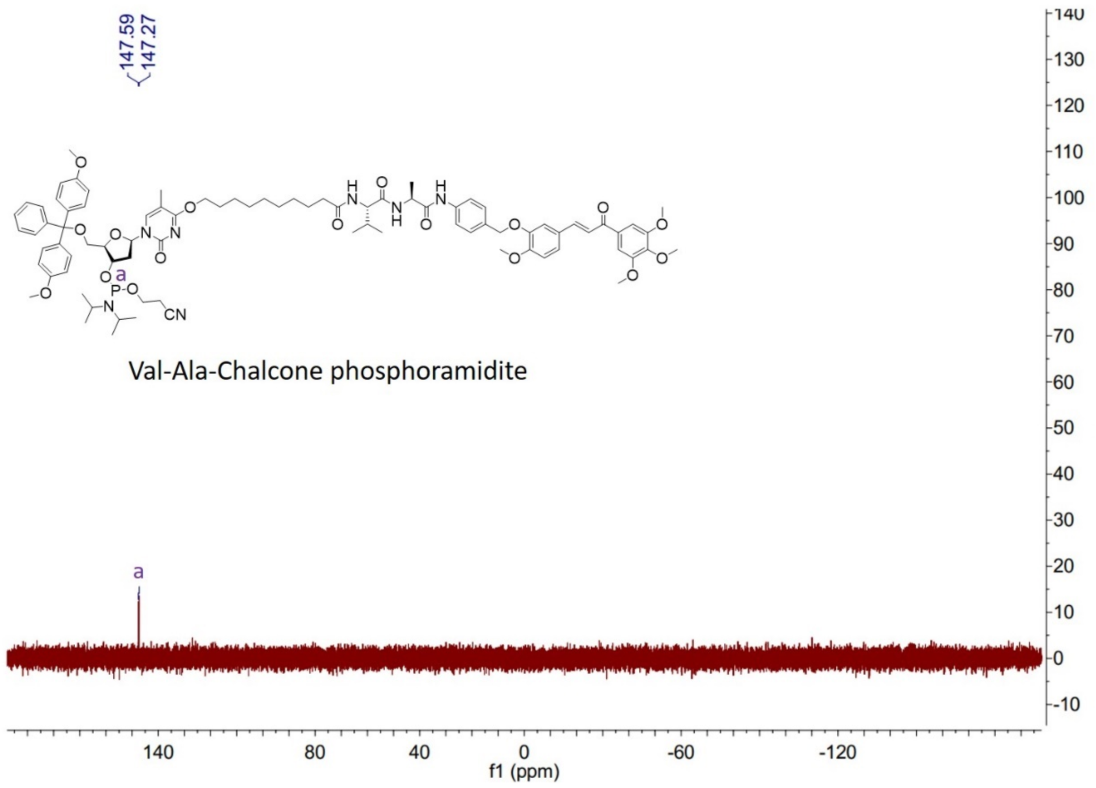


**Figure S77.** $^{31}$P NMR spectrum of Val-Ala-Chalcone phosphoramidite.

## 7. Author contributions

C.J., T.B. and W.T. put forward the concept. C.J. and T.B. designed the experiments. C.J. performed the experiments and collected the experimental data. A.H.E.-S. synthesized oligonucleotides. S.L. participated in performing the cell viability assay. C.J., T.B. and A.H.E.-S. analyzed the data. C.J. wrote the manuscript, and T.B., W.T., K.A.V. and A.H.E.-S. revised the manuscript.
